# Prevention of complications related to peripherally inserted central catheter insertion techniques in newborns: systematic review and network meta-analysis ^*^

**DOI:** 10.1590/1518-8345.6905.4161

**Published:** 2024-07-05

**Authors:** Ludmylla de Oliveira Beleza, Guilherme da Costa Brasil, Amanda Salles Margatho, Christiane Inocêncio Vasques, Renata Cristina de Campos Pereira Silveira, Priscilla Roberta Silva Rocha, Laiane Medeiros Ribeiro

**Affiliations:** 1 Universidade de Brasília, Brasília, DF, Brazil.; 2 Secretaria do Estado de Saúde, Hospital Materno Infantil de Brasília, UTI Neonatal, Brasília, DF, Brazil.; 3 Centro Universitário do Distrito Federal, Brasília, DF, Brazil.; 4 Universidade de São Paulo, Escola de Enfermagem de Ribeirão Preto, PAHO/WHO Collaborating Centre for Nursing Research Development, Ribeirão Preto, SP, Brazil.; 5 Universidade de Brasília, Faculdade de Ciências da Saúde, Brasília, DF, Brazil.; 6 Universidade de Brasília, Faculdade de Ceilândia, Brasília, DF, Brazil.

**Keywords:** Newborn Infant, Peripheral Catheterization, Central Venous Catheters, Treatment Failure, Neonatal Nursing, Neonatal Intensive Care Units

## Abstract

**Objective:**

to analyze the effectiveness of peripherally inserted central catheter insertion techniques in preventing the occurrence of complications related to this device in newborns.

**Method:**

a paired and network systematic literature review and meta-analysis, with its search carried out in seven databases and in the Grey Literature, including randomized and non-randomized clinical trials. The risk of bias was assessed using the Cochrane Risk of Bias 2 and Risk of Bias In Non-randomized Studies of Interventions tools. Certainty of the evidence was assessed by means of the Grading of Recommendations Assessment, Development and Evaluation. A meta-analysis was carried out with the aid of the R statistical program.

**Results:**

eight studies with 1,126 newborns were included and six insertion techniques were identified: intracavitary electrocardiogram; intracavitary electrocardiogram associated with ultrasound; ultrasound; formula; anatomical landmark; and modified anatomical landmark. Five techniques significantly decreased primary tip malpositioning when compared to the control ( *p* <0.05). Intracavitary electrocardiogram significantly and more effectively reduced arrhythmias, general complications and phlebitis; the technique that used a formula also reduced general complications. Infection, infiltration, secondary tip malpositioning, catheter rupture, thrombosis, occlusion and catheter-associated skin lesion were not significantly preventable events.

**Conclusion:**

intracavitary electrocardiogram and use of the formula were the most effective techniques in reducing complications.

## Highlights

**(1)** Some PICC insertion techniques have reduced their related complications.

**(2)** Primary tip malpositioning was the most prevented complication.

**(3)** Intracavitary electrocardiogram reduced arrhythmias, phlebitis and general complications.

**(4)** The technique that used the formula significantly reduced general complications.

## Introduction

 Peripherally Inserted Central Catheters (PICCs) are widely used in newborns (NBs) ^(^ ^1^ ^-^ ^2^ ^)^ . They have several advantages over the use of other central vascular access devices and short peripherally inserted catheters, such as preserving the peripheral vascular system ^(^ ^3^ ^)^ , reducing the risks of phlebitis and infections ^(^ ^3^ ^-^ ^4^ ^)^ , of the need for repeated punctures ^(^ ^4^ ^-^ ^5^ ^)^ and of the pain and stimuli to which NBs are subjected ^(^ ^3^ ^-^ ^5^ ^)^ . 

 Despite the advantages, PICC use is associated with different types of complications that can increase neonatal morbidity and mortality ^(^ ^6^ ^-^ ^7^ ^)^ , increase costs for the health system and compromise the patients’ quality of life ^(^ ^8^ ^-^ ^10^ ^)^ . 

 PICC-related complications can occur since their insertion, passing through the dwell period and even after their removal ^(^ ^6^ ^)^ . According to the Infusion Nursing Society (INS) (2021), the complications related to the insertion of central vascular access devices such as PICCs are as follows: arterial puncture; cardiac arrhythmias; nerve injury; air embolism; and primary malpositioning of the central vascular access device ^(^ ^11^ ^)^ . Many complications such as catheter-associated deep vein thrombosis, secondary malpositioning (intra- or extra-vascular catheter tip migration), central line-associated bloodstream infection, phlebitis, infiltration, leaks, catheter rupture, occlusion and device-associated skin lesion might be prevented depending on the insertion technique used ^(^ ^11^ ^)^ . 

 From the point of view of the insertion technique, some precautions such as the method used to guide the insertion interfere with PICC-related complications, that is, actions performed during insertion of this device can prevent complications related to its introduction and the late ones that occur with permanence of the PICC ^(^ ^11^ ^)^ . 

 Some studies and sets of good practices recommend using technologies in PICC insertion in order to prevent related complications and increase patient safety, such as ultrasound (USG) ^(^ ^11^ ^-^ ^12^ ^)^ , catheter-vein ratio ^(^ ^11^ ^)^ and intracavitary electrocardiogram (IC-ECG) ^(^ ^11^ ^,^ ^13^ ^-^ ^14^ ^)^ . For health institutions, there is a reduction in costs related to the treatment of complications since, in some situations, removal of this device requires another puncture and, oftentimes, it is necessary to use new catheters, in addition to the increase in the nursing time spent ^(^ ^5^ ^,^ ^10^ ^-^ ^11^ ^)^ . 

 USG can be used to determine the optimal size of the catheter to be introduced according to the diameter of the vein (catheter-vein ratio), to locate the tip, and for vascular visualization to guide the puncture. This can reduce complications and the need for repositioning, as well as increase the success chances at the first puncture attempt and provide greater patient satisfaction ^(^ ^11^ ^-^ ^12^ ^,^ ^15^ ^)^ . 

 Through electrodes on the body surface and in contact with the catheter, an IC-ECG monitors changes in the size of the electrocardiogram P wave, whose elevation indicates that the catheter tip reached the cavoatrial junction through the superior vena cava ^(^ ^14^ ^)^ . This technique strains, in real time, the correct position of the tip of the device, leading to significantly more successful central positions due to its immediate adjustment, in addition to providing immediate treatment and eliminating the need to confirm the tip position by means of a radiological examination ^(^ ^13^ ^-^ ^14^ ^,^ ^16^ ^)^ . 

Review studies on the relationship among all insertion techniques and PICC-related complications in NBs were not identified in the literature or on the registration platforms of this review.

 For this reason, this study aimed at analyzing the effectiveness of peripherally inserted central catheter insertion techniques in preventing the occurrence of complications related to this device in newborns. A network meta-analysis was used to achieve this objective, as it allows for a comparison among three or more concurrent interventions simultaneously in a single analysis, combining direct evidence (studies in which the interventions of interest are compared) and indirect evidence (studies in which the interventions of interest with a common comparator are compared). The effect estimates from direct and indirect comparison studies are combined into a network of interventions ^(^ ^17^ ^)^ . 

## Method

 This is a systematic literature review with paired and network meta-analysis, reported according to the PRISMA (Preferred Reporting Items Systematic Reviews and Meta-Analysis Checklist) ^(^ ^18^ ^)^ and PRISMA-Network Meta-Analyses (PRISMA-NMA) ^(^ ^19^ ^)^ guidelines. The protocol was registered on the PROSPERO platform under number CRD42022324152. 

 The PICOS ^(^ ^17^ ^)^ (acronym for Population, Interventions, Comparators, Outcomes, and Study design) strategy was used to delimit the guiding question of this review: In newborn patients (P), which PICC insertion technique (I) is most effective in preventing the occurrence of complications related to the use of this device (O)? 

### Eligibility criteria

Randomized and non-randomized controlled trials were included, which evaluated PICC insertion techniques in hospitalized newborns and PICC-related complications referred to as outcome measures.

The studies excluded were those that evaluated complications related to PICC use in NBs receiving outpatient care; as well as those that evaluated complications related to PICC use in newborns in Mixed Intensive Care Units (they care for newborns together with children); those that evaluated complications related to PICC use in children and adults; those that did not describe the insertion techniques; those that evaluated different types of PICCs, insertion sites or dressings; those that evaluated insertion techniques for other types of central vascular access devices; observational studies; qualitative studies; conference abstracts, reviews, editorials, experts’ opinions, case reports and series, reflection articles; and studies without a comparison group.

### Information sources

 The search was carried out in the following electronic databases: Cumulative Index to Nursing and Allied Health Literature (CINAHL), Cochrane CENTRAL, Embase, Latin American and Caribbean Literature in Health Sciences (LILACS), PubMed, Scopus and Web of Science. The grey literature was consulted through the Google Scholar and Open Gray databases. The reference lists of the studies included were also consulted in order to identify any publications not previously identified in the searches. In addition to that, there was an active search for publications that might comprise the sample for this review through consultation with experts. The search was carried out on January 1 ^st^ , 2023. 

### Search strategy

The search strategy was developed based on the guiding question of this review, using the “OR” Boolean operator between synonyms and similar terms and “AND” between keywords. Initially, the strategy was structured for the PubMed database, using the Medical Subject Headings (MeSH) descriptors and keywords, and then adapted for each of the databases searched. There were no time or language restrictions on the publications.

The search strategy developed in the PubMed database was as follows: (“infant, newborn”[MeSH Terms] OR “Newborn Infant”[All Fields] OR “Newborn Infants”[All Fields] OR “Newborns”[All Fields] OR “Newborn”[All Fields] OR “Neonate”[All Fields] OR “Neonates”[All Fields] OR “infant, premature”[MeSH Terms] OR “Premature Infant”[All Fields] OR “Preterm Infants”[All Fields] OR “Preterm Infant”[All Fields] OR “Premature Infants”[All Fields] OR “Neonatal Prematurity”[All Fields] OR “Preterm”[All Fields] OR “infant, extremely premature”[MeSH Terms] OR “Extremely Premature”[All Fields] OR “Extremely Premature Infant”[All Fields] OR “Extremely Preterm Infants”[All Fields] OR “Extremely Preterm Infant”[All Fields] OR “Extremely Premature Infants”[All Fields] OR “infant, extremely low birth weight”[MeSH Terms] OR “Extremely Low Birth Weight Infant”[All Fields] OR “Extremely Low Birth Weight Infants”[All Fields] OR “infant, very low birth weight”[MeSH Terms] OR “very low birth weight infant”[All Fields] OR “very low birth weight infants”[All Fields] OR “very low birth weight infant”[All Fields] OR “very low birth weight infants”[All Fields] OR “Very Low Birth Weight”[All Fields] OR “infant, low birth weight”[MeSH Terms] OR “low birth weight infant”[All Fields] OR “low birth weight infants”[All Fields] OR “low birth weight infant”[All Fields] OR “low birth weight infants”[All Fields] OR “Low Birth Weight”[All Fields] OR “Low Birth Weights”[All Fields] OR “infant, small for gestational age”[MeSH Terms] OR “Small for Gestational Age”[All Fields] OR “Term Birth”[MeSH Terms] OR “Term Births”[All Fields] OR “Fullterm Birth”[All Fields] OR “Fullterm Births”[All Fields] OR “infant, postmature”[MeSH Terms] OR “Postmature Infant”[All Fields] OR “Postmature Infants”[All Fields] OR “Neonatal”[All Fields] OR “intensive care units, neonatal”[MeSH Terms] OR “Neonatal Intensive Care Unit”[All Fields] OR “Neonatal Intensive Care Units”[All Fields] OR “Neonatal Intensive Care”[All Fields] OR “Neonatal ICU”[All Fields] OR “Neonatal ICUs”[All Fields]) AND (“catheterization, peripheral”[MeSH Terms] OR “Peripherally Inserted Central Catheter”[All Fields] OR “Peripherally Inserted Central Catheters”[All Fields] OR “PICC”[All Fields] OR “PICCs”[All Fields] OR “Peripherally Inserted Central Catheter Line”[All Fields] OR “Peripherally Inserted Central Catheter Lines”[All Fields] OR “PICC Line”[All Fields] OR “PICC Lines”[All Fields]) AND (“Complications”[MeSH Subheading] OR “Complication”[All Fields] OR “Treatment Failure”[MeSH Terms] OR “Treatment Failures”[All Fields] OR “PICC Failure”[All Fields] OR “PICC Complication”[All Fields] OR “PICC Complications”[All Fields] OR “Infections”[All Fields] OR “Infection”[All Fields] OR “Catheter-Related Infections”[MeSH Terms] OR “catheter related infection”[All Fields] OR “catheter related infection”[All Fields] OR “Central Line-Associated Bloodstream Infection”[All Fields] OR “CLABSI”[All Fields] OR “Central Line-Associated Bloodstream Infections”[All Fields] OR “Bloodstream Infection”[All Fields] OR “Bloodstream Infections”[All Fields] OR “Catheter-Associated Bloodstream”[All Fields] OR “Catheter-Associated Bloodstream Infections”[All Fields] OR “Central Line Bloodstream Infection”[All Fields] OR “CABSI”[All Fields] OR “CRBSI”[All Fields] OR “Neonatal Nosocomial Infections”[All Fields] OR “Neonatal Nosocomial Infection”[All Fields] OR “Deep Vein Thrombosis”[All Fields] OR “Catheter-Related Deep Vein Thrombosis”[All Fields] OR “Thrombosis”[MeSH Terms] OR “Thromboses”[All Fields] OR “Thrombus”[All Fields] OR “Blood Clot”[All Fields] OR “Blood Clots”[All Fields] OR “Phlebitis”[MeSH Terms:noexp] OR “Phlebitides”[All Fields] OR “Periphlebitis”[All Fields] OR “Adverse Effects”[MeSH Subheading] OR “Adverse Effect”[All Fields] OR “Pericardial effusion”[All Fields] OR “Cardiac Tamponade”[All Fields] OR “Arterial Puncture”[All Fields] OR “Nerve Injury”[All Fields] OR “Nerve Injuries”[All Fields] OR “embolism, air”[MeSH Terms] OR “Air Embolism”[All Fields] OR “Air Embolisms”[All Fields] OR “arrhythmias, cardiac”[MeSH Terms] OR “Cardiac Dysrhythmia”[All Fields] OR “Cardiac Dysrhythmias”[All Fields] OR “Arrhythmia”[All Fields] OR “Arrythmia”[All Fields] OR “Cardiac Dysrhythmia”[All Fields] OR ((“central”[All Fields] OR “centrally”[All Fields] OR “centrals”[All Fields]) AND (“vascular access devices”[MeSH Terms] OR (“vascular”[All Fields] AND “access”[All Fields] AND “devices”[All Fields]) OR “vascular access devices”[All Fields] OR (“vascular”[All Fields] AND “access”[All Fields] AND “device”[All Fields]) OR “vascular access device”[All Fields]) AND (“malposition”[All Fields] OR “malpositioned”[All Fields] OR “malpositioning”[All Fields] OR “malpositionings”[All Fields] OR “malpositions”[All Fields])) OR “Infiltration”[All Fields] OR “Extravasation”[All Fields] OR “Extravasation of Diagnostic and Therapeutic Materials”[MeSH Terms]).

### Selection process

 After removing duplicates using the Endnote Web software (Clarivate Analytics) ^(^ ^20^ ^)^ , the lists of references of the studies identified in the databases were exported to the Rayyan online software (Rayyan *,* Qatar Computing Research Institute) ^(^ ^21^ ^)^ . 

The study selection process was carried out in two phases. In the first one, two reviewers (L.O.B.; G.B.) independently read the titles and abstracts of the studies identified in the databases, applying the eligibility criteria. In Phase 2, the reviewers (L.O.B.; G.B.) again applied the inclusion criteria after reading the studies in full. The disagreements were solved through discussion between them, and a third reviewer (A.S.M.) was only resorted to when they did not reach a consensus.

### Data collection process

Both reviewers (L.O.B.; G.B.) extracted the following data from the studies selected in phase 2: characteristics of the study (author, year of publication, country, study objectives and main conclusions) and of the sample (number of NBs and catheters gestational age and birth weight, corrected gestational age or days of life and weight at the time of PICC insertion), insertion technique performed and comparator, primary and secondary outcomes.

 The data collected were organized in Microsoft Word ^®^ tables and the outcomes were entered into Microsoft Excel ^®^ by a reviewer, with independent cross-checking by another. Subsequently, the data were transported to the R software, version 4.2.3. 

### Outcomes

 The rates of general and specific PICC-related complications were considered as primary outcomes. The definition of complications was based on the terms described in the INS set of good practices (2021), as well as on what it considers as complications related to the insertion technique: arterial puncture, cardiac arrhythmias, nerve injury, air embolism and primary malpositioning of central vascular access devices. The catheter-related complications were catheter-associated deep vein thrombosis, secondary malpositioning (intra- or extra-vascular catheter tip migration), phlebitis, infiltration and extravasation, central line-associated bloodstream infection, occlusion, catheter rupture, and catheter-associated skin lesion ^(^ ^11^ ^)^ . These latter complications that occur during PICC permanence were added because they have been described as preventable depending on the insertion technique used ^(^ ^11^ ^)^ . In addition to that, as only studies with changes exclusively in the insertion technique between the groups monitored were included, with no difference in the material used or in the PICC care and maintenance procedures, it can be stated that these outcomes also analyzed by the authors in these studies would only be related to the insertion techniques used. The different nomenclatures used by the authors of the studies to define the complications were standardized according to the description proposed in the INS set of good practices ^(^ ^11^ ^)^ . 

The secondary outcomes collected from the studies that had these data were as follows: Overall Success Rate (OSR) or number of PICCs successfully introduced in all insertion attempts; success rate of the first puncture; number of punctures; and catheter dwell time.

### Risk of bias corresponding to the studies included

 The risk of bias assessment of the randomized clinical trials included was performed by two reviewers, using the Risk of Bias 2 (RoB2) tool ^(^ ^17^ ^)^ developed by the Cochrane Collaboration. In turn, the non-randomized clinical trials were evaluated using the Risk of Bias In Non-Randomised Studies of Interventions (ROBINS-I) tool, also developed by the Cochrane Collaboration ^(^ ^22^ ^)^ . 

### Effect measures

The data extracted and the results found were expressed by means of relative and absolutes frequencies or through mean values and standard deviations. For binary outcomes, the effect measure used was Relative Risk (RR) and, for the continuous outcome, the Means Difference (MD). All results were reported with their respective 95% confidence intervals (CIs). The figures and graphs were performed in the statistical program (R Statistics Software).

### Synthesis methods

 The outcome data were transported to the R Statistics Software, version 4.2.3, using the meta version 6.2-1 and netmeta version 2.8-1 packages (The R Foundation, Vienna, Austria). For each outcome analyzed, a different number of studies directly compared new insertion techniques (IC-ECG, formula, etc.) to the puncture technique guided by anatomical landmarks (common comparator). An intervention meta-analysis was carried out with the studies considered homogeneous regarding study design, characteristics of the sample and insertion technique performed, in which a ≤0.05 significance level was considered. The common comparator found in each study allowed for a simultaneous comparison of all treatments of interest (insertion techniques) through a network meta-analysis. The analyses were performed according to the frequentist approach, using the random effects model, with the Mantel-Haenzel method for binary outcomes and the inverse variance method for the continuous outcome. Heterogeneity was evaluated according to I ^2^ , based on the classification suggested by PRISMA ^(^ ^18^ ^)^ . 

 To sort the treatments evaluated in each network meta-analysis into a ranking, the P-score was estimated, which is based on the point estimate and the standard error of each estimate included in the network meta-analysis and measures the magnitude of the certainty that one treatment is better than another. This score varies from 0 to 1, and values close to 1 indicate superiority of the treatment under evaluation ^(^ ^23^ ^)^ . 

 The studies carried out only with preterm infants were excluded from the network meta-analyses, as they deal with a peculiar and differentiated sample from those used in studies that combine preterm infants with full-term NBs, thus avoiding intransitivity ^(^ ^17^ ^)^ . Preterm infants have immature skin and vascular system anatomy and physiology, lower immunity, higher rates of infections and mechanical complications ^(^ ^4^ ^,^ ^24^ ^-^ ^28^ ^)^ and also a higher chance of proper tip positioning ^(^ ^29^ ^)^ . 

It is noted that the network meta-analysis had not been provided for in the protocol of this review; however, considering the feasibility of this analysis and the importance/relevance of the evidence it provides, it was decided to perform it. Such decision was based on the analysis of the data from the eligible studies, when presence of a control group in common among the insertion techniques evaluated in the included studies was verified. In addition to that, the network meta-analysis is the method that best answers the guiding question of this review.

### Evaluation of publication bias

 A search on Clinicaltrials.gov was carried out for unpublished results of clinical trials as a way to reduce the risk of bias due to lack of results in a synthesis ^(^ ^17^ ^)^ . It was not possible to generate the funnel plot because this feature requires a minimum of 10 tests to ensure adequate power to detect asymmetries ^(^ ^17^ ^)^ . 

### Certainty of the evidence assessment

 The Grading of Recommendations, Assessment, Development, and Evaluation Working Group (GRADE) approach was used to assess the quality and certainty of the evidence for each outcome of each comparison ^(^ ^30^ ^)^ , with downgrading or elevation by one or two points based on judgment criteria. The web version of GRADEpro was used to present the conclusions for the main outcomes of the direct comparisons (GRADEpro GDT; GRADE Working Group, 2022). The evaluation regarding certainty of the evidence of the effect estimates found in the network meta-analysis was carried out in four stages with the aid of a specific form prepared in Microsoft Excel ^®^ : 1) Presentation of the direct and indirect estimates of each comparison of interest; 2) Classification of the certainty of the evidence of each direct and indirect estimate; 3) Presentation of the effect estimates corresponding to the network meta-analysis of the comparison of interest; and 4) Classification of the certainty of the evidence corresponding to the network meta-analysis estimates ^(^ ^31^ ^)^ . In these stages, intransitivity of the indirect estimates, incoherence (or inconsistency, as PRISMA calls it) and imprecision of the network were evaluated ^(^ ^32^ ^-^ ^34^ ^)^ . 

## Results

**Figure 1:**
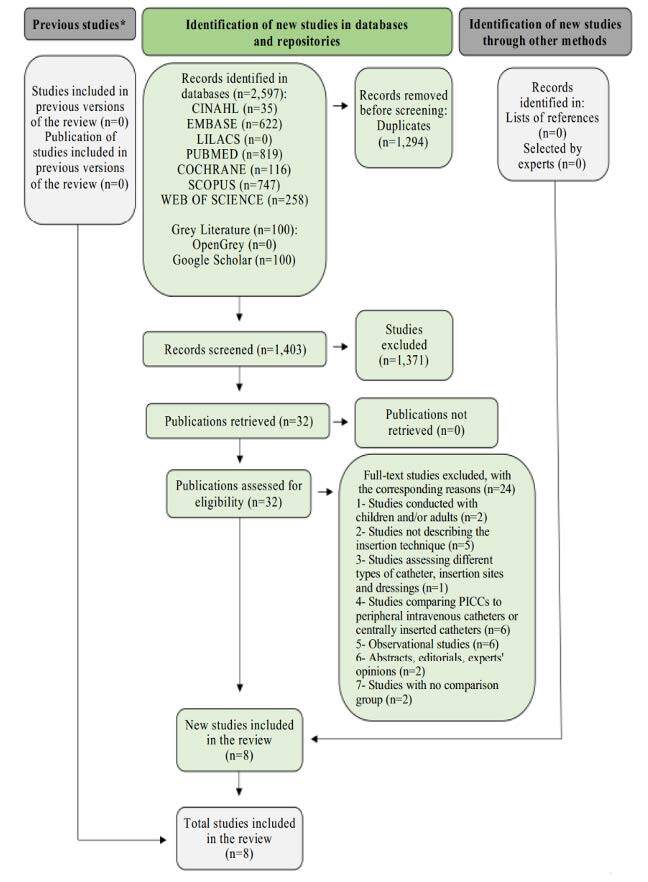
- Flowchart corresponding to the literature search and selection criteria adapted from PRISMA ^(^ ^18^ ^)^

 A total of 2,697 studies were identified in the databases and grey literature, of which 1,294 were duplicates, resulting in 1,403 articles selected for reading their titles and abstracts. A detailed flowchart of the selection, exclusion and inclusion of the studies, adapted from PRISMA, is shown in Figure 1 . It should be added that seven of the 100 records selected in the Grey Literature were duplicates; the others were screened for titles and abstracts, but none were included in this review. 

*CINAHL = Cumulative Index to Nursing and Allied Health Literature; ^†^ LILACS = Latin American and Caribbean Literature in Health Sciences

 Eight studies were included in this review, with a total sample of 1,126 NBs. Five were from China ^(^ ^14^ ^,^ ^28^ ^,^ ^35^ ^-^ ^37^ ^)^ , one from the United States ^(^ ^38^ ^)^ , one from India ^(^ ^39^ ^)^ and one from Brazil ^(^ ^40^ ^)^ . The publication period ranged from 2013 to 2021, and one study was only available in the Chinese language ^(^ ^35^ ^)^ . 

 Regarding the study designs, six were randomized controlled trials ^(^ ^14^ ^,^ ^35^ ^-^ ^36^ ^,^ ^38^ ^-^ ^40^ ^)^ and two were non-randomized trials ^(^ ^28^ ^,^ ^37^ ^)^ . 

 The samples varied within the neonate population, three studies only evaluated the insertion techniques in preterm ^(^ ^28^ ^)^ and very preterm/extreme preterm ^(^ ^38^ ^-^ ^39^ ^)^ NBs and four assessed the insertion techniques in preterm and full-term NBs ^(^ ^14^ ^,^ ^35^ ^,^ ^37^ ^,^ ^40^ ^)^ . One study only cited the mean weight of 2,500 g on the insertion day ^(^ ^36^ ^)^ . 

 The PICC insertion techniques in NBs found in the studies included were the following: intracavitary electrocardiogram (IC-ECG) to guide the tip ^(^ ^14^ ^,^ ^35^ ^)^ ; intracavitary electrocardiogram with ultrasound for vascular visualization (IC-ECG/USG) ^(^ ^28^ ^,^ ^37^ ^)^ ; real-time ultrasound (USGRT) to visualize the tip during insertion and guiding it ^(^ ^38^ ^-^ ^39^ ^)^ ; modified anatomical landmark (AL), in which the intervention took place with measurement of the catheter size to be inserted with a different anatomical framework ^(^ ^40^ ^)^ ; formula use, in which an equation determines the catheter length that must be inserted associated with performing a warm compress for 15 minutes at the insertion site before the puncture ^(^ ^36^ ^)^ ; and puncture guided by anatomical landmarks, characterized by blind puncture (without the aid of technologies), measurement by arm (the number of catheter centimeters to be inserted is predetermined by a measurement performed on the body surface from the probable insertion site to the superior or inferior vena cava) and visualization of the tip by x-ray ^(^ ^14^ ^,^ ^28^ ^,^ ^35^ ^-^ ^40^ ^)^ , which was the control group of all studies. In other words, the comparator/control in all studies was the same technique, which led the network to a star-shaped geometry. This means that all new techniques were compared to a common comparator, without direct comparison between them, but indirect. 

**Figure 2:**
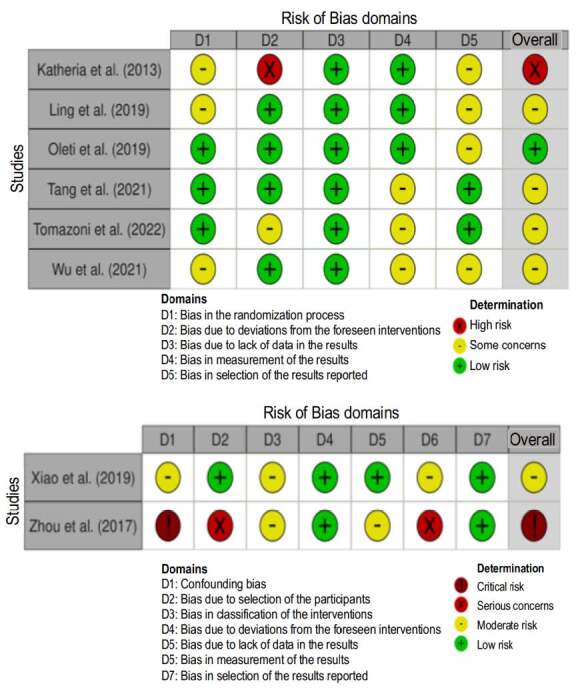
- Risk of bias assessment applied to the studies using the RoB2 (Risk of Bias 2) tool (above) and ROBINS-I (Risk of Bias In Non-randomized Studies of Interventions) (below). Brasília, DF, Brazil, 2023

 The risk of bias in the studies included is detailed in Figure 2 . 

 The randomized trials showed some concerns regarding the randomization process due to some differences in the characteristics of the participants ^(^ ^38^ ^)^ , lack of detailed information in the articles ^(^ ^36^ ^)^ or 1:1 allocation ^(^ ^14^ ^)^ . It was not possible for the professionals who applied the intervention to be blinded, as they would need to know what the intervention was to apply it, with the exception of one study ^(^ ^40^ ^)^ . In a research study that used USGRT ^(^ ^38^ ^)^ , some deviations from the intended interventions that may have affected the outcomes were observed, with unexplained insertion failures that can be directly related to the intervention, even changing the number of participants in both groups. Although the study protocol of one of the publications was not identified ^(^ ^39^ ^)^ , which led the bias domain of the reported results to be judged as “some concerns”, this study was evaluated as low risk of bias because it was well designed and contained the authors’ statement that all interventions and analyses were carried out according to the plan before initiating the study. Two publications did not report whether or not those responsible for evaluating the outcomes knew which group they were evaluating ^(^ ^35^ ^-^ ^36^ ^)^ . 

 The non-randomized trials obtained all the information from their control group through retrospective data, and the articles had some questions from the domains answered as “not reported” ^(^ ^28^ ^,^ ^37^ ^)^ . For example, there was no information on whether the professionals who evaluated the outcomes knew which group the participants belonged to, which may have mainly influenced measurement of the primary tip malpositioning, the only complication measured in the second study ^(^ ^37^ ^)^ . This same study ^(^ ^37^ ^)^ was considered at critical risk of bias because there were differences in the characteristics and number of participants between the groups with no control of the measured confounding factors; in addition, the participants seemed to have had different follow-ups. 

 The characteristics of the primary studies included in the review and their results are described in Figure 3 . 

**Figure 3:** - Characteristics of the studies included and individual results found (n=8). Brasília, DF, Brazil, 2023

**Study and sample characteristics**					**Results**	**Main Conclusions**
**Author; Year; Country**	**Interventions (Insertion and Control Technique)**	**Sample (n)**	**GAB** ^*^ **(s** ^†^ **) and BW** ^§^ **(g** ^||^ **)**	**CGA** ^‡^ **(s** ^†^ **) or days old and/or weight (g** ^||^ **)**	**Complications** **n (%) experimental x n (%) control**	
Katheria, et al.; 2013 ^(^ ^38^ ^)^ ; United States	Experimental Group: blind puncture, measurement by anatomical landmark, USGRT ^**^ for tip verification	20		CGA ^‡^: 31 ± 4 w ^†^ Weight: 1,185 ± 538 g ^||^ 4 ±5 days	1 ^st¶^ Malpositioning: 5(25%) **x** 16(57.1%) 2 ^nd††^ Malpositioning (Cardiac tamponade): 1 (5%) **x** 0 (0%)	USGRT ^**^ reduced the insertion procedure time by 40% ( *p* =0.034), as well as the need for x-ray ( *p* =0.001) and for manipulations ( *p* =0.034).
	Control Group: puncture guided by anatomical landmarks	28		CGA ^‡^: 30 ± 4 w ^†^ Weight: 1.208 ± 481 g ^||^ 5 ±5 days		
Ling, et al.; 2019 ^(^ ^14^ ^)^ ; China	Experimental Group: blind puncture, measurement by anatomical landmark, IC-ECG ^‡‡^ to guide the tip	80	GAB ^*^: 37.1± 1.4 w ^†^ (32-40)	3.4 ± 0.5 days (1-6)	General complications: 3(3.75%) **x** 19(23.75%), *p* =0.000 Arrhythmia: 0(0%) **x** 4(5%), *p* =0.043 Phlebitis: 1(1.25%) **x** 7(8.75%), *p* =0.03 Infection: 0(0%) **x** 1(1.25%), *p* =0.316 Skin lesion: 1(1.25%) **x** 1(1.25%), *p* =0.316 1 ^st¶^ Malpositioning: 4(5%) **x** 17(21.25%), *p* =0.002 Occlusion: 1(1.25%) **x** 4(5%), *p* =0.173	IC-ECG ^‡‡^ improved the rate of the first attempt to insert the PICC ^§§^ up to the central position; it also required less time and medical cost, less exposure to radiation and reduced complication rates.
	Control Group: puncture guided by anatomical landmarks	80	GAB ^*^: 36.8± 1.3 w ^†^ (32-40)	3.2 ± 0.3 days (1-5)		
Oleti, et al.; 2019 ^(^ ^39^ ^)^ ; India	Experimental Group: blind puncture, measurement by anatomical landmark, USGRT ^**^ for tip verification	40	GAB ^*^ **:** 31.1± 3.1 w ^†^ BW ^§^: 1,286 g ^||^ (926-1,662)	1.75 days (0.83-5.37)	Infection: 4(10.2%) **x** 1(2.5%) - RR ^||||^ 4.1; 95% CI ^¶¶^: 0.47-35, *p* =0.24 Infiltration or occlusion: 4(10%) **x** 6(15%) 1 ^st¶^ Malpositioning: 13(32.5%) **x** 27(67.5%) – RR ^||||^ 0.48; 95% CI ^¶¶^: 0.29-0.79; *p* =0.002	The use of USGRT ^**^ reduced the incidence of tip malpositioning by 52%, and can be recommended for PICC ^§§^ insertion.
	Control Group: puncture guided by anatomical landmarks	40	GAB ^*^: 31.4 ± 3.6 w ^†^ BW ^§^: 1,061 g ^||^ (889-1,636)	1.04 days (0.77-4.87)		
Tang, et al.; 2021 ^(^ ^35^ ^)^ ; China	Experimental Group: blind puncture, measurement by anatomical landmark, IC-ECG ^‡‡^ to guide the tip	105	GAB ^*^: 36.9 ± 1.3 w ^†^ (33-40)		General complications: 4(3.8%) **x** 23(21.9%); *p* =0.000 Arrhythmia: 0(0%) **x** 6(5.7%); *p* =0.015 Phlebitis: 2(1.9%) **x** 9(8.6%); *p* =0.029 Infection: 0(0%) **x** 1(1%); *p* =1 Skin lesion: 1(1%) **x** 3(2.9%); *p* =0.311 Thrombosis: 1(1%) **x** 4(3.8%); *p* =0.184	The use of IC-ECG ^‡‡^ helps to timely detect and correct ectopic tip and the resulting vascular injury, infection and arrhythmia, bringing greater safety than control.
	Control Group: puncture guided by anatomical landmarks	105	GAB ^*^: 36.4 ± 1.3 w ^†^ (32-40)			
Tomazoni, et al.; 2022 ^(^ ^40^ ^)^ ; Brazil	Experimental Group: blind puncture, measurement using a modified anatomical reference landmark (insertion site up to the right sternoclavicular junction), x-ray to check the tip	44	GAB ^*^ and BW ^§^ are stratified into bands		General complications: 17(38.6%) **x** 27(61.3%) Phlebitis: 1(2.3%) **x** 3(6.8%) Infiltration: 3(6.8%) **x** 6(13.6%) 1 ^st¶^ Malpositioning: 23(52.27%) **x** 43(97.7%); *p* =0.000 2 ^nd††^ Malpositioning: 1(2.3%) **x** 1(2.3%) Occlusion: 3(6.8%) **x** 4(9.1%) Rupture: 1(2.3%) **x** 1(2.3%) Suspected infection: 2(4.5%) x 1(2.3%)	Using the modified measurement method provided better results for adequate PICC ^§§^ tip positioning.
	Control Group: puncture guided by anatomical landmarks (insertion site up to the right sternoclavicular junction and up to the 3 ^rd^ intercostal space)	44				
Wu, et al.; 2021 ^(^ ^36^ ^)^ ; China	Experimental Group: saphenous puncture after 15 minutes of warm compress, guided by formula, x-ray to check the tip *Formula >1,500 g*: L ^***^ = M ^†††^ - 1.5 + 23 0.3 *Formula >1,500 g*: L ^***^ = 23 – 1.5 - M ^†††^ 0.3 L ^***^: Size of the PICC ^§§^ to be inserted M ^†††^: Newborn weight Control Group: saphenous vein puncture guided by anatomical landmarks	65		16.52± 0.63 days (1.47-26.46) Weight: 2,510 ± 180 g ^||^ (1,170-3,760)	General complications: 4(6.15%) **x** 23(35.38%); <0.001 Phlebitis: 1(1.53%) **x** 7(10.76%) Infection: 1(1.53%) **x** 6(9.23%) 2 ^nd††^ Malpositioning: 1(1.53%) **x** 4(6.15%) Thrombosis: 1(1.53%) **x** 6(9.23%) Note: The 1 ^st^ malpositioning ^st^ was not checked per individual, but regarding the number of centimeters that had to be pulled: Formula with traction of 0.25 ± 0.08 centimeters **x** Control with traction of 2.87 ± 0.23 centimeters ( *p* <0.000)	Using the formula to determine the catheter length to be inserted reduced hospitalization time, pain scale and related complications, as well as it increased the success rate of the first puncture and the time the catheter remained in place.
		65		16.73 ± 0.84 days (8.4-26.81) Weight: 2,570 ± 210 g ^||^ (1,190-3,890)		
Xiao, et al.; 2019 ^(^ ^28^ ^)^ ; China	Experimental Group: USG ^‡‡‡^ for vascular visualization and IC-ECG ^‡‡^ to guide the tip	78	GAB ^*^: 32.17 ± 2.63 w ^†^ (28-37) BW ^§^: 1,520 ± 377.38 g ^||^	15.21 ±7.52 days Weight: 1,657.44 ± 307.22 g ^||^	General complications: 5(6.41%) **x** 14(16.86%); *p* =0.040 Phlebitis: 2(2.56%) **x** 7(10.84%); *p* =0.202 Infection: 1(1.28%) **x** 3(3.61%); *p* =0.657 1 ^st¶^ Malpositioning: 5(6.41%) **x** 22(26.51%); *p* =0.001 Rupture: 2(2.56%) **x** 4(4.82%); *p* =0.735	IC-ECG ^‡‡^ can contribute to reducing the rates of tip repositioning and complications, in addition to increasing the rates of adequate tip positioning on the first attempt.
	Control Group: puncture guided by anatomical landmarks	83	GAB ^*^: 32.36 ± 2.63 w ^†^ (28-37) BW ^§^: 1,508.13 ± 279.31 g ^||^	13.19 ±8.8 days Weight: 1,571.63 ± 266.16 g ^||^		
Zhou et al.; 2017 ^(^ ^37^ ^)^ ; China	Experimental Group: USG ^‡‡‡^ for vascular visualization and IC-ECG ^‡‡^ to guide the tip	49	GAB ^*^: 35 ± 4 w ^†^ (28-41)	17 ± 16 days (1-28) BW: 2,700 ± 900 g ^||^ (1,100-4,900)	1 ^st¶^ Malpositioning: 3(6.12%) **x** 75(37.5%); *p* <0.001 2 ^nd^ ^nd^: Malpositioning (Pleural effusion): 0(0%) **x** 1(0.5%)	IC-ECG ^‡‡^ with saline column can be applied to neonates, guiding the catheter tip appropriately, thus reducing the risks, delays and costs of tip adjustments.
	Control Group: puncture guided by anatomical landmarks	200	GAB ^*^: 36 ± 3 w ^†^ (28-41)	13 ± 12 days (1-28) BW: 2,700 ± 900 g ^||^ (1,000-5,000)		

^‡^ CGA = Corrected Gestational Age;

^**^ USGRT = Real-Time Ultrasound;

^||||^ RR = Relative Risk;

^¶¶^ CI = Confidence Interval;

^***^ L = Size of the PICC to be inserted;

^§§^ PICC = Peripherally Inserted Central Catheter;

^†††^ M = Newborn weight;

^st^ 1 Malpositioning = Primary tip malpositioning;

^§^ BW = Birth Weight;

^‡‡‡^ USG = Ultrasound

^nd^ 2 Malpositionging = Secondary intravascular or extravascular tip malpositioning;

^‡‡^ IC-ECG = Intracavitary Electrocardiogram;

^*^ GAB= Gestational Age at Birth;

^†^ w = Weeks;

^||^ g = Grams;

**Figure 4:**
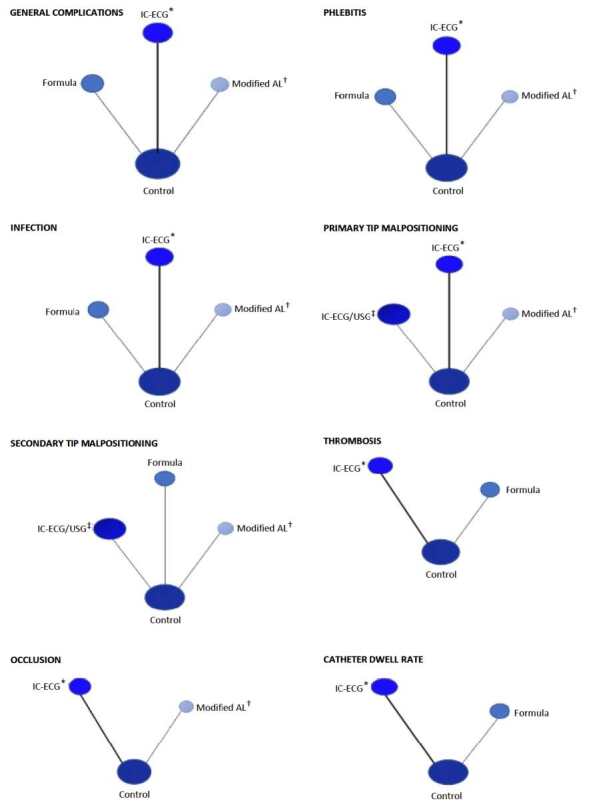
- Network geometry of all outcomes that allowed performing the network meta-analysis. Brasília, DF, Brazil, 2023

 Not all outcomes were assessed in all the studies included. Those evaluated in more than one included study, with a similar sample and more than one insertion technique, were analyzed in the network. In the structure or geometry of each network, the control group was the only one directly compared to the other insertion techniques and these were compared indirectly with each other, precisely for having this common comparator. Thus, for all outcomes in which network meta-analysis was possible, its geometry was the same. The network structure of all outcomes that allowed the network meta-analysis is graphically represented in Figure 4 . It is noted that, in the network geometry circles represent interventions, whose sizes correspond to the number of each sample, and straight lines are direct comparisons between insertion techniques, with greater thickness the more studies that carried out that comparison. 

*IC-ECG = Intracavitary Electrocardiogram; ^†^ AL = Anatomical Landmark; ^‡^ IC-ECG/USG = Intracavitary Electrocardiogram and Ultrasound for vascular visualization

**Figure 5:**
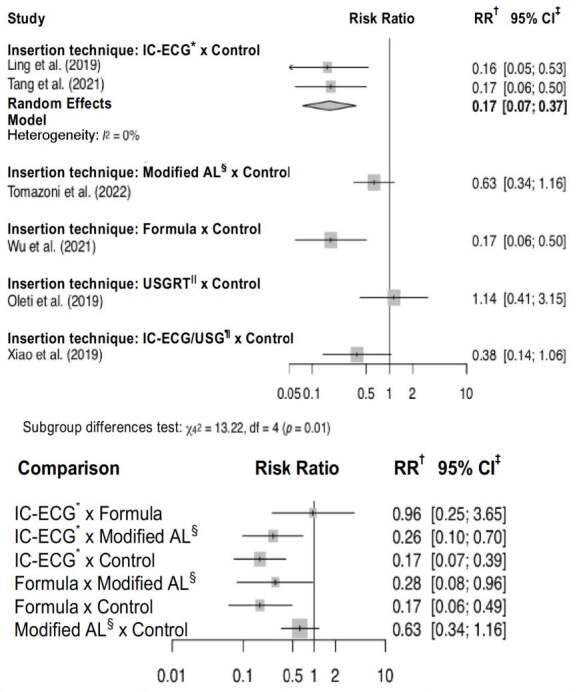
- Forest graphs showing the effect of the insertion techniques on the “general complications” outcome, both from direct comparisons (above) and those carried out on the network (below). Brasília, DF, Brazil, 2023

 In relation to the primary outcomes, one of them was general complications, which corresponded to the total number of complications reported by each study, and may refer to two or more complications, depending on the research objective and the newborn’s follow-up length. Two studies did not verify this outcome ^(^ ^37^ ^-^ ^38^ ^)^ , as they were the only ones that monitored their samples only until the tip location was confirmed and not until the PICC was removed (see device dwell time), as in the others. As was the case with other complications, primary tip malpositioning was not mentioned in the “general complications” outcome by any author of the studies included. Figure 5 shows the results related to the general complications regarding the meta-analysis with direct and indirect comparisons. 

*IC-ECG = Intracavitary Electrocardiogram; ^†^ RR = Relative Risk; ^‡^ CI = Confidence Interval; ^§^ AL = Anatomical Landmark; ^||^ USGRT = Real-Time Ultrasound; ^¶^ IC-ECG/USG = Intracavitary Electrocardiogram and Ultrasound for vascular visualization

 The “phlebitis” complication was analyzed with an intervention meta-analysis and in a network with studies with the same sample. In the intervention with the two studies that used IC-ECG in PICC insertion ^(^ ^14^ ^,^ ^35^ ^)^ , it was found that this technique might significantly reduce the phlebitis risk by up to 81% (RR: 0.19; 95% CI: 0.06-0.65). In the network meta-analysis, when compared to the control, IC-ECG remained the only insertion technique capable of significantly reducing this complication. 

 Primary malpositioning of the PICC tip was an outcome not directly measured in two studies ^(^ ^35^ ^-^ ^36^ ^)^ . All interventions (IC-ECG, IC-ECG/USG, USGRT and modified ARL) reduced the risk of primary tip malpositioning, with an estimated large magnitude, when compared to the control. An intervention meta-analysis was carried out with two studies that compared USGRT to the control group ^(^ ^38^ ^-^ ^39^ ^)^ , with RR of 0.47 (95% CI: 0.31-0.72). However, as these trials ^(^ ^38^ ^-^ ^39^ ^)^ were carried out with very premature and extremely premature newborns, it was not possible to include them in the network meta-analysis carried out with other insertion techniques. In this network, it was also found that IC-ECG/USG reduced the risk of primary malpositioning when compared to modified AL (RR: 0.31; 95% CI: 0.10-0.96). 

**Figure 6:**
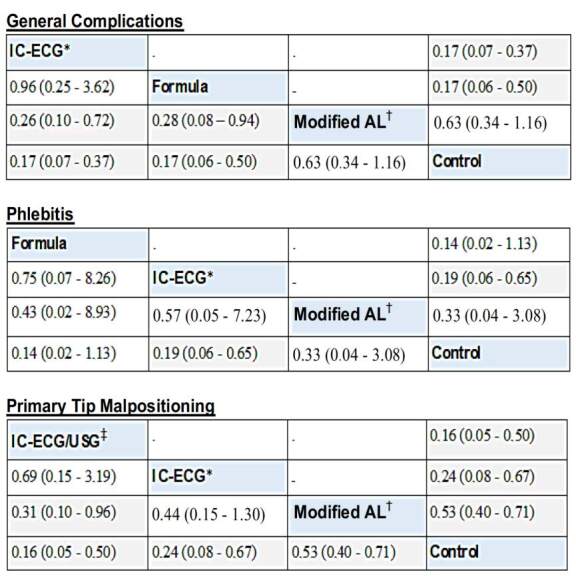
- League tables showing the ranking of the most effective insertion techniques in reducing general complications, phlebitis and primary tip malpositioning with the respective relative risks resulting from the comparisons. Brasília, DF, Brazil, 2023

 Thus, the insertion techniques significantly reduced general complications, phlebitis and primary tip malpositioning. The ranking carried out according to the P-score results of the most effective techniques in reducing those complications is shown in Figure 6 . In this figure, relative risks (RRs) below 1 favor the intervention defined in the column for the network meta-analysis results (lower triangle) and the intervention defined in the row for paired meta-analysis results (upper triangle). Thus, it is verified that the most effective insertion techniques in reducing general complications, phlebitis and primary tip malpositioning were IC-ECG (P-score 0.8399), formula (P-score 0.7562) and IC-ECG/USG (P-score 0.8863), respectively. 

*IC-ECG = Intracavitary Electrocardiogram; ^†^ AL = Anatomical Landmark; ^‡^ IC-ECG/USG = Ultrasound for vascular visualization and tip guided by intracavitary electrocardiogram

 Arrhythmia was investigated through an intervention meta-analysis carried out with the only two studies ^(^ ^14^ ^,^ ^35^ ^)^ that measured this outcome, verifying a reduction in its risk with the use of the IC-ECG insertion technique (RR: 0.09; 95% CI: 0.01-0.71). 

With the “infection”, “secondary tip malpositioning” and “catheter-associated deep vein thrombosis” outcomes, an intervention meta-analysis and a network meta-analysis were performed, with lack of statistical significance in the paired and network effect estimates. A ranking of the most effective insertion techniques in terms of reducing the aforementioned complications was carried out and, in it, the formula was considered the best.

 Two complications mentioned in the 2021 INS manual were not found in any study included in this review: inadvertent arterial puncture; and air embolism ^(^ ^11^ ^)^ . Another two complications were mentioned in the studies included: occlusion; and catheter-associated skin lesion. The meta-analyses carried out for these last two outcomes (network for occlusion and intervention for skin lesions) did not show a significant reduction depending on the insertion technique used. 

 As for the secondary outcomes, the Overall Success Rate (OSR) was above 80% in a study that used USGRT (92.5% x 85%) ^(^ ^39^ ^)^ . An intervention meta-analysis was performed with this outcome with only two homogeneous studies that used USGRT ^(^ ^38^ ^-^ ^39^ ^)^ in their experimental group, resulting in RR: 0.90; 95% CI: 0.60-1.36. 

 The study with the highest success rates in the first puncture was the one that used the formula as insertion technique (96.92% x 72.31%) ^(^ ^36^ ^)^ , whereas the rate reached 37.5% with the modified AL ^(^ ^40^ ^)^ . 

 Regarding the catheter dwell time, the formula ^(^ ^36^ ^)^ and USGRT ^(^ ^39^ ^)^ showed that the experimental group had significantly longer dwell time when compared to the control (MD: 2.35; 95% CI: 2.07-2.63 and MD: 0.9; 95% CI: 0.60-1.10, respectively). The IC-ECG insertion technique reduced the mean dwell time (MD: -0.23; 95% CI: -1.21.-0.75). 

 Certainty of the body of evidence was evaluated through GRADE. No assessed outcome presented “high” certainty of its evidence. Direct and indirect comparisons were evaluated for each outcome, so that a result regarding certainty of the evidence from the network meta-analyses was constructed based on the assessment of the incoherence and imprecision of such networks. Inconsistency in the network, as GRADE calls it, is nonexistent since, for each outcome, there were either direct comparisons (techniques x control) or indirect comparisons (between techniques in the network), but never a combination of direct and indirect comparisons. The evaluation results are shown in Figure 7 . 

**Figure 7:** - Results of the certainty of the evidence assessments from direct/indirect estimates and network meta-analysis, according to the Grading of Recommendations, Assessment, Development, and Evaluation Working Group (GRADE), and carried out on the outcomes (n=11) with their respective comparisons. Brasília, DF, Brazil, 2023

**Outcomes**	**Comparisons**	**Certainty of the evidence from direct estimates**	**Certainty of the evidence from direct estimates**	**Certainty of the evidence from network meta-analysis**
Arrhythmia	IC-ECG ^*^ x Control	Moderate	-	-
General complications	IC-ECG ^*^ x Control Formula x Control Modified AL ^†^ x Control IC-ECG/USG ^‡^ x Control USGRT ^§^ x Control IC-ECG ^*^ x Formula IC-ECG ^*^ x Modified AL ^†^ Formula x Modified AL ^†^	Moderate Moderate Moderate Low Very low - - -	- - - - - Moderate Moderate Moderate	Moderate Low Low - - Low Moderate Low
Phlebitis	IC-ECG ^*^ x Control Modified AL ^†^ x Control Formula x Control Formula x Modified AL ^†^ Formula x IC-ECG ^*^ IC-ECG ^*^ x Modified AL ^†^	Moderate Moderate Moderate - - -	- - - Moderate Moderate Moderate	Moderate Very Low Low Very Low Very Low Very Low
Infection	IC-ECG ^*^ x Control Modified AL ^†^ x Control Formula x Control USGRT ^§^ x Control Formula x Modified AL ^†^ Formula x IC-ECG ^*^ IC-ECG ^*^ x Modified AL ^†^ IC-ECG/USG ^‡^ x Control	Moderate Low Moderate Very low - - - Very low	- - - - Low Moderate Low -	Very low Very low Low - Very low Very low Very low -
Skin lesion	IC-ECG ^*^ x Control	Very low	-	-
Primary malpositioning	IC-ECG ^*^ x Control Modified AL ^†^ x Control IC-ECG/USG ^‡^ x Control USGRT ^§^ x Control IC-ECG/USG ^‡^ x Modified AL ^†^ IC-ECG/USG ^‡^ x IC-ECG ^*^ IC-ECG ^*^ x Modified AL ^†^	Moderate Moderate Very low Moderate - - -	- - - - Low Low Moderate	Moderate Moderate Low - Low Very low Very low
Secondary malpositioning	Modified AL ^†^ x Control Formula x Control IC-ECG/USG ^‡^ x Control USGRT ^§^ x Control Formula x IC-ECG/USG ^‡^ Formula x Modified AL ^†^ Modified AL ^†^ x IC-ECG/USG ^‡^	Moderate Low Very low Very low - - -	- - - - Very low Low Very low	Very low Very low Very low - Very low Very low Very low
Occlusion	IC-ECG ^*^ x Control Modified AL ^†^ x Control IC-ECG ^*^ x Modified AL ^†^	Moderate Moderate -	- - Moderate	Very low Very low Very low
Dwell time	IC-ECG ^*^ x Control Formula x Control USGRT ^§^ x Control IC-ECG ^*^ x Formula	Moderate Moderate Moderate -	- - - Moderate	Very low Moderate - Moderate
Thrombosis	IC-ECG ^*^ x Control Formula x Control Formula x IC-ECG ^*^	High Moderate -	- - Moderate	Low Very low Very low
Overall Success Rate	Modified AL ^†^ x Control USGRT ^§^ x Control	Low Very low	- -	- -

^‡^ IC-ECG/USG = Intracavitary Electrocardiogram with Ultrasound for visualization;

^*^ IC-ECG = Intracavitary Electrocardiogram;

^†^ AL = Anatomical Landmark;

^§^ USGRT = Real-Time Ultrasound

## Discussion

This review is the first to verify the effectiveness of different PICC insertion techniques in preventing the occurrence of complications in newborns. With the synthesis, evaluation and careful combination of the eight studies included through paired and network meta-analyses, it was possible to verify which insertion technique, among those identified, is most effective in reducing certain complications associated with PICC central vascular access devices. In this way, there was a significant reduction in the risk of general complications, arrhythmias, phlebitis and primary malpositioning of the PICC tip, as well as changes in the catheter dwell time depending on the insertion technique used. Infection, infiltration/leaks, secondary tip malpositioning, catheter rupture, thrombosis, occlusion, skin injury and OSR were influenced by the insertion techniques and the majority with an absolute reduction, but without statistical significance and presenting effect estimates with very low certainty of the evidence.

 Preventing complications can reduce morbidity, the need for additional procedures and costs ^(^ ^41^ ^)^ . Good practices during PICC insertion in newborns can mitigate unnecessary exposure of the infants, multiple venipunctures and risk of inappropriate catheter positioning, in addition to improving the insertion success rate ^(^ ^42^ ^-^ ^43^ ^)^ . 

In this review, the use of IC-ECG was associated with a significantly lower and more effective risk of general complications phlebitis and primary tip malpositioning and with a reduction in cardiac arrhythmias, all with moderate certainty of the evidence. It was also the best technique to reduce the catheter dwell time, especially when compared to the technique that uses the formula. With such moderate certainty of the findings, it can be stated that IC-ECG is an insertion technique that should be used in the clinical practice due to its effectiveness in preventing important complications.

 A Chinese systematic review and meta-analysis carried out with local randomized clinical trials found that IC-ECG reduced the risk of phlebitis (OR: 0.33 - 95% CI: 0.19-0.56, *p* <0.001) and the total complications (OR: 0.23 - 95% CI: 0.16-0.33, *p* <0.001), in addition to increasing the accuracy of optimal tip location (OR: 5.37 - 95% CI: 3.80-7.59, *p* <0.001) ^(^ ^44^ ^)^ . This meta-analysis corroborates the results herein presented, showing the effectiveness of IC-ECG in reducing arrhythmias, general complications and phlebitis, in addition to primary tip malpositioning and its possible consequences. This is because research studies and sets of good practices have associated increased complication rates with non-central PICC tips, especially occlusions, leaks, other mechanical complications, phlebitis and non-elective failure/removal ^(^ ^2^ ^,^ ^11^ ^,^ ^29^ ^,^ ^41^ ^,^ ^45^ ^-^ ^46^ ^)^ . The complication rate for non-central PICCs is up to 200% higher when compared to central ones ^(^ ^29^ ^)^ . In other words, an insertion technique capable of preventing primary malpositioning of the central vascular access device will reduce the occurrence of these other complications mentioned above, which can be extremely harmful. 

 Phlebitis is influenced by the PICC insertion technique, as it can be caused when the tip is not central ^(^ ^2^ ^,^ ^35^ ^,^ ^47^ ^)^ (smaller caliber vessels are more subjected to chemical irritation and constant friction from the catheter). It can also be associated with other factors such as a very rigid catheter, inadequate puncture technique, inappropriate puncture site due to constant movement of the limb (especially upper limbs), infection and medication incompatibility ^(^ ^11^ ^,^ ^35^ ^,^ ^48^ ^-^ ^49^ ^)^ . Therefore, as the IC-ECG insertion technique most effectively reduced tip malpositioning, it is no surprise that it is also the best in reducing the risk of phlebitis. 

 Regarding the dwell time, it can be stated that IC-ECG was not effective in reducing it when compared to the control, although it reduced it significantly when compared to the formula. This result is different from the one found in the Chinese systematic review and meta-analysis, in which using IC-ECG during insertion reduced this outcome when compared to the control (MD=5.86, 95% CI: 16.36-4.65, *p* =0.00) ^(^ ^44^ ^)^ . It is noted that the dwell time during which the catheter is in place should only be discussed if other data that were not collected and were not in the studies included in this review are added. This is because interpretation of the decrease in DT can either be considered positive, if this was due to a reduction in complications and consequent completion of intravenous therapy more quickly, for example, or as negative, if it was due to more early catheter removals. 

 The insertion technique that uses a formula based on the newborn’s weight to predict the size (in centimeters) of the catheter to be inserted ( Figure 3 ) proved to be superior to the others by significantly increasing the dwell time (moderate certainty of the evidence) and reducing the risk of phlebitis (low confidence in the effect). This low certainty of the evidence in relation to phlebitis leads to the consideration that the most effective technique in reducing its risk was in fact IC-ECG, in which confidence is moderate, with more precise estimates. Furthermore, it was the second best technique in reducing the risk of general complications and the one with the best success rate at the first puncture attempt, less need for PICC adjustments, hospitalization time and pain ^(^ ^36^ ^)^ . 

 Other research studies with newborns also resulted in formulas that predict the size of the catheter to be inserted, based on the infants’ physical characteristics such as weight, height and gestational age, ensuring a more accurate way of centrally locating the PICC tip ^(^ ^25^ ^,^ ^50^ ^)^ . 

 In the study using the formula, the saphenous vein was punctured, almost always on the first attempt and by trained and experienced nurses ^(^ ^36^ ^)^ . The experience of a vascular nursing team is important for successful insertion and safety in terms of complication rates ^(^ ^28^ ^,^ ^51^ ^)^ . 

 It is noted that, in addition to the warm compress used in research with the formula ^(^ ^36^ ^)^ , other studies tested different strategies to keep the NBs warm and veins vasodilated during PICC insertion, such as using of a thermal blanket ^(^ ^52^ ^)^ and placing the NB in a crib with radiant heat during insertion of this device ^(^ ^4^ ^)^ . These tactics and the results that were found as for the number of insertions on the first puncture in the study that used the warm compress (96.92%, n=63) ^(^ ^36^ ^)^ and a heated crib (77.72%, n=457) ^(^ ^4^ ^)^ show the need to actually employ measures to prevent newborn hypothermia during prolonged procedures such as PICC insertion. 

 IC-ECG associated with USG for vascular visualization was considered the best insertion technique in reducing primary tip malpositioning according to the network meta-analysis ranking. However, it should be considered that the studies that used it were non-randomized, with higher than moderate risk of bias and very low and low certainty of the evidence. Therefore, it is correct to say that the most effective technique in reducing the risk of primary malpositioning was IC-ECG alone and that IC-ECG/USG was the second best, surpassing modified AL and control. The other outcomes measured in the studies that used IC-ECG/USG ^(^ ^28^ ^,^ ^37^ ^)^ also resulted in estimates with a significant uncertainty degree and without statistical significance. This shows that, with regard to the prevention of PICC-associated complications, IC-ECG/USG is either not advantageous in the clinical practice for this purpose or requires randomized studies to be carried out to more reliably verify the existence of benefits or not. 

 However, employing devices to assist with visualization and venipuncture can be useful, as newborns have narrower veins that are immature and vulnerable to ruptures ^(^ ^1^ ^,^ ^25^ ^-^ ^26^ ^)^ . In the research with modified AL there was also failure in the puncture of 18 newborns for PICC insertion due to venous fragility ^(^ ^40^ ^)^ . 

 The INS recommends using USG to evaluate the vein that will be punctured regarding its caliber (the catheter cannot exceed 45% of the vessel), whether there are abnormalities such as occlusions and thrombosis and to identify positioning of the device tip ^(^ ^11^ ^)^ . In a descriptive survey carried out by nurses from Iran with 30 newborns, a USG device was used for vascular assessment and visualization during the PICC insertion puncture. There was an increase in the success rate at the first (68% USG group x 60% conventional group) and second (50% x 40%) attempts, although not statistically significant ^(^ ^53^ ^)^ . An American retrospective study whose objective was to describe the use of USG in PICC insertion found a success rate of 100% in a sample comprised by 10 newborns with multiple unsuccessful vascular access attempts ^(^ ^54^ ^)^ . 

 The insertion technique that used modified AL ^(^ ^40^ ^)^ reduced the risk of general complications and primary malpositioning with statistical significance (low and moderate to very low certainty of the evidence, respectively). 

 A retrospective study carried out with 588 NBs found low prevalence of complications associated with the catheter (10.71%) and also carried out measurements up to the right sternoclavicular junction, such as the study with modified AL ^(^ ^40^ ^)^ , stating that the traditional measurement ended up positioning the PICC tip too deep ^(^ ^4^ ^)^ . Therefore, when it comes to NBs, it is imperative to carry out a prior measurement of the catheter length to be introduced based on anatomical landmarks, differently from what is recommended by the INS for other populations in the upper limbs (puncture site: right sternoclavicular region and third intercostal space) ^(^ ^11^ ^)^ . 

 USGRT proved to be effective in reducing primary tip malpositioning and increasing catheter dwell time significantly and with low and moderate certainty in the findings, respectively. It did not present significant effect estimates for the other complications and even obtained opposite estimates, with no significance and very low certainty in some cases, such as general complications, infection and secondary tip malpositioning. It must be considered that the studies which used this insertion technique ^(^ ^38^ ^-^ ^39^ ^)^ were carried out with very premature and extremely premature newborns, whose peculiarities may have led to a tendency for an increase in some complications or to lack of a significant reduction in others, as was the case with the other techniques. Furthermore, they were the studies with the smallest sample size. 

 A prospective cohort monitored PICC insertion in the lower limbs of 166 newborns in their experimental group with USGRT and compared it to 141 newborns in whom this technology was not used. As in this review, there was a significant reduction in primary tip malpositioning, as the need for catheter adjustments in the USGRT group was much lower (10.84% x 65.95%, *p* <0.001) and there was no significant difference between the groups in other complications (phlebitis, occlusion, bacteremia and rupture) ^(^ ^12^ ^)^ . 

 A recent meta-analysis also validates the results found in this review by verifying that USG is excellent for locating the PICC tip in a neonatal unit when compared to x-ray, with 95.2% sensitivity and 71.4% specificity, and that it should be combined with x-ray when it is not possible to position the tip using this method ^(^ ^55^ ^)^ . 

 With the negative results of the studies that used USGRT, it is clear that it is imperative to establish protocols ^(^ ^56^ ^-^ ^57^ ^)^ , as well as adequate training and experience of professionals who manipulate the equipment ^(^ ^57^ ^)^ . When it comes to newborns and children, the need for these requirements was highlighted as a disadvantage of USG, as well as the difficulties of some units having a device available 24 hours a day ^(^ ^57^ ^-^ ^59^ ^)^ . 

 Several changes have been made to the materials and procedures related to PICC insertion and maintenance in newborns and the use of technologies to assist punctures and, in this sense, central positioning of the PICC has been most recommended in the current literature ^(^ ^11^ ^,^ ^56^ ^,^ ^60^ ^-^ ^61^ ^)^ . These technologies aim at reducing complication rates and, consequently, newborns’ pain, suffering and morbidity and mortality ^(^ ^60^ ^)^ . However, none of the recently published Brazilian studies ^(^ ^26^ ^,^ ^42^ ^,^ ^62^ ^)^ have observed the use of such technologies (IC-ECG, USGRT, USG for vascular visualization, etc.), which leads us to reflect on what reasons have contributed to the fact that they are not being used and studied in Brazil. 

 This systematic review and meta-analysis (paired and in network) verified the effectiveness of insertion techniques in preventing the occurrence of PICC-related complications in newborns, which can even lead to sequelae and death. It included nearly six insertion techniques, more than 1,000 NBs in its sample, and used with methodological rigor and differentiated analyses. The studies included were all randomized and non-randomized controlled clinical trials, evaluated together as already recommended in the literature ^(^ ^63^ ^)^ , the majority of which had a sample size greater than 100 newborns. This fact can be considered an advantage, as clinical trials related to intravenous catheters in newborns generally have a mean sample size of less than 100 participants ^(^ ^64^ ^)^ . 

However, this review should be interpreted in the context of some limitations. A small sample of comparative studies was evaluated, with the possibility of carrying out few intervention meta-analyses. In addition to that, no study compared different technologies to each other for PICC insertion, the risk of bias of most of the studies included was judged as “Some concerns”, and no outcome was assessed with high certainty of the evidence.

## Conclusion

This review showed that the insertion techniques are capable of preventing PICC-related complications in newborns; however, it is not yet possible to assert which is the most effective for all. This is because each technique included in this review showed greater effectiveness for different complications, even though IC-ECG reduced most of them.

All techniques other than anatomical landmark-guided puncture (control) reduced primary tip malpositioning, but IC-ECG was the most effective in reducing it along with general complications, phlebitis and catheter dwell time, also considerably and significantly reducing arrhythmias. The formula showed greater effectiveness in increasing catheter dwell time and was the second best in reducing general complications. It was also considered superior with very low certainty of the evidence and no statistical significance in reducing the risks of infection, secondary tip malpositioning and thrombosis. USGRT did not show any reduction in other complications, it increased catheter dwell time and found a tendency for some to increase without significance.

There is a need for more randomized controlled clinical trials with larger samples that compare insertion techniques in terms of complication rates. Cost-effectiveness studies should also be operationalized so that public policies may be developed to reduce failures in intravenous therapy for patients of all ages and provide greater safety. Every effort must be directed towards investigating more strategies, techniques and technologies so that PICC use, essential for newborns, becomes safer for this vulnerable population.
