# Supplementary material for: Prevention of complications related to peripherally inserted central catheter insertion techniques in newborns: systematic review and network meta-analysis
Source: Rev Lat Am Enfermagem. 2024 Jul 5;32:e4161. doi: 10.1590/1518-8345.6905.4161 (PMC11251685; doi:10.1590/1518-8345.6905.4161)
Supplement: Translation

# Translation: Prevención de complicaciones asociadas con las técnicas de inserción de catéter central de inserción periférica en recién nacidos: revisión sistemática y metaanálisis en red ^*^

**DOI:** 10.1590/1518-8345.6905.4160

**Authors:** Ludmylla de Oliveira Beleza, Guilherme da Costa Brasil, Amanda Salles Margatho, Christiane Inocêncio Vasques, Renata Cristina de Campos Pereira Silveira, Priscilla Roberta Silva Rocha, Laiane Medeiros Ribeiro

**Affiliations:** ^1^ Universidade de Brasília, Brasília, DF, Brasil.; ^2^ Secretaria do Estado de Saúde, Hospital Materno Infantil de Brasília, UTI Neonatal, Brasília, DF, Brasil.; ^3^ Centro Universitário do Distrito Federal, Brasília, DF, Brasil.; ^4^ Universidade de São Paulo, Escola de Enfermagem de Ribeirão Preto, PAHO/WHO Collaborating Centre for Nursing Research Development, Ribeirão Preto, SP, Brasil.; ^5^ Universidade de Brasília, Faculdade de Ciências da Saúde, Brasília, DF, Brasil.; ^6^ Universidade de Brasília, Faculdade de Ceilândia, Brasília, DF, Brasil.

**Keywords:** Recíen Nacido, Cateterismo Periférico, Catéteres Venosos Centrales, Insuficiencia del Tratamiento, Enfermería Neonatal, Unidades de Cuidado Intensivo Neonatal

## Abstract

**Objetivo:**

analizar la efectividad de las técnicas de inserción de catéter central de inserción periférica en la prevención de la aparición de complicaciones asociadas con este dispositivo en recién nacidos.

**Método:**

revisión sistemática de la literatura y metaanálisis pareado y en red, la búsqueda se realizó en siete bases de datos y en la literatura gris, se incluyeron ensayos clínicos aleatorizados y no aleatorizados. El riesgo de sesgo se evaluó mediante las herramientas *Cochrane Risk of Bias 2* y *Risk of Bias In Non-randomized Studies of Interventions* . La certeza de la evidencia a través de la *Grading of Recommendations Assessment, Development and Evaluation* . Se realizó un metaanálisis con ayuda del programa estadístico R.

**Resultados:**

se incluyeron ocho estudios, con 1126 recién nacidos, y se identificaron seis técnicas de inserción: electrocardiograma intracavitario, electrocardiograma intracavitario asociado a ultrasonido, ultrasonido, fórmula, punto anatómico de referencia y punto anatómico de referencia modificado. Cinco técnicas redujeron significativamente el mal posicionamiento primario de la punta en comparación con el control (p<0,05). El electrocardiograma intracavitario redujo de manera significativa y más efectiva las arritmias, las complicaciones generales y la flebitis; la técnica que utilizó una fórmula también redujo las complicaciones generales. La infección, la infiltración, el mal posicionamiento secundario de la punta, la rotura del catéter, la trombosis, la oclusión y las lesiones de la piel asociadas con el catéter son eventos que no se revinieron significativamente.

**Conclusión:**

el electrocardiograma intracavitario y el uso de la fórmula fueron las técnicas más efectivas para reducir las complicaciones.

## Destacados

**(1)** Algunas técnicas de inserción de PICC han reducido las complicaciones asociadas.

**(2)** El mal posicionamiento primario de la punta fue la complicación que más se previno.

**(3)** El electrocardiograma intracavitario redujo arritmias, flebitis y complicaciones generales.

**(4)** La técnica que utilizó la fórmula redujo significativamente las complicaciones generales.

## Introducción

 El catéter central de inserción periférica (PICC), denominado *Peripherally Inserted Central Catheter* (PICC) en inglés, es ampliamente utilizado en recién nacidos (RN) ^(^ ^1^ ^-^ ^2^ ^)^ . Presenta varias ventajas con respecto al uso de otros dispositivos de acceso venoso central y de catéteres cortos de inserción periférica, como preservación del sistema vascular periférico ^(^ ^3^ ^)^ , menor riesgo de flebitis e infecciones ^(^ ^3^ ^-^ ^4^ ^)^ , menos necesidad de someter al recién nacido a repetidas punciones ^(^ ^4^ ^-^ ^5^ ^)^ , dolor y estímulos ^(^ ^3^ ^-^ ^5^ ^)^ . 

 A pesar de las ventajas, el uso del PICC se asocia con diferentes tipos de complicaciones que pueden aumentar la morbimortalidad neonatal ^(^ ^6^ ^-^ ^7^ ^)^ , incrementar los costos para el sistema de salud y comprometer la calidad de vida de los pacientes ^(^ ^8^ ^-^ ^10^ ^)^ . 

 Las complicaciones asociadas con el PICC se pueden presentar en su inserción, durante todo el período de permanencia e incluso después de su remoción ^(^ ^6^ ^)^ . Según la *Infusion Nursing Society* (INS) (2021), las complicaciones asociadas con la inserción de dispositivos de acceso venoso central como el PICC son: punción arterial, arritmias cardíacas, lesión de nervios, embolia aérea y mal posicionamiento primario del dispositivo de acceso venoso central ^(^ ^11^ ^)^ . Se podrían prevenir muchas complicaciones, como la trombosis venosa profunda asociada con el catéter, mal posicionamiento secundario (migración de la punta del catéter intra o extravascular), infección del torrente sanguíneo asociada con la vía central, flebitis, infiltración, extravasación, ruptura del catéter, oclusión y lesiones de la piel asociadas con el dispositivo, dependiendo de la técnica de inserción utilizada ^(^ ^11^ ^)^ . 

 Desde el punto de vista de la técnica de inserción, algunas precauciones, como el método utilizado para guiar la inserción, determinan las complicaciones asociadas con el PICC, es decir, las acciones realizadas durante la inserción de este dispositivo pueden prevenir complicaciones asociadas con su introducción y las complicaciones tardías que se presentan con la permanencia del PICC ^(^ ^11^ ^)^ . 

 Los estudios y el conjunto de buenas prácticas recomiendan el uso de tecnologías en la inserción del PICC para prevenir las complicaciones asociadas y aumentar la seguridad del paciente, como ultrasonido (US) ^(^ ^11^ ^-^ ^12^ ^)^ , relación catéter-vena ^(^ ^11^ ^)^ y electrocardiograma intracavitario (IC-ECG) ^(^ ^11^ ^,^ ^13^ ^-^ ^14^ ^)^ . Para las instituciones de salud, el tratamiento de las complicaciones tiene una reducción de costos asociada, dado que, en ciertas situaciones, la remoción de este dispositivo requiere otra punción y, muchas veces, es necesario usar un nuevo catéter, además aumenta el tiempo que le dedican los enfermeros ^(^ ^5^ ^,^ ^10^ ^-^ ^11^ ^)^ . 

 El US se puede utilizar para determinar el tamaño ideal del catéter que se va a introducir según el diámetro de la vena (relación catéter-vena), para ubicar la punta y visualizar la vena para guiar la punción. Esto puede reducir las complicaciones y la necesidad de reposicionamiento, además de aumentar las posibilidades de éxito en el primer intento de punción y proporcionarle una mayor satisfacción al paciente ^(^ ^11^ ^-^ ^12^ ^,^ ^15^ ^)^ . 

 El IC-ECG, mediante electrodos colocados en la superficie corporal y en contacto con el catéter, monitorea los cambios en el tamaño de la onda P en el electrocardiograma, cuya elevación indica que la punta del catéter ha llegado a la unión cavo-auricular a través de la vena cava superior ^(^ ^14^ ^)^ . Esta técnica muestra, en tiempo real, la posición correcta de la punta del dispositivo, lo que lleva a un posicionamiento central significativamente más exitoso debido a su ajuste inmediato, además de brindar tratamiento inmediato y eliminar la necesidad de confirmar la posición de la punta mediante examen radiológico ^(^ ^13^ ^-^ ^14^ ^,^ ^16^ ^)^ . 

No se identificaron estudios de revisión sobre la relación entre todas las técnicas de inserción y las complicaciones asociadas con el PICC en recién nacidos en la literatura ni en las plataformas de registro de esta revisión.

 Por ese motivo, este estudio tuvo como objetivo analizar la efectividad de las técnicas de inserción de catéter central de inserción periférica en la prevención de la aparición de complicaciones asociadas con este dispositivo en los recién nacidos. Para lograr este objetivo se utilizó un metaanálisis en red, ya que permite comparar tres o más intervenciones distintas simultáneamente en un solo análisis, y combinar evidencia directa (estudios en los que se comparan las intervenciones de interés) e indirecta (estudios en los que se comparan las intervenciones de interés con un comparador común). Las estimaciones de los efectos de los estudios de comparación directa e indirecta se combinan en una red de intervenciones ^(^ ^17^ ^)^ . 

## Método

 Esta es una revisión sistemática de la literatura con metaanálisis pareado y en red, que sigue las pautas de PRISMA ( *Preferred Reporting Items Systematic Reviews and Meta-Analysis Checklist* ) ^(^ ^18^ ^)^ y PRISMA- *Network Meta-Analysis* (PRISMA-NMA) ^(^ ^19^ ^)^ . El protocolo se registró en la plataforma PROSPERO, bajo el número CRD42022324152. 

 Se utilizó la estrategia PICOS ^(^ ^17^ ^)^ (acrónimo de *Population, Interventions, Comparators, Outcomes, Study design* ) para definir la pregunta orientadora de esta revisión: en pacientes recién nacidos (P), ¿qué técnica de inserción de PICC (I) es más efectiva para prevenir la aparición de complicaciones asociadas con el uso de este dispositivo (O)? 

### Criterio de elegibilidad

Se incluyeron ensayos clínicos controlados aleatorizados y no aleatorizados que evaluaron las técnicas de inserción del PICC en recién nacidos hospitalizados y las complicaciones asociadas con el PICC informadas como medidas de resultado.

Se excluyeron los estudios que evaluaron complicaciones asociadas con el uso de PICC en recién nacidos que recibían atención ambulatoria; quienes evaluaron las complicaciones asociadas con el uso de PICC en recién nacidos en Unidades de Cuidados Intensivos Mixtas (que atienden a recién nacidos junto con niños); que evaluaron las complicaciones asociadas con el uso de PICC en niños y adultos; que no describieron las técnicas de inserción; que evaluaron diferentes tipos de catéteres PICC, sitios de inserción o apósitos; que evaluaron técnicas de inserción de otros tipos de dispositivos de acceso venoso central; estudios observacionales; estudios cualitativos; resúmenes de congresos, revisiones, editoriales, opiniones de especialistas, informes y series de casos, artículos de reflexión; y estudios sin grupo de comparación.

### Fuentes de información

 La búsqueda se realizó en las siguientes bases de datos electrónicas: *Cumulative Index to Nursing and Allied Health Literature* (CINAHL), Cochrane CENTRAL, Embase, Literatura Latinoamericana y del Caribe en Ciencias de la Salud (LILACS), PubMed, Scopus y *Web of Science* . La literatura gris fue consultada a través de las bases de datos *Google Académico* y *Open Gray* . También se consultó la lista de referencias de los estudios incluidos para identificar publicaciones no identificadas previamente en las búsquedas. Además, se realizó una búsqueda activa de publicaciones que pudieran conformar la muestra para esta revisión a través de consultas con especialistas ese día. La búsqueda se realizó el 1 de enero de 2023. 

### Estrategia de búsqueda

 La estrategia de búsqueda se desarrolló a partir de la pregunta orientadora de esta revisión, utilizando el operador booleano “OR” entre sinónimos y similares y “AND” entre palabras clave. Inicialmente, la estrategia se estructuró para la base de datos PubMed, utilizando los descriptores *Medical Subject Headings* (MeSH) y palabras clave, y luego se adaptó para cada una de las bases de datos consultadas. No hubo restricciones de tiempo ni de idioma para las publicaciones. 

La estrategia de búsqueda desarrollada en la base de datos PubMed fue la siguiente: (“infant, newborn”[MeSH Terms] OR “Newborn Infant”[All Fields] OR “Newborn Infants”[All Fields] OR “Newborns”[All Fields] OR “Newborn”[All Fields] OR “Neonate”[All Fields] OR “Neonates”[All Fields] OR “infant, premature”[MeSH Terms] OR “Premature Infant”[All Fields] OR “Preterm Infants”[All Fields] OR “Preterm Infant”[All Fields] OR “Premature Infants”[All Fields] OR “Neonatal Prematurity”[All Fields] OR “Preterm”[All Fields] OR “infant, extremely premature”[MeSH Terms] OR “Extremely Premature”[All Fields] OR “Extremely Premature Infant”[All Fields] OR “Extremely Preterm Infants”[All Fields] OR “Extremely Preterm Infant”[All Fields] OR “Extremely Premature Infants”[All Fields] OR “infant, extremely low birth weight”[MeSH Terms] OR “Extremely Low Birth Weight Infant”[All Fields] OR “Extremely Low Birth Weight Infants”[All Fields] OR “infant, very low birth weight”[MeSH Terms] OR “very low birth weight infant”[All Fields] OR “very low birth weight infants”[All Fields] OR “very low birth weight infant”[All Fields] OR “very low birth weight infants”[All Fields] OR “Very Low Birth Weight”[All Fields] OR “infant, low birth weight”[MeSH Terms] OR “low birth weight infant”[All Fields] OR “low birth weight infants”[All Fields] OR “low birth weight infant”[All Fields] OR “low birth weight infants”[All Fields] OR “Low Birth Weight”[All Fields] OR “Low Birth Weights”[All Fields] OR “infant, small for gestational age”[MeSH Terms] OR “Small for Gestational Age”[All Fields] OR “Term Birth”[MeSH Terms] OR “Term Births”[All Fields] OR “Fullterm Birth”[All Fields] OR “Fullterm Births”[All Fields] OR “infant, postmature”[MeSH Terms] OR “Postmature Infant”[All Fields] OR “Postmature Infants”[All Fields] OR “Neonatal”[All Fields] OR “intensive care units, neonatal”[MeSH Terms] OR “Neonatal Intensive Care Unit”[All Fields] OR “Neonatal Intensive Care Units”[All Fields] OR “Neonatal Intensive Care”[All Fields] OR “Neonatal ICU”[All Fields] OR “Neonatal ICUs”[All Fields]) AND (“catheterization, peripheral”[MeSH Terms] OR “Peripherally Inserted Central Catheter”[All Fields] OR “Peripherally Inserted Central Catheters”[All Fields] OR “PICC”[All Fields] OR “PICCs”[All Fields] OR “Peripherally Inserted Central Catheter Line”[All Fields] OR “Peripherally Inserted Central Catheter Lines”[All Fields] OR “PICC Line”[All Fields] OR “PICC Lines”[All Fields]) AND (“Complications”[MeSH Subheading] OR “Complication”[All Fields] OR “Treatment Failure”[MeSH Terms] OR “Treatment Failures”[All Fields] OR “PICC Failure”[All Fields] OR “PICC Complication”[All Fields] OR “PICC Complications”[All Fields] OR “Infections”[All Fields] OR “Infection”[All Fields] OR “Catheter-Related Infections”[MeSH Terms] OR “catheter related infection”[All Fields] OR “catheter related infection”[All Fields] OR “Central Line-Associated Bloodstream Infection”[All Fields] OR “CLABSI”[All Fields] OR “Central Line-Associated Bloodstream Infections”[All Fields] OR “Bloodstream Infection”[All Fields] OR “Bloodstream Infections”[All Fields] OR “Catheter-Associated Bloodstream”[All Fields] OR “Catheter-Associated Bloodstream Infections”[All Fields] OR “Central Line Bloodstream Infection”[All Fields] OR “CABSI”[All Fields] OR “CRBSI”[All Fields] OR “Neonatal Nosocomial Infections”[All Fields] OR “Neonatal Nosocomial Infection”[All Fields] OR “Deep Vein Thrombosis”[All Fields] OR “Catheter-Related Deep Vein Thrombosis”[All Fields] OR “Thrombosis”[MeSH Terms] OR “Thromboses”[All Fields] OR “Thrombus”[All Fields] OR “Blood Clot”[All Fields] OR “Blood Clots”[All Fields] OR “Phlebitis”[MeSH Terms:noexp] OR “Phlebitides”[All Fields] OR “Periphlebitis”[All Fields] OR “Adverse Effects”[MeSH Subheading] OR “Adverse Effect”[All Fields] OR “Pericardial effusion”[All Fields] OR “Cardiac Tamponade”[All Fields] OR “Arterial Puncture”[All Fields] OR “Nerve Injury”[All Fields] OR “Nerve Injuries”[All Fields] OR “embolism, air”[MeSH Terms] OR “Air Embolism”[All Fields] OR “Air Embolisms”[All Fields] OR “arrhythmias, cardiac”[MeSH Terms] OR “Cardiac Dysrhythmia”[All Fields] OR “Cardiac Dysrhythmias”[All Fields] OR “Arrhythmia”[All Fields] OR “Arrythmia”[All Fields] OR “Cardiac Dysrhythmia”[All Fields] OR ((“central”[All Fields] OR “centrally”[All Fields] OR “centrals”[All Fields]) AND (“vascular access devices”[MeSH Terms] OR (“vascular”[All Fields] AND “access”[All Fields] AND “devices”[All Fields]) OR “vascular access devices”[All Fields] OR (“vascular”[All Fields] AND “access”[All Fields] AND “device”[All Fields]) OR “vascular access device”[All Fields]) AND (“malposition”[All Fields] OR “malpositioned”[All Fields] OR “malpositioning”[All Fields] OR “malpositionings”[All Fields] OR “malpositions”[All Fields])) OR “Infiltration”[All Fields] OR “Extravasation”[All Fields] OR “Extravasation of Diagnostic and Therapeutic Materials”[MeSH Terms]).

### Proceso de selección

 Después de eliminar los duplicados utilizando el *software Endnote Web* ( *Clarivate Analytics* ) ^(^ ^20^ ^)^ , se exportó la lista de referencias de los estudios identificados en las bases de datos al *software* *online* *Rayyan* ( *Rayyan* , *Qatar Computing Research Institute* ) ^(^ ^21^ ^)^ . 

El proceso de selección de los estudios se llevó a cabo en dos fases. En la primera, dos revisores (L.O.B.; G.B.), de forma independiente, leyeron los títulos y resúmenes de los estudios identificados en las bases de datos, aplicando los criterios de elegibilidad. En segunda fase, los revisores (L.O.B.; G.B.) aplicaron nuevamente los criterios de inclusión después de leer los estudios completos. Los desacuerdos se resolvieron mediante discusión entre ambas partes y solo se contactó al tercer revisor (A.S.M.) cuando no se llegó a un consenso.

### Proceso de recolección de datos

Los dos revisores (L.O.B.; G.B.) extrajeron los siguientes datos de los estudios seleccionados en la segunda fase: características del estudio (autor, año de publicación, país, objetivos del estudio y conclusiones principales) y de la muestra (número de RN y catéteres; edad gestacional y peso al nacer, edad gestacional corregida o días de vida y peso al momento de la inserción del PICC), técnica de inserción realizada y comparador, desenlaces primarios y secundarios.

 Los datos recolectados se organizaron en tablas en *Microsoft Word* ^®^ y un revisor ingresó los desenlaces en el programa *Microsoft Excel* ^®^ , otro revisor realizó una verificación cruzada independiente. Posteriormente los datos fueron exportados al *software* R versión 4.2.3. 

### Desenlaces

 Las tasas de complicaciones generales y específicas relacionadas con el PICC se consideraron desenlaces primarios. La definición de complicaciones se basó en los términos descritos en el conjunto de buenas prácticas de la INS (2021), al igual que las complicaciones asociadas con la técnica de inserción: punción arterial, arritmias cardíacas, lesión de nervios, embolia aérea y mal posicionamiento primario del dispositivo de acceso venoso central. Las complicaciones asociadas con el uso del catéter fueron trombosis venosa profunda asociada con el catéter, mal posicionamiento secundario (migración de la punta del catéter intra o extravascular), flebitis, infiltración y extravasación, infección del torrente sanguíneo asociada con la vía central, oclusión, ruptura del catéter y lesiones de la piel asociadas con el catéter ^(^ ^11^ ^)^ . Estas últimas complicaciones que ocurren durante la permanencia del PICC se incluyeron porque se consideran prevenibles, dependiendo de la técnica de inserción utilizada ^(^ ^11^ ^)^ . Además, como sólo se incluyeron estudios que cambiaron únicamente la técnica de inserción entre los grupos estudiados, o sea, no hubo diferencias en el material utilizado ni en los procedimientos de cuidado y mantenimiento del PICC, se puede decir que estos desenlaces también analizados por los autores, en estos estudios, sólo estarían relacionados con las técnicas de inserción utilizadas. Las diferentes nomenclaturas utilizadas por los autores del estudio para definir las complicaciones fueron estandarizadas según la descripción propuesta en el conjunto de buenas prácticas de la INS ^(^ ^11^ ^)^ . 

Los desenlaces secundarios obtenidos de los estudios que tenían estos datos fueron: tasa de éxito general (TEG) o número de PICC insertados exitosamente en todos los intentos de inserción; tasa de éxito de la primera punción; número de punciones; y tiempo de permanencia del catéter.

### Riesgo de sesgo de los estudios incluidos

 La evaluación del riesgo de sesgo de los ensayos clínicos aleatorizados incluidos fue realizada por dos revisores, utilizando la herramienta *Risk of Bias 2* (RoB2) ^(^ ^17^ ^)^ , desarrollada por la Colaboración Cochrane. Los ensayos clínicos no aleatorizados se evaluaron utilizando la herramienta *Risk of Bias In Non-Randomized Studies of Interventions* (ROBINS-I), también desarrollada por la Colaboración Cochrane ^(^ ^22^ ^)^ . 

### Medidas del efecto

 Los datos extraídos y los resultados obtenidos se expresaron mediante frecuencias relativas y absolutas o mediante medias y desviaciones estándar. Para los desenlaces binarios, la medida del efecto utilizada fue el riesgo relativo (RR) y, para el desenlace continuo, fue la diferencia de medias/ *mean difference* (DM). Todos los resultados se informaron con sus respectivos intervalos de confianza (IC) del 95%. Las figuras y gráficos fueron creados por el programa estadístico ( *Software* R *Statistics* ). 

### Métodos de síntesis

 Los datos relacionados con los desenlaces se exportaron al *software R Statistics* , versión 4.2.3, utilizando los paquetes meta versión 6.2-1 y netmeta versión 2.8-1 ( *The R Foundation* , Viena, Austria). Para cada desenlace analizado, un número diferente de estudios comparó directamente nuevas técnicas de inserción (IC-ECG, fórmula, etc.) con la técnica de punción guiada por puntos anatómicos de referencia (comparador común). Se realizó un metaanálisis de intervención con los estudios que se consideraron homogéneos en lo que respecta al diseño del estudio, las características de la muestra y la técnica de inserción realizada, se adoptó un nivel de significancia ≤ 0,05. El comparador común presente en cada estudio permitió comparar simultáneamente todos los tratamientos de interés (técnicas de inserción) mediante un metaanálisis en red. Los análisis se realizaron según el enfoque frecuentista, utilizando el modelo de efectos aleatorios, se usó el método de Mantel-Haenzel para desenlaces binarios y el método de varianza inversa para el desenlace continuo. La heterogeneidad se evaluó en función del I ^2^ , según la clasificación sugerida por PRISMA ^(^ ^18^ ^)^ . 

 Para ordenar en un *ranking* los tratamientos evaluados en cada metaanálisis en red, se estimó el P- *score* , que se basa en la estimación puntual y el error estándar de cada estimación incluida en el metaanálisis en red y mide el grado de certeza de que un tratamiento es mejor que otro. Ese puntaje varía de 0 a 1, y los valores cercanos a 1 indican que del tratamiento bajo evaluación es superior ^(^ ^23^ ^)^ . 

 Los estudios realizados únicamente con prematuros fueron excluidos de los metaanálisis en red, porque se trata de una muestra peculiar y diferente a la que se utiliza en estudios que combinan prematuros con recién nacidos a término, para evitar la intransitividad ^(^ ^17^ ^)^ . Los bebés prematuros tienen anatomía y fisiología inmadura de la piel y del sistema vascular, inmunidad más baja, mayores tasas de infecciones y complicaciones mecánicas ^(^ ^4^ ^,^ ^24^ ^-^ ^28^ ^)^ e, incluso, mayores posibilidades de posicionamiento adecuado de la punta ^(^ ^29^ ^)^ . 

Cabe señalar que el metaanálisis en red no estaba previsto en el protocolo de esta revisión, pero considerando la viabilidad de este análisis y la importancia/relevancia de la evidencia que proporciona, se decidió realizarlo. Esa decisión se basó en el análisis de datos de estudios elegibles, cuando se verificó que las técnicas de inserción evaluadas en los estudios incluidos tenían un grupo control en común. Además, el metaanálisis en red es el método que mejor responde a la pregunta orientadora de esta revisión.

### Evaluación del sesgo de publicación

 Se realizó una búsqueda en la página *Clinicaltrials.gov* de resultados inéditos de ensayos clínicos para reducir el riesgo de sesgo por falta de resultados en una síntesis ^(^ ^17^ ^)^ . No fue posible generar el *funnel plot* , dado que el mismo requiere un mínimo de 10 pruebas para garantizar la potencia adecuada para detectar asimetrías ^(^ ^17^ ^)^ . 

### Evaluación de la certeza de la evidencia

 Se utilizó el enfoque del *Grading of Recommendations, Assessment, Development, and Evaluation Working Group* (GRADE) para evaluar la calidad y certeza de la evidencia para cada desenlace de cada comparación ^(^ ^30^ ^)^ , con una disminución o un aumento de uno o dos puntos según los criterios de evaluación. Se utilizó la versión *web* del GRADEpro para presentar las conclusiones de los principales desenlaces de las comparaciones directas (GRADEpro GDT; *the* GRADE *Working Group* , 2022). La evaluación de la certeza de la evidencia de las estimaciones del efecto obtenidas en el metaanálisis en red se realizó en cuatro etapas con la ayuda de un formulario específico elaborado en *Microsoft Excel* ^®^: 1) presentación de las estimaciones directas e indirectas de cada comparación de interés; 2) graduación de la certeza de la evidencia para cada estimación directa e indirecta; 3) presentación de las estimaciones de los efectos del metaanálisis en red de la comparación de interés; y 4) graduación de la certeza de la evidencia de las estimaciones del metaanálisis en red ^(^ ^31^ ^)^ . En estas etapas se evaluó la intransitividad de las estimaciones indirectas, la incoherencia (o inconsistencia, como la llama PRISMA) y la imprecisión de la red ^(^ ^32^ ^-^ ^34^ ^)^ . 

## Resultados

 Se identificaron un total de 2697 estudios en las bases de datos y literatura gris, de los cuales 1294 eran duplicados, por lo que se seleccionaron 1403 artículos para la lectura de título y resumen. En la Figura 1 se presenta un diagrama de flujo detallado de la selección, exclusión e inclusión de estudios, adaptado de PRISMA. Hay que agregar que, de los 100 registros seleccionados en la literatura gris, siete eran duplicados; los demás se seleccionaron según el título y el resumen, pero no se incluyó ninguno en esta revisión. 

**Figura 1:** - Diagrama de flujo de la búsqueda en la literatura y de los criterios de selección adaptado de PRISMA ^(^ ^18^ ^)^

*CINAHL = *Cumulative Index to Nursing and Allied Health Literature;* ^†^ LILACS = Literatura Latinoamericana y del Caribe en Ciencias de la Salud

 En esta revisión se incluyeron ocho estudios, con una muestra total de 1126 RN. Cinco procedían de China ^(^ ^14^ ^,^ ^28^ ^,^ ^35^ ^-^ ^37^ ^)^ , uno de Estados Unidos ^(^ ^38^ ^)^ , uno de India ^(^ ^39^ ^)^ y uno de Brasil ^(^ ^40^ ^)^ . El período de publicación osciló entre 2013 y 2021, y un estudio estaba disponible solamente en chino ^(^ ^35^ ^)^ . 

 En cuanto al diseño del estudio, seis eran ensayos clínicos controlados aleatorizados ^(^ ^14^ ^,^ ^35^ ^-^ ^36^ ^,^ ^38^ ^-^ ^40^ ^)^ y dos eran ensayos no aleatorizado ^(^ ^28^ ^,^ ^37^ ^)^ . 

 Las muestras variaban dentro de la población de neonatos, tres estudios evaluaron las técnicas de inserción solo en recién nacidos prematuros ^(^ ^28^ ^)^ y muy prematuros/prematuros extremos ^(^ ^38^ ^-^ ^39^ ^)^ y cuatro evaluaron las técnicas de inserción en recién nacidos prematuros y a término ^(^ ^14^ ^,^ ^35^ ^,^ ^37^ ^,^ ^40^ ^)^ . Solo un estudio registró un peso promedio de 2500 g el día de la inserción ^(^ ^36^ ^)^ . 

 Las técnicas de inserción del PICC en RN observadas en los estudios incluidos fueron: electrocardiograma intracavitario (IC-ECG) para guiar la punta ^(^ ^14^ ^,^ ^35^ ^)^ ; electrocardiograma intracavitario con ultrasonido para visualizar la vena (IC-ECG/US) ^(^ ^28^ ^,^ ^37^ ^)^ ; ultrasonido en tiempo real (USTR) para visualizar la punta durante la inserción y guiarla ^(^ ^38^ ^-^ ^39^ ^)^ ; punto anatómico de referencia (PAR) modificado, en el que la intervención se realizó midiendo el tamaño del catéter que se va a insertar con una referencia anatómica diferente ^(^ ^40^ ^)^ ; uso de fórmula, en la que una ecuación determina la cantidad de catéter que se debe insertar junto con la aplicación de una compresa tibia durante 15 minutos en el sitio de inserción antes de la punción ^(^ ^36^ ^)^ ; y punción guiada por puntos anatómicos de referencia, que se caracteriza por punción ciega (sin ayuda de tecnología), medición por PAR (los centímetros de catéter que se van a insertar los predetermina una medición realizada en la superficie del cuerpo desde el sitio probable de inserción hasta la vena cava superior o inferior) y visualización de la punta mediante rayos X ^(^ ^14^ ^,^ ^28^ ^,^ ^35^ ^-^ ^40^ ^)^ , que era el grupo control en todos los estudios. En otras palabras, el comparador/control en todos los estudios era una misma técnica, por ende, la geometría de la red adoptó la forma de estrella. Esto significa que las nuevas técnicas se compararon con un comparador común, es decir, no se realizaron comparaciones directas entre ellas, sino indirectas. 

 El riesgo de sesgo de los estudios incluidos se detalla en la Figura 2 . 

**Figura 2:** - Evaluación del riesgo de sesgo aplicado a los estudios mediante la herramienta RoB2 ( *Risk of Bias* 2) arriba y ROBINS-I ( *Risk of Bias In Non-randomized Studies of Interventions)* abajo. Brasília, DF, Brasil, 2023

 Los ensayos aleatorizados mostraron algunas preocupaciones con respecto al proceso de aleatorización debido a que había algunas diferencias en las características de los participantes ^(^ ^38^ ^)^ , falta de información detallada en el artículo ^(^ ^36^ ^)^ o asignación 1:1 ^(^ ^14^ ^)^ . No fue posible cegar a los profesionales que aplicaron la intervención, dado que debían saber cuál era la intervención para aplicarla, excepto en un estudio ^(^ ^40^ ^)^ . En una investigación que utilizó USTR ^(^ ^38^ ^)^ , se observaron algunas desviaciones de las intervenciones previstas, que pueden haber afectado los desenlaces, y tener fallas de inserción sin explicación que podrían estar directamente relacionadas con la intervención, e incluso modificar el número de participantes en los dos grupos. Si bien no se identificó el protocolo del estudio en una de las publicaciones ^(^ ^39^ ^)^ , lo que llevó a que el dominio de sesgo en los resultados informados fuera considerado “algunas preocupaciones”, este estudio fue evaluado como con bajo riesgo de sesgo debido a que estuvo bien diseñado y a que los autores declararon que todas las intervenciones y análisis se llevaron a cabo según lo planeado antes del inicio del estudio. Dos publicaciones no informaron si los responsables de evaluar los desenlaces sabían o no qué grupo estaban evaluando ^(^ ^35^ ^-^ ^36^ ^)^ . 

 Los ensayos no aleatorizados obtuvieron información de su grupo control a través de datos retrospectivos, y las respuestas a algunas de las preguntas de los dominios fueron “no informado” ^(^ ^28^ ^,^ ^37^ ^)^ . Por ejemplo, no había información sobre si los profesionales que evaluaron los desenlaces sabían a qué grupo pertenecían los participantes, lo que podría haber afectado principalmente la medición del desenlace mal posicionamiento primario de la punta, que fue la única complicación medida en el segundo estudio ^(^ ^37^ ^)^ . Este mismo estudio ^(^ ^37^ ^)^ fue considerado con riesgo crítico de sesgo porque hubo diferencias en las características y en el número de participantes entre los grupos, no hubo ningún tipo de control de los factores de confusión medidos, además parece que los participantes recibieron distinto seguimiento. 

 Las características de los estudios primarios incluidos en la revisión y sus resultados se describen en la Figura 3 . 

**Figura 3:** - Características de los estudios incluidos y resultados individuales obtenidos (n=8). Brasília, DF, Brasil, 2023

**Características del estudio y la muestra**					**Resultados**	**Conclusiones Principales**
**Autor; Año; País**	**Intervenciones (Técnica de Inserción y Control)**	**Muestra (n)**	EGN ^*^ (s ^†^ **) y PN** ^§^ **(g** ^||^ **)**	**EGC** ^‡^ **(s** ^†^ **) o días de vida y/o peso (g** ^||^ **)**	**Complicaciones n (%) experimental vs. n (%) control**	
Katheria, et al.; 2013 ^(^ ^38^ ^)^ ; Estados Unidos	Grupo Experimental: punción ciega, medición por punto anatómico de referencia, USTR ^**^ para localizar la punta	20		EGN ^‡^: 31± 4s ^†^ Peso: 1185 ± 538g ^||^ 4 ±5 días	Malpos. 1 ^o¶^: 5(25%) **vs.** 16(57,1%) Malpos. 2 ^o††^ (Taponamiento cardíaco): 1 (5%) **vs.** 0 (0%)	USTR ^**^ redujo en un 40% el tiempo del procedimiento de inserción ( *p* =0,034), la necesidad de rayos X ( *p* =0,001) y manipulaciones ( *p* =0,034).
	Grupo Control: punción guiada por puntos anatómicos de referencia	28		EGN ^‡^: 30± 4s ^†^ Peso: 1208 ± 481g ^||^ 5 ±5 días		
Ling, et al.; 2019 ^(^ ^14^ ^)^ ; China	Grupo Experimental: punción ciega, medición por punto anatómico de referencia, IC-ECG ^‡‡^ para guiar la punta	80	EGN ^*^: 37,1 ± 1,4s ^†^ (32-40)	3,4 ± 0,5 días (1-6)	Complicaciones generales: 3(3,75%) **vs.** 19(23,75%), *p* =0,000 Arritmia: 0(0%) **vs.** 4(5%), *p* =0,043 Flebitis: 1(1,25%) **vs.** 7(8,75%), *p* =0,03 Infección: 0(0%) **vs.** 1(1,25%), *p* =0,316 Lesiones de la piel: 1(1,25%) **vs.** 1(1,25%), *p* =0,316 Malpos. 1 ^o¶^: 4(5%) **vs.** 17(21,25%), *p* =0,002 Oclusión: 1(1,25%) **vs.** 4(5%), *p* =0,173	IC-ECG ^‡‡^ mejoró la tasa del primer intento de inserción del PICC ^§§^ hasta la posición central, requirió menos tiempo y costos médicos, menor exposición a la radiación y redujo las tasas de complicaciones.
	Grupo Control: punción guiada por puntos anatómicos de referencia	80	EGN ^*^: 36,8 ± 1,3s ^†^ (32-40)	3,2 ± 0,3 días (1-5)		
Oleti, et al.; 2019 ^(^ ^39^ ^)^ ; India	Grupo Experimental: punción ciega, medición por punto anatómico de referencia, USTR ^**^ para localizar la punta	40	EGN ^*^: 31,1± 3,1s ^†^ PN ^§^ 1286g ^||^ (926-1662)	1,75 días (0,83-5,37)	Infección: 4(10,2%) **vs.** 1(2,5%) - RR ^||||^ 4,1; IC ^¶¶^ 95% 0,47-35, *p* =0,24 Infiltración u oclusión: 4(10%) **vs.** 6(15%) Malpos. 1 ^o¶^: 13(32,5%) **vs.** 27(67,5%) – RR ^||||^ 0,48; IC ^¶¶^ 95%: 0,29-0,79; *p* =0,002	El uso de USTR ^**^ redujo la incidencia de mal posicionamiento de la punta en un 52 % y se puede recomendar para la inserción de la punta del PICC ^§§^ .
	Grupo Control: punción guiada por puntos anatómicos de referencia	40	EGN ^*^: 31,4 ±3,6s ^†^ PN ^§^: 1061g ^||^ (889-1636)	1,04 días (0,77-4,87)		
Tang, et al.; 2021 ^(^ ^35^ ^)^ ; China	Grupo Experimental: punción ciega, medición por punto anatómico de referencia, IC-ECG ^‡‡^ para guiar la punta	105	EGN ^*^: 36,9 ± 1,5s ^†^ (33-40)		Complicaciones generales: 4(3,8%) **vs.** 23(21,9%); *p* =0,000 Arritmia: 0(0%) **vs.** 6(5,7%); *p* =0,015 Flebitis: 2(1,9%) **vs.** 9(8,6%); *p* =0,029 Infección: 0(0%) **vs.** 1(1%); *p* =1 Lesiones de la piel: 1(1%) **vs.** 3(2,9%); *p* =0,311 Trombosis: 1(1%) **vs.** 4(3,8%); *p* =0,184	El uso de IC-ECG ^‡‡^ ayuda a detectar y corregir oportunamente la punta ectópica y la lesión vascular resultante, la infección y la arritmia, brindando mayor seguridad que el control.
	Grupo Control: punción guiada por puntos anatómicos de referencia	105	EGN ^*^: 36,4 ± 1,6s ^†^ (32-40)			
Tomazoni, et al.; 2022 ^(^ ^40^ ^)^ ; Brasil	Grupo Experimental: punción ciega, medición por punto anatómico de referencia modificado (sitio de inserción hasta la unión esternoclavicular derecha), rayos X para localizar la punta	44	EGN ^*^ y PN ^§^ están estratificados en franjas		Complicaciones generales: 17(38,6%) **vs.** 27(61,3%) Flebitis: 1(2,3%) **vs.** 3(6,8%) Infiltración: 3(6,8%) **vs.** 6(13,6%) Malpos. 1 ^o^ ^¶^: 23(52,27%) **vs.** 43(97,7%); *p* =0,000 Malpos. 2 ^o††^: 1(2,3%) **vs.** 1(2,3%) Oclusión: 3(6,8%) **vs.** 4(9,1%) Ruptura: 1(2,3%) **vs.** 1(2,3%) Sospecha de infección: 2(4,5%) **vs.** 1(2,3%)	El uso del método de medición modificado proporcionó mejores resultados para el posicionamiento adecuado de la punta del PICC ^§§^ .
	Grupo Control: punción guiada por puntos anatómicos de referencia (sitio de inserción hasta la unión esternoclavicular derecha y hasta el 3º espacio intercostal)	44				
Wu, et al.; 2021 ^(^ ^36^ ^)^ ; China	Grupo Experimental: punción de vena safena después de 15 minutos de compresa tibia, guiada por fórmula, rayos X para localizar la punta *Fórmula >1500g*: L ^***^ = M ^†††^ - 1,5 +23 0,3 *Fórmula <1500g*: L ^***^ = 23 – 1,5 - M ^†††^ 0,3 L ^***^: tamaño del PICC ^§§^ que se va a insertar M ^†††^: peso del recién nacido Grupo Control: punción de la vena safena guiada por puntos anatómicos de referencia	65		16,52± 0,63 días (1,47-26,46) Peso: 2510± 180g ^||^ (1170-3760)	Complicaciones generales: 4(6,15%) **vs.** 23(35,38%); *p* <0,001 Flebitis: 1(1,53%) **vs.** 7(10,76%) Infección: 1(1,53%) **vs.** 6(9,23%) Malpos. 2 ^o††^: 1(1,53%) **vs.** 4(6,15%) Trombosis: 1(1,53%) **vs.** 6(9,23%)	El uso de la fórmula para determinar la cantidad de catéter que se va a insertar redujo el tiempo de hospitalización, la escala del dolor y las complicaciones asociadas, además aumentó la tasa de éxito de la primera punción y el tiempo de permanencia del catéter.
		65		16,73 ± 0,84 días (8,4 -26,81) Peso 2570 ± 210g ^||^ (1190-3890)	Obs: El Malpos. 1 ^o¶^ no se comprobó por individuo, pero sí con respecto a la cantidad de centímetros que hubo que traccionar: Fórmula con tracción de 0,25 ± 0,08 centímetros **vs.** Control con tracción de 2,87 ± 0,23 centímetros ( *p* < 0,000)	
Xiao, et al.; 2019 ^(^ ^28^ ^)^ ; China	Grupo Experimental: US ^‡‡‡^ para visualizar la vena e IC-ECG ^‡‡^ para guiar la punta	78	EGN ^*^: 32,17 ± 2,63s ^†^ (28-37) PN ^§^: 1520 ± 377,38g ^||^	15,21 ±7,52 días Peso 1657,44 ± 307,22g ^||^	Complicaciones generales: 5(6,41%) **vs.** 14(16,86%); *p* =0,040 Flebitis: 2(2,56%) **vs.** 7(10,84%); *p* =0,202 Infección: 1(1,28%) **vs.** 3(3,61%); *p* =0,657 Malpos. 1 ^o¶^: 5(6,41%) **vs.** 22(26,51%); *p* =0,001 Ruptura: 2(2,56%) **vs.** 4(4,82%); *p* =0,735	IC-ECG ^‡‡^ puede contribuir a reducir las tasas de reposicionamiento de la punta y las complicaciones, además de aumentar las tasas de posicionamiento adecuado de la punta en el primer intento.
	Grupo Control: punción guiada por puntos anatómicos de referencia	83	EGN ^*^: 32,36 ± 2,78s ^†^ (28-37) PN ^§^: 1508,13 ± 279,31g ^||^	13,19 ±8,8 días Peso 1571,63 ± 266,16g ^||^		
Zhou et al.; 2017 ^(^ ^37^ ^)^ ; China	Grupo Experimental: US ^‡‡‡^ para visualizar la vena e IC-ECG ^‡‡^ para guiar la punta	49	EGN ^*^: 35 ± 4s ^†^ (28-41)	17 ± 16 días (1-28) PN 2700 ± 900g ^||^ (1100-4900)	Malpos. 1 ^o¶^: 3(6,12%) **vs.** 75(37,5%); *p* <0,001 Malpos. 2 ^o^ ^††^ (Derrame pleural): 0(0%) **vs.** 1(0,5%)	IC-ECG ^‡‡^ con columna salina se puede aplicar a los recién nacidos, guiando la punta del catéter de manera adecuada, reduciendo así los riesgos, retrasos y costos de los ajustes de la punta.
	Grupo Control: punción guiada por puntos anatómicos de referencia	200	EGN ^*^: 36 ± 3s ^†^ (28-41)	13 ± 12 días (1-28) PN 2700 ± 900g ^||^ (1000-5000)		

^‡^ EGN = Edad gestacional corregida;

^**^ USTR = Ultrasonido en tiempo real;

^||||^ RR = Riesgo relativo;

^¶¶^ IC = Intervalo de confianza;

^¶^ Malpos. 1º = Mal posicionamiento primario de la punta;

^***^ L = Tamaño del PICC que se va a insertar;

^†††^ M = Peso del recién nacido;

^§§^ PICC = Catéter central de inserción periférica;

^§^ PN = Peso de nacimiento;

^‡‡‡^ US = Ultrasonido

^‡‡^ IC-ECG = Electrocardiograma intracavitario;

^††^ Malpos. 2º = Mal posicionamiento intravascular o extravascular secundario de la punta;

^*^ EGN = Edad gestacional al nacer;

^†^ s = Semanas;

^||^ g = Gramos;

 No se evaluaron todos los desenlaces en todos los estudios incluidos. Se analizaron en la red los que fueron evaluados en más de un estudio incluido, tenían muestra similar y más de una técnica de inserción. En la estructura o geometría de cada red, el grupo control fue el único que se comparó directamente con las otras técnicas de inserción y estas se compararon indirectamente entre sí, precisamente porque tienen este comparador común. Por lo tanto, para todos los desenlaces en los que fue posible realizar el metaanálisis en red, la geometría fue la misma. En la Figura 4 se representa gráficamente la estructura de la red de todos los desenlaces que permitieron el metaanálisis en red. Cabe destacar que, en la geometría de la red, los círculos representan intervenciones, y el tamaño de los mismo corresponde al número de cada muestra, y las líneas rectas son las comparaciones directas entre las técnicas de inserción, cuanto mayor es el número de estudios que realizaron esa comparación mayor es el grosor de la línea. 

**Figura 4:** - Geometría de la red de todos los desenlaces que permitieron el metaanálisis en redes. Brasilia, DF, Brasil, 2023

^*^ IC-ECG = Electrocardiograma intracavitario; ^†^ PAR = Punto anatómico de referencia; ^‡^ IC-ECG/US = Electrocardiograma intracavitario y ultrasonido para visualizar la vena

 En lo que respecta a los desenlaces primarios, uno de ellos era complicaciones generales, que correspondía al número total de complicaciones informadas por cada estudio, y se pueden referir a dos o más complicaciones, dependiendo del objetivo de la investigación y del tiempo de seguimiento del recién nacido. Dos estudios no verificaron este desenlace ^(^ ^37^ ^-^ ^38^ ^)^ , dado que fueron los únicos que realizaron el seguimiento de la muestra sólo hasta que se confirmó la ubicación de la punta y no hasta que se retiró el PICC (ver tiempo de permanencia del dispositivo), como en los demás. Ninguno de los autores de los estudios analizados incluyó el mal posicionamiento primario de la punta en el desenlace complicaciones generales como lo hicieron con otras complicaciones. La Figura 5 muestra los resultados relacionados con las complicaciones generales con respecto al metaanálisis con comparaciones directas e indirectas. 

**Figura 5:** - Gráficos de bosque que demuestran el efecto de las técnicas de inserción en el desenlace Complicaciones generales, tanto de las comparaciones directas (arriba) como de las realizadas en la red (abajo). Brasilia, DF, Brasil, 2023

^*^ IC-ECG = Electrocardiograma intracavitario; ^†^ RR = Riesgo relativo; ^‡^ IC = Intervalo de confianza; ^§^ PAR = Punto anatómico de referencia; ^||^ USTR = Ultrasonido en tiempo real; ^¶^ IC-ECG/US = Electrocardiograma intracavitario y ultrasonido para visualizar la vena

 La complicación flebitis se analizó con metaanálisis de intervención y en red con los estudios con la misma muestra. En la intervención con los dos estudios que utilizaron IC-ECG en la inserción del PICC ^(^ ^14^ ^,^ ^35^ ^)^ , se observó que esta técnica podría reducir significativamente el riesgo de flebitis hasta en un 81% (RR 0,19; IC 95%: 0,06-0,65). En el metaanálisis en red, el IC-ECG, cuando se lo comparó con el control, fue la única técnica de inserción capaz de reducir significativamente esta complicación. 

 El mal posicionamiento primario de la punta del PICC fue un desenlace que no se midió directamente en dos estudios ^(^ ^35^ ^-^ ^36^ ^)^ . Todas las intervenciones (IC-ECG, IC-ECG/US, USTR y PAR modificado) redujeron el riesgo de mal posicionamiento primario de la punta, con una estimación de gran magnitud con respecto al control. Se realizó un metaanálisis de intervención con dos estudios que compararon el USTR con el grupo control ^(^ ^38^ ^-^ ^39^ ^)^ , con RR de 0,47 (IC 95%: 0,31-0,72). Sin embargo, como estos ensayos ^(^ ^38^ ^-^ ^39^ ^)^ se realizaron con recién nacidos muy prematuros y prematuros extremos, no fue posible incluirlos en el metaanálisis en red realizado con otras técnicas de inserción. En esta red, también se observó que el IC-ECG/US redujo el riesgo de mal posicionamiento primario con respecto al PAR modificado (RR: 0,31; IC del 95 %: 0,10-0,96). 

 Por lo tanto, las técnicas de inserción redujeron significativamente las complicaciones generales, la flebitis y el mal posicionamiento primario de la punta. En la Figura 6 se presenta el *ranking* realizado según los resultados del P- *score* de las técnicas más efectivas para reducir estas complicaciones. En esta figura, los riesgos relativos (RR) inferiores a 1 favorecen la intervención definida en la columna de resultados del metaanálisis en red (triángulo inferior) y la intervención definida en la fila para los resultados del metaanálisis pareado (triángulo superior). Por ende, parece que las técnicas de inserción más eficaces para reducir las complicaciones generales, la flebitis y el mal posicionamiento primario de la punta fueron, respectivamente, IC-ECG (P- *score* 0,8399), fórmula (P- *score* 0,7562) e IC-ECG/US (P- *score* 0,8863). 

**Figura 6:** - Tablas de posiciones *(League tables)* que presentan el *ranking* de las técnicas de inserción más eficaz para reducir las Complicaciones generales, la flebitis y el mal posicionamiento primario de la punta con los respectivos riesgos relativos como resultados de las comparaciones. Brasilia, DF, Brasil, 2023

^*^ IC-ECG = Electrocardiograma intracavitario; ^†^ PAR = Punto anatómico de referencia; ^‡^ IC-ECG/US = Ultrasonido para visualizar la vena y la punta guiada por electrocardiograma intracavitario

 La arritmia se investigó a través de un metaanálisis de intervención realizado con los dos únicos estudios ^(^ ^14^ ^,^ ^35^ ^)^ que midieron ese desenlace, y se comprobó que la técnica de inserción IC-ECG (RR 0,09; IC 95%: 0,01-0,71) redujo el riesgo de la misma. 

 Con los desenlaces infección, mal posicionamiento secundario de la punta y trombosis venosa profunda asociada al catéter, se realizó un metaanálisis de intervención y un metaanálisis en red, no se obtuvo significación estadística en las estimaciones del efecto pareadas y de la red. Se realizó un *ranking* de las técnicas de inserción más efectivas según la tendencia de reducción de dichas complicaciones y se consideró que la fórmula era la mejor. 

 Dos de las complicaciones mencionadas en el manual de la INS 2021 no fueron encontradas en ninguno de los estudios incluidos en esta revisión: punción arterial inadvertida y embolia aérea ^(^ ^11^ ^)^ . En los estudios incluidos se mencionaron otras dos complicaciones: oclusión y lesiones de la piel asociadas con el catéter. Los metaanálisis realizados para estos dos últimos desenlaces (en red para oclusión e intervención para lesiones de la piel) no mostraron una reducción significativa según la técnica de inserción utilizada. 

 En cuanto a los desenlaces secundarios, la tasa de éxito general (TEG) estuvo por encima del 80% en un estudio que utilizó USTR (92,5% vs. 85%) ^(^ ^39^ ^)^ . Se llevó a cabo un metaanálisis de intervención con este desenlace con solo dos estudios homogéneos que utilizaron USTR ^(^ ^38^ ^-^ ^39^ ^)^ en el grupo experimental, que dio como resultado un RR 0,90; IC 95%: 0,60-1,36. 

 El estudio con mayores tasas de éxito en la primera punción fue el que utilizó la fórmula como técnica de inserción (96,92% vs. 72,31%) ^(^ ^36^ ^)^ , mientras que con el PAR modificado la tasa alcanzó el 37,5% ^(^ ^40^ ^)^ . 

 En lo que respecta al tiempo de permanencia del catéter, la fórmula ^(^ ^36^ ^)^ y el USTR ^(^ ^39^ ^)^ revelaron que el grupo experimental tuvo un tiempo de permanencia significativamente mayor que el control (DM 2,35; IC 95%: 2,07-2,63 y DM 0,9; IC 95%: 0,60-1,10, respectivamente). La técnica de inserción IC-ECG redujo la duración promedio del tiempo de permanencia (DM -0,23; IC del 95%: -1,21-0,75). 

 La certeza del conjunto de evidencias se realizó a través del GRADE. Ninguno de los desenlaces evaluados presentó una “alta” certeza de la evidencia. Se evaluaron las comparaciones directas e indirectas para cada desenlace, para obtener un resultado de la certeza de la evidencia de los metaanálisis en rede a partir de la evaluación de la incoherencia e imprecisión de estas redes. La inconsistencia en la red, como la llama el GRADE, no existe, porque, para cada desenlace, había comparaciones directas (técnicas vs. control) o comparaciones indirectas (entre técnicas en la red), pero nunca una combinación comparación directa e indirecta. Los resultados de la evaluación se presentan en la Figura 7 . 

**Figura 7:** - Resultados de las evaluaciones de la certeza de la evidencia a partir de las estimaciones directas, indirectas y del metaanálisis en red, según el *Grading of Recommendations, Assessment, Development, and Evaluation Working Group* (GRADE) realizado en los desenlaces (n=11) con sus respectivas comparaciones. Brasilia, DF, Brasil, 2023

**Desenlaces**	**Comparaciones**	**Certeza de la evidencia de las estimaciones directas**	**Certeza de la evidencia de las estimaciones indirectas**	**Certeza de la evidencia del metaanálisis en red**
Arritmia	IC-ECG ^*^ vs. Control	Moderada	-	-
Complicaciones generales	IC-ECG ^*^ vs. Control Fórmula vs. Control PAR ^†^ modificado vs. Control IC-ECG/US ^‡^ vs. Control USTR ^§^ vs. Control IC-ECG ^*^ vs. Fórmula IC-ECG ^*^ vs. PAR ^†^ modificado Fórmula vs. PAR ^†^ modificado	Moderada Moderada Moderada Baja Muy baja - - -	- - - - - Moderada Moderada Moderada	Moderada Baja Baja - - Baja Moderada Baja
Flebitis	IC-ECG ^*^ vs. Control PAR ^†^ modificado vs. Control Fórmula vs. Control Fórmula vs. PAR ^†^ modificado Fórmula vs. IC-ECG ^*^ IC-ECG ^*^ vs. PAR ^†^ modificado	Moderada Moderada Moderada - - -	- - - Moderada Moderada Moderada	Moderada Muy baja Baja Muy baja Muy baja Muy baja
Infección	IC-ECG ^*^ vs. Control PAR ^†^ modificado vs. Control Fórmula vs. Control USTR ^§^ vs. Control Fórmula vs. PAR ^†^ modificado Fórmula vs. IC-ECG ^*^ IC-ECG ^*^ vs. PAR ^†^ modificado IC-ECG/US ^‡^ vs. Control	Moderada Baja Moderada Muy baja - - - Muy baja	- - - - Baja Moderada Baja -	Muy baja Muy baja Baja - Muy baja Muy baja Muy baja -
Lesiones de la piel	IC-ECG ^*^ vs. Control	Muy baja	-	-
Mal posicionamiento primario	IC-ECG ^*^ vs. Control PAR ^†^ modificado vs. Control IC-ECG/US ^‡^ vs. Control USTR ^§^ vs. Control IC-ECG/US ^‡^ vs. PAR ^†^ modificado IC-ECG/US ^‡^ vs. IC-ECG ^*^ IC-ECG ^*^ vs. PAR ^†^ modificado	Moderada Moderada Muy baja Moderada - - -	- - - - Baja Baja Moderada	Moderada Moderada Baja - Baja Muy baja Muy baja
Mal posicionamiento secundario	PAR ^†^ Modificado vs. Control Fórmula vs. Control IC-ECG/US ^‡^ vs. Control USTR ^§^ vs. Control Fórmula vs. IC-ECG/US ^‡^ Fórmula vs. PAR ^†^ modificado PAR ^†^ modificado vs. IC-ECG/US ^‡^	Moderada Baja Muy baja Muy baja - - -	- - - - Muy baja Baja Muy baja	Muy baja Muy baja Muy baja - Muy baja Muy baja Muy baja
Oclusión	IC-ECG ^*^ vs. Control PAR ^†^ modificado vs. Control IC-ECG ^*^ vs. PAR ^†^ modificado	Moderada Moderada -	- - Moderada	Muy baja Muy baja Muy baja
Tiempo de permanencia	IC-ECG ^*^ vs. Control Fórmula vs. Control USTR ^§^ vs. Control IC-ECG ^*^ vs. Fórmula	Moderada Moderada Moderada -	- - - Moderada	Muy baja Moderada - Moderada
Trombosis	IC-ECG ^*^ vs. Control Fórmula vs. Control Fórmula vs. IC-ECG ^*^	Alta Moderada -	- - Moderada	Baja Muy baja Muy baja
Tasa de éxito general	PAR ^†^ modificado vs. Control USTR ^§^ vs. Control	Baja Muy baja	- -	- -

^‡^ IC-ECG/US = Electrocardiograma intracavitario con ultrasonido para visualización;

^*^ IC-ECG = Electrocardiograma intracavitario;

^†^ PAR = Punto anatómico de referencia;

^§^ USTR = Ultrasonido en tiempo real

## Discusión

Esta revisión es la primera en verificar la efectividad de diferentes técnicas de inserción de PICC para prevenir la aparición de complicaciones en los recién nacidos. Con la síntesis, evaluación y combinación cuidadosa de los ocho estudios incluidos a través de metaanálisis pareados y en red, se pudo comprobar qué técnica de inserción, entre las identificadas, es más efectiva para reducir ciertas complicaciones asociadas al dispositivo de acceso venoso central PICC. Por ende, se registró una reducción significativa del riesgo de complicaciones generales, arritmias, flebitis, mal posicionamiento primario de la punta del PICC, así como cambios en el tiempo de permanencia del catéter dependiendo de la técnica de inserción utilizada. La incidencia de infección, infiltración/extravasación, mal posicionamiento secundario de la punta, rotura del catéter, trombosis, oclusión, lesiones de la piel y TEG se vio afectada por las técnicas de inserción y la mayoría registró una reducción absoluta, pero sin significación estadística y presentó estimaciones de efecto con muy baja certeza de la evidencia.

 La prevención de complicaciones puede reducir la morbilidad, la necesidad de procedimientos adicionales y los costos ^(^ ^41^ ^)^ . Las buenas prácticas durante la inserción del PICC en recién nacidos pueden disminuir la exposición innecesaria del bebé, las múltiples venopunciones y el riesgo de posicionamiento inadecuado del catéter, además de mejorar la tasa de éxito de la inserción ^(^ ^42^ ^-^ ^43^ ^)^ . 

En esta revisión, el uso de IC-ECG se asoció con un riesgo significativamente menor y más efectivo de complicaciones generales, flebitis y mal posicionamiento primaria de la punta y con una reducción de las arritmias cardíacas, todo con una certeza de la evidencia moderada. También fue la mejor técnica para reducir el tiempo de permanencia del catéter, especialmente cuando se la compara con la técnica que utiliza la fórmula. Debido a la moderada certeza de los hallazgos, se puede decir que el IC-ECG es una técnica de inserción que se debe utilizar en la práctica clínica por su eficacia para prevenir complicaciones importantes.

 La revisión sistemática china y los metaanálisis realizados con ensayos clínicos aleatorizados locales comprobaron que el IC-ECG redujo el riesgo de flebitis ( *Odds Ratio* - OR 0,33 - IC 95% 0,19-0,56, p<0,001) y el total de complicaciones (OR 0,23 - IC 95% 0,16-0,33, p<0,001), además aumentó la precisión de la localización óptima de la punta (OR 5,37 - IC 95% 3,80-7,59, p<0,001) ^(^ ^44^ ^)^ . Los resultados de ese metaanálisis coinciden con los obtenidos en el presente estudio, que demuestra la efectividad del IC-ECG para reducir arritmias, complicaciones generales, flebitis, además del mal posicionamiento primario de la punta y sus posibles consecuencias. Esto se debe a que las investigaciones y el conjunto de buenas prácticas asociaron el aumento de las tasas de complicaciones con la punta del PICC no central, especialmente oclusión, extravasación, otras complicaciones mecánicas, flebitis y fracaso/extracción no electiva ^(^ ^2^ ^,^ ^11^ ^,^ ^29^ ^,^ ^41^ ^,^ ^45^ ^-^ ^46^ ^)^ . La tasa de complicaciones de los PICC no centrales es hasta un 200% mayor que las de los centrales ^(^ ^29^ ^)^ . En otras palabras, una técnica de inserción capaz de prevenir un mal posicionamiento primario del dispositivo de acceso venoso central venoso central reducirá la aparición de estas otras complicaciones mencionadas anteriormente, que pueden ser extremadamente dañinas. 

 La incidencia de flebitis se relaciona con la técnica de inserción del PICC, ya que puede ocurrir cuando la punta no se ubica en posición central ^(^ ^2^ ^,^ ^35^ ^,^ ^47^ ^)^ (los vasos de menor calibre están más sujetos a la irritación química y la fricción constante del catéter). También puede estar asociada a otros factores como catéter muy rígido, técnica de punción inadecuada, sitio de punción inadecuado por movimiento constante de la extremidad (especialmente de las superiores), infección e incompatibilidad de medicamentos ^(^ ^11^ ^,^ ^35^ ^,^ ^48^ ^-^ ^49^ ^)^ . Por lo tanto, dado que la técnica de inserción de IC-ECG redujo con mayor eficacia el mal posicionamiento de la punta, no nos sorprende que sea la mejor para reducir el riesgo de flebitis. 

 En cuanto al tiempo de permanencia (TP), si bien el IC-ECG no fue eficaz para reducirlo en comparación con el control, sí lo redujo significativamente con respecto a la fórmula. Este resultado no coincide con los de la revisión sistemática y los del metaanálisis chino, en los cuales el uso de IC-ECG durante la inserción redujo el tiempo de permanencia en comparación con el control (DM = 5,86, IC 95 % 16,36-4,65, p = 0,00) ^(^ ^44^ ^)^ . Cabe destacar que el tiempo que el catéter estuvo colocado sólo debe discutirse si se agregan otros datos que no se recopilaron y no estuvieron presentes en los estudios incluidos en esta revisión. Esto se debe a que la interpretación de la disminución del TP puede ser positiva o negativa, es positiva si se produjo debido a una reducción de las complicaciones y, por ende, a que la terapia intravenosa duró menos, y es negativa si esto se produjo debido a que el catéter se retiró antes de tiempo. 

 La técnica de inserción que utiliza una fórmula basada en el peso del recién nacido para predecir el tamaño, en centímetros, del catéter que se va a insertar ( Figura 3 ) demostró ser superior a las demás al aumentar significativamente el tiempo de permanencia (certeza de la evidencia moderada) y reducir el riesgo de flebitis (baja confianza en el efecto). Esta baja certeza de la evidencia con respecto a la flebitis lleva a considerar que la técnica más eficaz para reducir el riesgo fue, de hecho, el IC-ECG, en el que la confianza es moderada, con estimaciones más precisas. Además, fue la segunda mejor técnica para reducir el riesgo de complicaciones generales y la que tuvo mejor tasa de éxito en el primer intento de punción, menor necesidad de ajustes del PICC, tiempo de hospitalización y dolor ^(^ ^36^ ^)^ . 

 Otras investigaciones con recién nacidos también dieron como resultado fórmulas que predicen el tamaño del catéter que se va a insertar, en función de las características físicas de los bebés como peso, estatura y edad gestacional, que garantizan una forma más precisa para ubicar de forma central la punta del PICC ^(^ ^25^ ^,^ ^50^ ^)^ . 

 En el estudio que utilizó la fórmula, la punción de la vena safena fue realizada casi siempre en el primer intento y por enfermeros capacitados y experimentados ^(^ ^36^ ^)^ . La experiencia de un equipo de enfermería vascular es importante para una inserción exitosa y la seguridad en términos de tasas de complicaciones ^(^ ^28^ ^,^ ^51^ ^)^ . 

 Cabe destacar que, además de la compresa tibia utilizada en investigaciones con la fórmula ^(^ ^36^ ^)^ , otras investigaciones probaron diferentes estrategias para mantener al RN caliente y las venas vasodilatadas durante la inserción del PICC, como, uso de una manta térmica ^(^ ^52^ ^)^ y colocación del RN en una cuna con calor radiante durante la inserción de este dispositivo ^(^ ^4^ ^)^ . Estas tácticas y los resultados obtenidos del número de inserciones en la primera punción en el estudio que utilizó la compresa tibia (96,92%, n=63) ^(^ ^36^ ^)^ y la cuna con calor radiante (77,72%, n=457) ^(^ ^4^ ^)^ demuestran que es realmente necesario emplear medidas para evitar la hipotermia del recién nacido durante procedimientos prolongados como la inserción del PICC. 

 El IC-ECG junto con el US para la visualizar la vena se consideró la mejor técnica de inserción para reducir el mal posicionamiento primario de la punta según el *ranking* del metaanálisis en red. Sin embargo, hay que considerar que los estudios que lo utilizaron fueron no aleatorizados, con un riesgo de sesgo mayor al moderado y una certeza de la evidencia muy baja y baja. Por lo tanto, es correcto decir que la técnica más eficaz para reducir el riesgo de mal posicionamiento primario fue el IC-ECG solo y que el IC-ECG/US fue la segunda mejor, superando al PAR modificado y al control. Los otros desenlaces medidos en los estudios que utilizaron IC-ECG/US ^(^ ^28^ ^,^ ^37^ ^)^ también obtuvieron estimaciones con un grado significativo de incertidumbre y sin significancia estadística. Esto demuestra que el IC-ECG/US, en lo que respecta a la prevención de complicaciones asociadas al PICC, o no presenta ventajas en la práctica clínica para este fin o requiere que se realicen estudios aleatorizados para verificar de forma más confiable la existencia o no de beneficios. 

 Sin embargo, el uso de equipos que contribuyan a la visualización y la venopunción puede ser útil, dado que los recién nacidos tienen venas más estrechas que son inmaduras y vulnerables a roturas ^(^ ^1^ ^,^ ^25^ ^-^ ^26^ ^)^ . En la investigación con PAR modificado también hubo fracaso en la punción de 18 recién nacidos para la inserción del PICC debido a la fragilidad venosa ^(^ ^40^ ^)^ . 

 El INS recomienda el uso de US para evaluar el calibre de la vena que en la que realizará la punción (el catéter no puede exceder el 45% del vaso), si existen anomalías como oclusiones y trombosis, y para identificar la posición de la punta del dispositivo ^(^ ^11^ ^)^ . En una investigación descriptiva realizada por enfermeras de Irán con 30 recién nacidos, se utilizó un dispositivo US para evaluar y visualizar la vena durante la punción para insertar el PICC. Hubo un aumento en la tasa de éxito en el primer (68% grupo US vs. 60% grupo convencional) y segundo intento (50% vs. 40%), pero esto no era estadísticamente significativo ^(^ ^53^ ^)^ . Un estudio retrospectivo estadounidense cuyo objetivo fue describir el uso de US en la inserción del PICC obtuvo una tasa de éxito del 100% en una muestra compuesta por 10 recién nacidos con múltiples intentos fallidos de acceso vascular ^(^ ^54^ ^)^ . 

 La técnica de inserción que utilizó PAR modificado ^(^ ^40^ ^)^ redujo el riesgo de complicaciones generales y mal posicionamiento primario con significación estadística (certeza de la evidencia baja y moderada a muy baja, respectivamente). 

 Un estudio retrospectivo realizado con 588 RN obtuvo una baja prevalencia de complicaciones asociadas al catéter (10,71%) y también realizó mediciones hasta la unión esternoclavicular derecha, al igual que el estudio con PAR modificado ^(^ ^40^ ^)^ , y confirmó que la medición tradicional terminó hacia arriba colocando la punta PICC demasiado profunda ^(^ ^4^ ^)^ . Por lo tanto, es imperativo, cuando se trata de RN, realizar una medición previa de la cantidad de catéter que se va a introducir en base a puntos anatómicos de referencia, diferente a la que recomienda el INS para otras poblaciones en los miembros superiores (lugar de punción, región esternoclavicular derecha y tercer espacio intercostal) ^(^ ^11^ ^)^ . 

 El USTR demostró ser efectivo para reducir el mal posicionamiento primario de la punta y el aumento del tiempo de permanencia del catéter de forma significativa y con una certeza baja y moderada en los hallazgos, respectivamente. No presentó estimaciones de efecto significativas para el resto de las complicaciones e incluso obtuvo estimaciones opuestas, sin significancia y con muy baja certeza en algunos casos, como complicaciones generales, infección y mal posicionamiento secundario de la punta. Hay que considerar que los estudios que utilizaron esta técnica de inserción ^(^ ^38^ ^-^ ^39^ ^)^ fueron realizados con recién nacidos muy prematuros y prematuros extremos, cuyas peculiaridades pueden haber provocado que se registre una tendencia al aumento de algunas complicaciones o a la falta de reducción significativa de otras, como ocurrió con las otras técnicas. Además, eran los estudios que tenían menor tamaño de muestra. 

 Una cohorte prospectiva siguió la inserción de PICC en las extremidades inferiores de 166 recién nacidos en el grupo experimental con USTR y la comparó con 141 recién nacidos en los que no se utilizó esta tecnología. Al igual que en la presente revisión, hubo una reducción significativa en el mal posicionamiento primario de la punta, dado que la necesidad de ajustes del catéter en el grupo USTR fue mucho menor (10,84% vs. 65,95%, p<0,001) y no hubo diferencias significativas entre los grupos en el resto de las complicaciones (flebitis, oclusión, bacteriemia y rotura) ^(^ ^12^ ^)^ . 

 Un metaanálisis reciente también valida los resultados encontrados en esta revisión al comprobar que el US es excelente para ubicar la punta del PICC en una unidad neonatal en comparación con los rayos X, que tiene una sensibilidad del 95,2% y una especificidad del 71,4%, y que hay que combinarlo con las radiografías cuando no se puede localizar la punta por ese método ^(^ ^55^ ^)^ . 

 Con los resultados negativos de los estudios que utilizaron USTR, queda claro que es imprescindible establecer protocolos ^(^ ^56^ ^-^ ^57^ ^)^ , así como una adecuada capacitación y la experiencia de los profesionales que manejan los equipos ^(^ ^57^ ^)^ . En el caso de recién nacidos y niños, se consideró una desventaja la necesidad de estos requisitos para el US, al igual que las dificultades que tienen algunas unidades para contar con un dispositivo disponible las 24 horas del día ^(^ ^57^ ^-^ ^59^ ^)^ . 

 Se han realizado varios cambios en los materiales y procedimientos relacionados con la inserción y el mantenimiento del PICC en recién nacidos y con el uso de tecnologías que ayuden a realizar la punción y, en este caso, la posición central del PICC ha sido la más recomendada en la literatura actual ^(^ ^11^ ^,^ ^56^ ^,^ ^60^ ^-^ ^61^ ^)^ . Estas tecnologías tienen como objetivo reducir las tasas de complicaciones y, por ende, el dolor, el sufrimiento y la morbilidad y mortalidad de los recién nacidos ^(^ ^60^ ^)^ . Sin embargo, ninguno de los estudios brasileños publicados recientemente ^(^ ^26^ ^,^ ^42^ ^,^ ^62^ ^)^ registró el uso de estas tecnologías (IC-ECG, USTR, US para visualizar la vena, etc.), lo que nos lleva a reflexionar sobre cuales son los motivos que han contribuido a que no se usen ni estudien en Brasil. 

 Esta revisión sistemática y metaanálisis (pareado y en red) comprobó la efectividad de las técnicas de inserción en la prevención de la aparición de complicaciones asociadas con el PICC en recién nacidos, que pueden incluso provocar secuelas y la muerte. La misma incluyó alrededor de seis técnicas de inserción, más de 1000 RN en la muestra, utilizada con rigor metodológico y análisis diferenciados. Los estudios incluidos fueron todos ensayos clínicos controlados, aleatorizados y no aleatorizados, evaluados en conjunto, como lo recomienda la literatura ^(^ ^63^ ^)^ , la mayoría de ellos tenía un tamaño de muestra mayor a 100 recién nacidos. Este hecho puede considerarse una ventaja, dado que los ensayos clínicos relacionados con catéteres intravenosos en recién nacidos generalmente tienen un tamaño de muestra promedio inferior a 100 participantes ^(^ ^64^ ^)^ . 

Sin embargo, esta revisión debe interpretarse en el contexto de algunas limitaciones. Se evaluó una pequeña muestra de estudios comparativos, y se realizaron pocos metaanálisis de intervención. Además, ninguno de los estudios comparó diferentes tecnologías entre sí para la inserción de PICC, el riesgo de sesgo de la mayoría de los estudios incluidos fue “algunas preocupaciones” y ninguno de los desenlaces obtuvo en la evaluación certeza de la evidencia alta.

## Conclusión

Esta revisión demostró que las técnicas de inserción son capaces de prevenir complicaciones asociadas con el PICC en recién nacidos, pero aún no es posible decir cuál es la más efectiva para todas ellas. Esto se debe a que cada técnica incluida en esta revisión mostró mayor efectividad para diferentes complicaciones, aunque el IC-ECG redujo la mayoría de ellas.

Todas las técnicas distintas a la punción guiada por puntos anatómicos de referencia (control) redujeron el mal posicionamiento primario de la punta, pero el IC-ECG fue la más efectiva para reducirlo junto con las complicaciones generales, la flebitis y el tiempo de permanencia del catéter, y redujo también de forma considerable y significativa las arritmias. La fórmula demostró que tenía mayor eficacia para aumentar el tiempo de permanencia del catéter y fue la segunda mejor para reducir las complicaciones generales. También se consideró superior con una certeza de la evidencia muy baja y sin significación estadística para reducir el riesgo de infección, el mal posicionamiento secundario de la punta y la trombosis. El USTR no mostró una reducción del resto de las complicaciones, aumentó el tiempo de permanencia del catéter y demostró una tendencia a aumentar algunas sin significancia.

Es necesario que se realicen más ensayos clínicos controlados aleatorizados con muestras más grandes que comparen las técnicas de inserción en términos de tasas de complicaciones. También hay que implementar estudios de costo-efectividad para que se puedan desarrollar políticas públicas que reduzcan las fallas en la terapia intravenosa para pacientes de todas las edades y que brinden mayor seguridad. Todos los esfuerzos deben dirigirse a que se investiguen más estrategias, técnicas y tecnologías para que el uso del PICC, esencial para los recién nacidos, sea más seguro para esta población tan vulnerable.

# Translation: Prevenção de complicações relacionadas a técnicas de inserção do cateter central de inserção periférica em recém-nascidos: revisão sistemática e metanálise em rede ^*^

**DOI:** 10.1590/1518-8345.6905.4162

**Authors:** Ludmylla de Oliveira Beleza, Guilherme da Costa Brasil, Amanda Salles Margatho, Christiane Inocêncio Vasques, Renata Cristina de Campos Pereira Silveira, Priscilla Roberta Silva Rocha, Laiane Medeiros Ribeiro

**Affiliations:** ^1^ Universidade de Brasília, Brasília, DF, Brasil.; ^2^ Secretaria do Estado de Saúde, Hospital Materno Infantil de Brasília, UTI Neonatal, Brasília, DF, Brasil.; ^3^ Centro Universitário do Distrito Federal, Brasília, DF, Brasil.; ^4^ Universidade de São Paulo, Escola de Enfermagem de Ribeirão Preto, PAHO/WHO Collaborating Centre for Nursing Research Development, Ribeirão Preto, SP, Brasil.; ^5^ Universidade de Brasília, Faculdade de Ciências da Saúde, Brasília, DF, Brasil.; ^6^ Universidade de Brasília, Faculdade de Ceilândia, Brasília, DF, Brasil.

**Keywords:** Recém-Nascido, Cateterismo Periférico, Cateteres Venosos Centrais, Falha de Tratamento, Enfermagem Neonatal, Unidades de Terapia Intensiva Neonatal

## Abstract

**Objetivo:**

analisar a efetividade das técnicas de inserção de cateter central de inserção periférica na prevenção da ocorrência de complicações relacionadas a este dispositivo em recém-nascidos.

**Método:**

revisão sistemática da literatura e metanálise pareada e em rede, com busca realizada em sete bases de dados e na literatura cinzenta, inclusão de ensaios clínicos aleatorizados e não aleatorizados. O risco de viés foi avaliado pelas ferramentas da Cochrane *Risk of Bias 2* e o *Risk of Bias In Non-randomised Studies of Interventions.* A certeza da evidência pelo *Grading of Recommendations Assessment, Development and Evaluation.* Realizou-se metanálise com auxílio do programa estatístico R.

**Resultados:**

oito estudos, com 1126 recém-nascidos, foram incluídos e seis técnicas de inserção identificadas: eletrocardiograma intracavitário, eletrocardiograma intracavitário associado à ultrassonografia, ultrassonografia, fórmula, marco de referência anatômico e marco de referência anatômico modificado. Cinco técnicas diminuíram significativamente o mau posicionamento primário da ponta quando comparadas com o controle ( *p* <0,05). O eletrocardiograma intracavitário diminuiu arritmias, complicações gerais e flebite de forma significativa e mais efetiva; a técnica que utilizou uma fórmula também reduziu complicações gerais. Infecção, infiltração, mau posicionamento secundário da ponta, ruptura do cateter, trombose, oclusão e lesão de pele associada ao cateter não foram eventos prevenidos significativamente.

**Conclusão:**

eletrocardiograma intracavitário e uso da fórmula foram as técnicas mais efetivas na redução de complicações.

## Destaques

**(1)** Algumas técnicas de inserção de PICC reduziram suas complicações relacionadas.

**(2)** O mau posicionamento primário da ponta foi a complicação mais prevenida.

**(3)** O eletrocardiograma intracavitário diminuiu arritmias, flebite e complicações gerais.

**(4)** A técnica que usou a fórmula reduziu complicações gerais significativamente.

## Introdução

 O cateter central de inserção periférica (CCIP), na língua inglesa denominado *Peripherally Inserted Central Catheter* (PICC), é amplamente utilizado em recém-nascidos (RNs) ^(^ ^1^ ^-^ ^2^ ^)^ . Ele apresenta várias vantagens em relação ao uso de outros dispositivos de acesso vascular central e de cateteres curtos de inserção periférica, tais como preservação do sistema vascular periférico ^(^ ^3^ ^)^ , redução do risco de flebite e infecções ^(^ ^3^ ^-^ ^4^ ^)^ , da necessidade de repetidas punções ^(^ ^4^ ^-^ ^5^ ^)^ e da dor e estímulos a que os RNs são submetidos ^(^ ^3^ ^-^ ^5^ ^)^ . 

 Apesar das vantagens, o uso do PICC está associado a diferentes tipos de complicações que podem aumentar a morbimortalidade neonatal ^(^ ^6^ ^-^ ^7^ ^)^ , elevar os custos para o sistema de saúde e comprometer a qualidade de vida dos pacientes ^(^ ^8^ ^-^ ^10^ ^)^ . 

 As complicações relacionadas ao PICC podem ocorrer desde a sua inserção, perpassando pelo período de permanência e, até mesmo, após sua retirada ^(^ ^6^ ^)^ . Segundo a *Infusion Nursing Society* (INS) (2021), as complicações relacionadas à inserção de dispositivos de acesso vascular central como o PICC são: punção arterial, arritmias cardíacas, lesão de nervos, embolia aérea e mau posicionamento primário do dispositivo de acesso vascular central ^(^ ^11^ ^)^ . Muitas complicações como trombose venosa profunda associada ao cateter, mau posicionamento secundário (migração da ponta do cateter intra ou extravascular), infecção da corrente sanguínea associada à linha central, flebite, infiltração, extravasamento, ruptura de cateter, oclusão e lesão de pele associada ao dispositivo poderiam ser prevenidas a depender da técnica de inserção utilizada ^(^ ^11^ ^)^ . 

 Do ponto de vista da técnica de inserção, alguns cuidados, como o método utilizado para guiar a inserção, interferem nas complicações relacionadas ao PICC, ou seja, ações realizadas durante a inserção deste dispositivo podem prevenir as complicações relacionadas à sua introdução e as tardias que ocorrem com a permanência do PICC ^(^ ^11^ ^)^ . 

 Estudos e conjuntos de boas práticas recomendam o uso de tecnologias na inserção do PICC de modo a prevenir as complicações relacionadas e aumentar a segurança do paciente, como ultrassonografia (USG) ^(^ ^11^ ^-^ ^12^ ^)^ , relação cateter-veia ^(^ ^11^ ^)^ e eletrocardiograma intracavitário (IC-ECG) ^(^ ^11^ ^,^ ^13^ ^-^ ^14^ ^)^ . Para as instituições de saúde, há redução dos custos relacionados ao tratamento das complicações, visto que, em algumas situações, a retirada deste dispositivo requer outra punção e, muitas vezes, é necessário o uso de novos cateteres, além do aumento do tempo de enfermagem dispendido ^(^ ^5^ ^,^ ^10^ ^-^ ^11^ ^)^ . 

 A USG pode ser utilizada para determinar o tamanho ideal do cateter a ser introduzido de acordo com o diâmetro da veia (relação cateter-veia), para localizar a ponta e para visualização vascular para guiar a punção. Isso pode reduzir as complicações e necessidade de reposicionamento, bem como aumentar as chances de sucesso na primeira tentativa de punção e proporcionar maior satisfação do paciente ^(^ ^11^ ^-^ ^12^ ^,^ ^15^ ^)^ . 

 O IC-ECG, por meio de eletrodos na superfície corporal e em contato com o cateter, monitoriza alterações no tamanho da onda P do eletrocardiograma, cuja elevação indica que a ponta do cateter alcançou a junção cavoatrial através da veia cava superior ^(^ ^14^ ^)^ . Esta técnica mostra, em tempo real, a correta posição da ponta do dispositivo, levando a posicionamentos centrais significativamente mais bem-sucedidos devido ao seu ajuste imediato, além de proporcionar tratamento imediato e eliminar a necessidade de confirmação do posicionamento da ponta por exame radiológico ^(^ ^13^ ^-^ ^14^ ^,^ ^16^ ^)^ . 

Estudos de revisão sobre a relação entre todas as técnicas de inserção e as complicações relacionadas ao PICC em RNs não foram identificados na literatura e nas plataformas de registros desta revisão.

 Por esse motivo, este estudo teve como objetivo analisar a efetividade das técnicas de inserção de cateter central de inserção periférica na prevenção da ocorrência de complicações relacionadas a este dispositivo em recém-nascidos. Uma metanálise em rede foi utilizada para atingir este objetivo, uma vez que permite comparação entre três ou mais intervenções concorrentes simultaneamente em uma só análise, combinando-se evidência direta (estudos em que as intervenções de interesse são comparadas) e indireta (estudos em que as intervenções de interesse com um comparador comum são comparadas). A estimativas de efeito dos estudos de comparação direta e indireta são combinadas em uma rede de intervenções ^(^ ^17^ ^)^ . 

## Método

 Trata-se de uma revisão sistemática da literatura com metanálise pareada e em rede, reportada de acordo com as orientações do PRISMA ( *Preferred Reporting Items Systematic Reviews and Meta-Analysis Checklist* ) ^(^ ^18^ ^)^ e do PRISMA- *Network Meta-Analisys* (PRISMA-NMA) ^(^ ^19^ ^)^ . O protocolo foi registrado na plataforma PROSPERO, sob número CRD42022324152. 

 A estratégia PICOS ^(^ ^17^ ^)^ (acrônimo de *population, interventions, comparators, outcomes, study design* ) foi utilizada para delimitar a questão norteadora desta revisão: Em pacientes recém-nascidos (P), qual técnica de inserção do PICC (I) é mais efetiva na prevenção da ocorrência de complicações relacionadas ao uso deste dispositivo (O)? 

### Critérios de elegibilidade

Foram incluídos ensaios clínicos controlados aleatorizados e não aleatorizados que avaliaram técnicas de inserção de PICC em recém-nascidos hospitalizados e complicações relacionadas ao PICC referidas como medidas de resultado.

Excluíram-se os estudos que avaliaram as complicações relacionadas ao uso do PICC em RNs em atendimento ambulatorial; que avaliaram as complicações relacionadas ao uso do PICC em RNs em Unidades de Terapia Intensiva Mista (atendem neonatos junto com crianças); que avaliaram complicações relacionadas ao uso do PICC em crianças e adultos; que não descreveram as técnicas de inserção; que avaliaram diferentes tipos de cateteres PICC, sítios de inserção ou curativos; que avaliaram técnicas de inserção de outros tipos de dispositivo de acesso vascular central; estudos observacionais; estudos qualitativos; resumos de congressos, revisões, editoriais, opinião de especialistas, relato e série de casos, artigos de reflexão; e estudos sem grupo comparador.

### Fontes de informação

 A busca foi realizada nas seguintes bases eletrônicas de dados: *Cumulative Index to Nursing and Allied Health Literature* (CINAHL), Cochrane CENTRAL, Embase, Literatura Latino-Americana e do Caribe em Ciências da Saúde (LILACS), PubMed, Scopus e *Web of Science* . A literatura cinzenta foi consultada por meio das bases *Google Scholar* e *Open Grey.* A lista de referências dos estudos incluídos também foi consultada, a fim de identificar alguma publicação não identificada previamente nas buscas. Além disso, houve busca ativa de publicações que pudessem compor a amostra desta revisão por meio de consulta a especialistas, no dia. A busca foi realizada em 1º de janeiro de 2023. 

### Estratégia de busca

 Elaborou-se a estratégia de busca a partir da questão norteadora desta revisão, com uso de operador booleano “OR” entre sinônimos e semelhantes e “AND” entre palavras-chave. Inicialmente, a estratégia foi estruturada para a base de dados PubMed, utilizando-se dos descritores *Medical Subject Headings* (MeSH) e palavras-chave e, em seguida, adaptada para cada uma das bases pesquisadas. Não houve restrições de tempo ou idioma das publicações. 

A estratégia de busca elaborada na base de dados PubMed foi a seguinte: (“infant, newborn”[MeSH Terms] OR “Newborn Infant”[All Fields] OR “Newborn Infants”[All Fields] OR “Newborns”[All Fields] OR “Newborn”[All Fields] OR “Neonate”[All Fields] OR “Neonates”[All Fields] OR “infant, premature”[MeSH Terms] OR “Premature Infant”[All Fields] OR “Preterm Infants”[All Fields] OR “Preterm Infant”[All Fields] OR “Premature Infants”[All Fields] OR “Neonatal Prematurity”[All Fields] OR “Preterm”[All Fields] OR “infant, extremely premature”[MeSH Terms] OR “Extremely Premature”[All Fields] OR “Extremely Premature Infant”[All Fields] OR “Extremely Preterm Infants”[All Fields] OR “Extremely Preterm Infant”[All Fields] OR “Extremely Premature Infants”[All Fields] OR “infant, extremely low birth weight”[MeSH Terms] OR “Extremely Low Birth Weight Infant”[All Fields] OR “Extremely Low Birth Weight Infants”[All Fields] OR “infant, very low birth weight”[MeSH Terms] OR “very low birth weight infant”[All Fields] OR “very low birth weight infants”[All Fields] OR “very low birth weight infant”[All Fields] OR “very low birth weight infants”[All Fields] OR “Very Low Birth Weight”[All Fields] OR “infant, low birth weight”[MeSH Terms] OR “low birth weight infant”[All Fields] OR “low birth weight infants”[All Fields] OR “low birth weight infant”[All Fields] OR “low birth weight infants”[All Fields] OR “Low Birth Weight”[All Fields] OR “Low Birth Weights”[All Fields] OR “infant, small for gestational age”[MeSH Terms] OR “Small for Gestational Age”[All Fields] OR “Term Birth”[MeSH Terms] OR “Term Births”[All Fields] OR “Fullterm Birth”[All Fields] OR “Fullterm Births”[All Fields] OR “infant, postmature”[MeSH Terms] OR “Postmature Infant”[All Fields] OR “Postmature Infants”[All Fields] OR “Neonatal”[All Fields] OR “intensive care units, neonatal”[MeSH Terms] OR “Neonatal Intensive Care Unit”[All Fields] OR “Neonatal Intensive Care Units”[All Fields] OR “Neonatal Intensive Care”[All Fields] OR “Neonatal ICU”[All Fields] OR “Neonatal ICUs”[All Fields]) AND (“catheterization, peripheral”[MeSH Terms] OR “Peripherally Inserted Central Catheter”[All Fields] OR “Peripherally Inserted Central Catheters”[All Fields] OR “PICC”[All Fields] OR “PICCs”[All Fields] OR “Peripherally Inserted Central Catheter Line”[All Fields] OR “Peripherally Inserted Central Catheter Lines”[All Fields] OR “PICC Line”[All Fields] OR “PICC Lines”[All Fields]) AND (“Complications”[MeSH Subheading] OR “Complication”[All Fields] OR “Treatment Failure”[MeSH Terms] OR “Treatment Failures”[All Fields] OR “PICC Failure”[All Fields] OR “PICC Complication”[All Fields] OR “PICC Complications”[All Fields] OR “Infections”[All Fields] OR “Infection”[All Fields] OR “Catheter-Related Infections”[MeSH Terms] OR “catheter related infection”[All Fields] OR “catheter related infection”[All Fields] OR “Central Line-Associated Bloodstream Infection”[All Fields] OR “CLABSI”[All Fields] OR “Central Line-Associated Bloodstream Infections”[All Fields] OR “Bloodstream Infection”[All Fields] OR “Bloodstream Infections”[All Fields] OR “Catheter-Associated Bloodstream”[All Fields] OR “Catheter-Associated Bloodstream Infections”[All Fields] OR “Central Line Bloodstream Infection”[All Fields] OR “CABSI”[All Fields] OR “CRBSI”[All Fields] OR “Neonatal Nosocomial Infections”[All Fields] OR “Neonatal Nosocomial Infection”[All Fields] OR “Deep Vein Thrombosis”[All Fields] OR “Catheter-Related Deep Vein Thrombosis”[All Fields] OR “Thrombosis”[MeSH Terms] OR “Thromboses”[All Fields] OR “Thrombus”[All Fields] OR “Blood Clot”[All Fields] OR “Blood Clots”[All Fields] OR “Phlebitis”[MeSH Terms:noexp] OR “Phlebitides”[All Fields] OR “Periphlebitis”[All Fields] OR “Adverse Effects”[MeSH Subheading] OR “Adverse Effect”[All Fields] OR “Pericardial effusion”[All Fields] OR “Cardiac Tamponade”[All Fields] OR “Arterial Puncture”[All Fields] OR “Nerve Injury”[All Fields] OR “Nerve Injuries”[All Fields] OR “embolism, air”[MeSH Terms] OR “Air Embolism”[All Fields] OR “Air Embolisms”[All Fields] OR “arrhythmias, cardiac”[MeSH Terms] OR “Cardiac Dysrhythmia”[All Fields] OR “Cardiac Dysrhythmias”[All Fields] OR “Arrhythmia”[All Fields] OR “Arrythmia”[All Fields] OR “Cardiac Dysrhythmia”[All Fields] OR ((“central”[All Fields] OR “centrally”[All Fields] OR “centrals”[All Fields]) AND (“vascular access devices”[MeSH Terms] OR (“vascular”[All Fields] AND “access”[All Fields] AND “devices”[All Fields]) OR “vascular access devices”[All Fields] OR (“vascular”[All Fields] AND “access”[All Fields] AND “device”[All Fields]) OR “vascular access device”[All Fields]) AND (“malposition”[All Fields] OR “malpositioned”[All Fields] OR “malpositioning”[All Fields] OR “malpositionings”[All Fields] OR “malpositions”[All Fields])) OR “Infiltration”[All Fields] OR “Extravasation”[All Fields] OR “Extravasation of Diagnostic and Therapeutic Materials”[MeSH Terms]).

### Processo de seleção

 Após a remoção das duplicatas por meio do *software* *Endnote* *Web (Clarivate Analytics* ) ^(^ ^20^ ^)^ , a lista de referências dos estudos identificados nas bases de dados foi exportada para o *software on-line Rayyan* ( *Rayyan* , *Qatar Computing Research Institute* ) ^(^ ^21^ ^)^ . 

O processo de seleção dos estudos foi realizado em duas fases. Na primeira, dois revisores (L.O.B.; G.B.), de forma independente, procederam com a leitura de títulos e resumos dos estudos identificados nas bases de dados, aplicando os critérios de elegibilidade. Na Fase 2, os revisores (L.O.B.; G.B.) aplicaram novamente os critérios de inclusão após leitura na íntegra dos estudos. As divergências foram resolvidas por meio de discussão entre ambos e o terceiro revisor (A.S.M.) foi acionado apenas quando não chegaram a um consenso.

### Processo de coleta de dados

Os dois revisores (L.O.B.; G.B.) extraíram os seguintes dados dos estudos selecionados na fase 2: características do estudo (autor, ano de publicação, país, objetivos do estudo e principais conclusões) e da amostra (número de RNs e de cateteres; idade gestacional e peso de nascimento, idade gestacional corrigida ou dias de vida e peso no momento da inserção do PICC), técnica de inserção realizada e comparador, desfechos primários e secundários.

 Os dados coletados foram organizados em tabelas no *Microsoft Word* ^®^ , e os desfechos inseridos no programa *Microsoft Excel* ^®^ por um revisor, com verificação cruzada independente de outro. Posteriormente, os dados foram transportados para o *software* R versão 4.2.3. 

### Desfechos

 As taxas de complicações gerais e específicas relacionadas ao PICC foram consideradas como desfechos primários. A definição de complicações baseou-se nos termos descritos no conjunto de boas práticas da INS (2021), assim como no que este considera como complicações relacionadas à técnica de inserção: punção arterial, arritmias cardíacas, lesão de nervos, embolia aérea e mau posicionamento primário de dispositivo de acesso vascular central. As complicações relacionadas ao uso do cateter foram trombose venosa profunda associada ao cateter, mau posicionamento secundário (migração da ponta do cateter intra ou extravascular), flebite, infiltração e extravasamento, infecção da corrente sanguínea associada à linha central, oclusão, ruptura de cateter e lesão de pele associada ao cateter ^(^ ^11^ ^)^ . Estas últimas complicações que ocorrem durante a permanência do PICC foram acrescentadas por terem sido descritas como preveníveis a depender da técnica de inserção utilizada ^(^ ^11^ ^)^ . Além disso, como foram inclusos apenas estudos com alteração exclusivamente da técnica de inserção entre os grupos acompanhados, não havendo nenhuma diferença no material utilizado e nos cuidados e procedimentos de manutenção do PICC, pode-se dizer que estes desfechos analisados também pelos autores, nestes estudos, estariam relacionados somente às técnicas de inserção empregadas. As diferentes nomenclaturas utilizadas pelos autores dos estudos para definir as complicações foram normatizadas conforme a descrição proposta no conjunto de boas práticas da INS ^(^ ^11^ ^)^ . 

Os desfechos secundários coletados dos estudos que possuíam esses dados foram: taxa de sucesso geral (TSG) ou número de PICCs inseridos com sucesso em todas as tentativas de inserção; taxa de sucesso da primeira punção; número de punções; e tempo de permanência do cateter.

### Risco de viés dos estudos incluídos

 A avaliação do risco de viés dos ensaios clínicos aleatorizados incluídos foi realizada por dois revisores, utilizando a ferramenta *Risk of Bias* 2 (RoB2) ^(^ ^17^ ^)^ , desenvolvida pela Colaboração Cochrane *.* Já os ensaios clínicos não aleatorizados foram avaliados por meio ferramenta *Risk of Bias In Non-Randomised Studies of Interventions* (ROBINS-I), também desenvolvida pela Colaboração Cochrane ^(^ ^22^ ^)^ . 

### Medidas de efeito

 Os dados extraídos e os resultados encontrados foram expressos por meio de frequências relativas, absolutas ou por médias e desvios-padrão. Para desfechos binários, a medida de efeito utilizada foi o risco relativo (RR) e, para o desfecho contínuo, a diferença de médias/ *mean difference* (MD). Todos os resultados foram reportados com seus respectivos intervalos de confiança (IC) de 95%. As figuras e gráficos foram realizados pelo programa estatístico ( *Software* R *Statistics* ). 

### Métodos de síntese

 Os dados referentes aos desfechos foram transportados para o *Software* R *Statistics* , versão 4.2.3, utilizando os pacotes meta versão 6.2-1 e netmeta versão 2.8-1 ( *The R* *Foundation* , Vienna, Áustria). Para cada desfecho analisado, um diferente número de estudos comparou, diretamente, técnicas de inserção novas (IC-ECG, fórmula, etc) com a técnica de punção guiada por marcos de referência anatômicos (comparador comum). Uma metanálise de intervenção foi realizada com os estudos considerados homogêneos quanto ao delineamento do estudo, às características da amostra e à técnica de inserção realizada, em que se considerou nível de significância £ 0,05. O comparador comum presente em cada estudo permitiu comparação simultânea de todos os tratamentos de interesse (técnicas de inserção) por meio de uma metanálise em rede. As análises foram realizadas segundo a abordagem frequentista, utilizando o modelo de efeitos aleatórios, com o método de Mantel-Haenzel para desfechos binários e o método do inverso da variância para o desfecho contínuo. Avaliou-se a heterogeneidade de acordo com o I ^2^ , com base na classificação sugerida pelo PRISMA ^(^ ^18^ ^)^ . 

 Para ordenar os tratamentos avaliados em cada metanálise em rede em um *ranking* , estimou-se o P- *score* , que se baseia na estimativa ponto e no erro-padrão de cada estimativa incluída na metanálise de rede e mensura a magnitude da certeza de que um tratamento é melhor do que outro. Tal escore varia de 0 a 1, e valores próximos de 1 indicam superioridade do tratamento em avaliação ^(^ ^23^ ^)^ . 

 Os estudos realizados apenas com prematuros foram excluídos das metanálises em rede, por tratar-se de uma amostra peculiar e diferenciada das utilizadas nos estudos que combinam prematuros com RNs a termo, evitando-se, assim, a intransitividade ^(^ ^17^ ^)^ . Prematuros possuem anatomia e fisiologia da pele e do sistema vascular imaturos, imunidade mais baixa, maiores taxas de infecções e complicações mecânicas ^(^ ^4^ ^,^ ^24^ ^-^ ^28^ ^)^ e, ainda, maior chance de posicionamento adequado da ponta ^(^ ^29^ ^)^ . 

Ressalta-se que a metanálise em rede não havia sido prevista no protocolo desta revisão, mas considerando-se a viabilidade dessa análise e a importância/relevância da evidência que ela fornece, optou-se por conduzi-la. A decisão de realizá-la se deu a partir da análise dos dados dos estudos elegíveis, quando verificada a presença de um grupo-controle em comum entre as técnicas de inserção avaliadas nos estudos incluídos. Além disso, a metanálise em rede é o método que melhor responde à questão norteadora desta revisão.

### Avaliação do viés de publicação

 Uma busca na página *Clinicaltrials.gov* foi realizada por resultados não publicados de ensaios clínicos como forma de reduzir o risco de viés devido à falta de resultados em uma síntese ^(^ ^17^ ^)^ . Não foi possível gerar o *funnel plot* , por este recurso exigir o mínimo de 10 ensaios para garantir adequado poder de detecção de assimetrias ^(^ ^17^ ^)^ . 

### Avaliação da certeza da evidência

 A abordagem *Grading of Recomendations, Assessment, Development, and Evaluation Working Group* (GRADE) foi usada para avaliar a qualidade e certeza das evidências para cada desfecho de cada comparação ^(^ ^30^ ^)^ , com rebaixamento ou elevação em um ou dois pontos com base em critérios de julgamento. Utilizou-se a versão baseada na *web* do GRADEpro para apresentar as conclusões para os principais desfechos das comparações diretas (GRADEpro GDT; *the* GRADE *Working Group* , 2022). A avaliação da certeza da evidência das estimativas de efeito encontradas na metanálise em rede foi realizada em quatro etapas com auxílio de formulário próprio elaborado no *Microsoft Excel* ^®^: 1) apresentação das estimativas diretas e indiretas de cada comparação de interesse; 2) graduação da certeza da evidência de cada estimativa direta e indireta; 3) apresentação das estimativas de efeito da metanálise em rede da comparação de interesse; e 4) graduação da certeza da evidência das estimativas da metanálise em rede ^(^ ^31^ ^)^ . Nessas etapas, foram avaliadas a intransitividade das estimativas indiretas, a incoerência (ou inconsistência, como o PRISMA denomina) e a imprecisão da rede ^(^ ^32^ ^-^ ^34^ ^)^ . 

## Resultados

 Identificou-se um total de 2697 estudos nas bases de dados e literatura cinzenta, dos quais 1294 eram duplicados, o que resultou em 1403 artigos selecionados para leitura de títulos e resumos. Um fluxograma detalhado da seleção, exclusão e inclusão dos estudos, adaptado do PRISMA, está apresentado na Figura 1 . Cabe acrescentar que, dos 100 registros selecionados na literatura cinzenta, sete estavam duplicados; os demais foram triados em relação a título e resumo, mas nenhum foi incluído nesta revisão. 

**Figura 1:** - Fluxograma da busca na literatura e dos critérios de seleção adaptado do PRISMA ^(^ ^18^ ^)^

* CINAHL = *Cumulative Index to Nursing and Allied Health Literature;* ^†^ ILACS = Literatura Latino-Americana e do Caribe em Ciências da Saúde

 Oito estudos foram incluídos nesta revisão, com amostra total de 1126 RNs. Cinco foram originários da China ^(^ ^14^ ^,^ ^28^ ^,^ ^35^ ^-^ ^37^ ^)^ , um dos Estados Unidos ^(^ ^38^ ^)^ , um da Índia ^(^ ^39^ ^)^ e um do Brasil ^(^ ^40^ ^)^ . O período de publicação variou de 2013 a 2021, e um estudo estava disponível apenas no idioma chinês ^(^ ^35^ ^)^ . 

 Em relação ao delineamento do estudo, seis eram ensaios clínicos controlados aleatorizados ^(^ ^14^ ^,^ ^35^ ^-^ ^36^ ^,^ ^38^ ^-^ ^40^ ^)^ e dois ensaios não aleatorizados ^(^ ^28^ ^,^ ^37^ ^)^ . 

 As amostras variaram dentro da população de neonatos, sendo que três estudos avaliaram as técnicas de inserção apenas em RNs prematuros ^(^ ^28^ ^)^ e muito prematuros/prematuros extremos ^(^ ^38^ ^-^ ^39^ ^)^ e quatro as técnicas de inserção em RNs prematuros junto com a termo ^(^ ^14^ ^,^ ^35^ ^,^ ^37^ ^,^ ^40^ ^)^ . Um estudo citou apenas o peso médio de 2500g no dia da inserção ^(^ ^36^ ^)^ . 

 As técnicas de inserção do PICC em RNs encontradas nos estudos incluídos foram: eletrocardiograma intracavitário (IC-ECG) para guiar a ponta ^(^ ^14^ ^,^ ^35^ ^)^ ; eletrocardiograma intracavitário com ultrassonografia para visualização vascular (IC-ECG/USG) ^(^ ^28^ ^,^ ^37^ ^)^ ; ultrassonografia em tempo real (USGRT) para visualização da ponta durante a inserção e guiando esta ^(^ ^38^ ^-^ ^39^ ^)^ ; marco de referência anatômico (MRA) modificado, em que a intervenção ocorreu com a mensuração de tamanho do cateter a ser inserido com um referencial anatômico diferente ^(^ ^40^ ^)^ ; uso de fórmula, em que uma equação determina a quantidade de cateter que deve ser inserida associada à realização de compressa morna por 15 minutos no sítio de inserção antes da punção ^(^ ^36^ ^)^ ; e punção guiada por marcos de referência anatômicos, caracterizada por punção cega (sem auxílio de tecnologias), mensuração por MRA (quantidade de centímetros de cateter a ser inserido é predeterminada por uma mensuração realizada na superfície do corpo do provável sítio de inserção até a veia cava superior ou inferior) e visualização da ponta por raio x ^(^ ^14^ ^,^ ^28^ ^,^ ^35^ ^-^ ^40^ ^)^ , que foi o grupo-controle de todos os estudos. Ou seja, o comparador/controle em todos os estudos foi uma mesma técnica, o que levou a rede a uma geometria em formato de estrela. Isso quer dizer que todas as técnicas novas foram comparadas com um comparador comum, sem comparação direta entre elas, mas sim, indiretas. 

 O risco de viés dos estudos incluídos encontra-se detalhado na Figura 2 . 

**Figura 2:** - Avaliação do risco de viés aplicada aos estudos por meio da ferramenta RoB2 ( *Risk of Bias* 2) acima e ROBINS-I ( *Risk of Bias In Non-randomised Studies of Interventions)* abaixo. Brasília, DF, Brasil, 2023

 Os ensaios aleatorizados mostraram algumas preocupações em relação ao processo de aleatorização por algumas diferenças nas características dos participantes ^(^ ^38^ ^)^ , falta de informações detalhadas no artigo ^(^ ^36^ ^)^ ou alocação 1:1 ^(^ ^14^ ^)^ . Não era possível que os profissionais que aplicaram a intervenção estivessem cegos, pois precisariam saber qual era a intervenção para aplicá-la, com exceção de um estudo ^(^ ^40^ ^)^ . Em pesquisa que utilizou USGRT ^(^ ^38^ ^)^ , foram observados alguns desvios das intervenções pretendidas que podem ter afetado os desfechos, com falhas de inserção não explicadas e podendo estar diretamente relacionadas com a intervenção, alterando, inclusive, o número de participantes nos dois grupos. Apesar de não ter sido identificado o protocolo do estudo de uma das publicações ^(^ ^39^ ^)^ , o que levou o domínio de viés dos resultados reportados a ser julgado como “algumas preocupações”, este estudo foi avaliado como baixo risco de viés por ser bem desenhado e conter a afirmação dos autores de que todas as intervenções e análises foram realizadas de acordo com o planejado antes do início do estudo. Duas publicações não informaram se os responsáveis pela avaliação dos desfechos sabiam ou não qual grupo estavam avaliando ^(^ ^35^ ^-^ ^36^ ^)^ . 

 Os ensaios não aleatorizados obtiveram as informações do seu grupo-controle por meio de dados retrospectivos, e os artigos possuíam algumas perguntas dos domínios respondidas como “não informado” ^(^ ^28^ ^,^ ^37^ ^)^ . Por exemplo, não havia informação se os profissionais que avaliaram os desfechos sabiam a qual grupo os participantes pertenciam, o que poderia ter influenciado, principalmente, a mensuração do desfecho mau posicionamento primário da ponta, única complicação medida no segundo estudo ^(^ ^37^ ^)^ . Este mesmo estudo ^(^ ^37^ ^)^ foi considerado com crítico risco de viés por haver diferenças nas características e no número dos participantes entre os grupos com nenhum tipo de controle dos fatores de confusão medidos, além de os participantes parecerem ter tido seguimentos diferenciados. 

 As características dos estudos primários incluídos na revisão e seus resultados estão descritos na Figura 3 . 

**Figura 3:** - Características dos estudos incluídos e resultados individuais encontrados (n=8). Brasília, DF, Brasil, 2023

**Características do estudo e da amostra**					**Resultados**	**Principais Conclusões**
**Autor; Ano; País**	**Intervenções (Técnica de inserção e Controle)**	**Amostra (n)**	IGN ^*^ (s ^†^ **) e PN** ^§^ **(g** ^||^ **)**	**IGC** ^‡^ **(s** ^†^ **) ou dias de vida e/ou peso (g** ^||^ **)**	**Complicações** **n (%) experimental x n (%) controle**	
Katheria, et al.; 2013 ^(^ ^38^ ^)^ ; Estados Unidos	Grupo Experimental: punção cega, mensuração por marco de referência anatômico, USGRT ^**^ para verificação da ponta	20		IGC ^‡^ 31± 4s ^†^ Peso:1185 ± 538g ^||^ 4 ±5 dias	Malpos. 1 ^o¶^: 5(25%) **x** 16(57,1%)	USGRT ^**^ reduziu o tempo do procedimento de inserção em 40% ( *p* =0,034), a necessidade de raio x ( *p* =0,001) e de manipulações ( *p* =0,034).
	Grupo-Controle: punção guiada por marcos de referência anatômicos	28		IGC ^‡^ 30± 4s ^†^ Peso:1208 ± 481g ^||^ 5 ±5 dias	Malpos. 2 ^o††^ (Tamponamento cardíaco): 1 (5%) **x** 0 (0%)	
Ling, et al.; 2019 ^(^ ^14^ ^)^ ; China	Grupo Experimental: punção cega, mensuração por marco de referência anatômico, IC-ECG ^‡‡^ para guiar a ponta	80	IGN ^*^ 37,1± 1,4s ^†^ (32-40)	3,4 ± 0,5 dias (1-6)	Complicações gerais: 3(3,75%) **x** 19(23,75%), *p* =0,000 Arritmia: 0(0%) **x** 4(5%), *p* =0,043 Flebite: 1(1,25%) **x** 7(8,75%), *p* =0,03 Infecção: 0(0%) **x** 1(1,25%), *p* =0,316 Lesão de pele: 1(1,25%) **x** 1(1,25%), *p* =0,316 Malpos. 1 ^o¶^: 4(5%) **x** 17(21,25%), *p* =0,002 Oclusão: 1(1,25%) **x** 4(5%), *p* =0,173	IC-ECG ^‡‡^ melhorou taxa de primeira tentativa de inserção do PICC ^§§^ até posição central, demandou menos tempo e custo médico, menor exposição à radiação e reduziu taxas de complicações.
	Grupo-Controle: punção guiada por marcos de referência anatômicos	80	IGN ^*^ 36,8 ± 1,3s ^†^ (32-40)	3,2 ± 0,3 dias (1-5)		
Oleti, et al.; 2019 ^(^ ^39^ ^)^ ; Índia	Grupo Experimental: punção cega, mensuração por marco de referência anatômico, USGRT ^**^ para verificação da ponta	40	IGN ^*^ 31,1± 3,1s ^†^ PN ^§^ 1286g ^||^ (926-1662)	1,75 dias (0,83-5,37)	Infecção: 4(10,2%) **x** 1(2,5%) - RR ^||||^ 4,1; IC ^¶¶^ 95% 0,47-35, *p* =0,24 Infiltração ou oclusão: 4(10%) **x** 6(15%) Malpos. 1 ^o¶^: 13(32,5%) **x** 27(67,5%) – RR ^||||^ 0,48; IC ^¶¶^ 95%: 0,29-0,79; *p* =0,002	O uso da USGRT ^**^ reduziu a incidência de mau posicionamento da ponta em 52%, podendo ser recomendada para a inserção do PICC ^§§^ .
	Grupo-Controle: punção guiada por marcos de referência anatômicos	40	IGN ^*^ 31,4 ±3,6s ^†^ PN ^§^ 1061g ^||^ (889-1636)	1,04 dias (0,77-4,87)		
Tang, et al.; 2021 ^(^ ^35^ ^)^ ; China	Grupo Experimental: punção cega, mensuração por marco de referência anatômico, IC-ECG ^‡‡^ para guiar a ponta	105	IGN ^*^ 36,9 ±1,5s ^†^ (33-40)		Complicações gerais: 4(3,8%) **x** 23(21,9%); *p* =0,000 Arritmia: 0(0%) **x** 6(5,7%); *p* =0,015 Flebite: 2(1,9%) **x** 9(8,6%); *p* =0,029 Infecção: 0(0%) **x** 1(1%); *p* =1 Lesão de pele: 1(1%) **x** 3(2,9%); *p* =0,311 Trombose: 1(1%) **x** 4(3,8%); *p* =0,184	O uso de IC-ECG ^‡‡^ ajuda a detectar e corrigir oportunamente ponta ectópica e a lesão vascular resultante, infecção e arritmia, trazendo maior segurança do que o controle.
	Grupo-Controle: punção guiada por marcos de referência anatômicos	105	IGN ^*^ 36,4 ±1,6s ^†^ (32-40)			
Tomazoni, et al.; 2022 ^(^ ^40^ ^)^ ; Brasil	Grupo Experimental: punção cega, mensuração por marco de referência anatômico modificado (sítio de inserção até junção esternoclavicular direita), raio x para verificação da ponta	44	IGN ^*^ e PN ^§^ estão estratifica-dos em faixas		Complicações gerais: 17(38,6%) **x** 27(61,3%) Flebite: 1(2,3%) **x** 3(6,8%) Infiltração: 3(6,8%) **x** 6(13,6%) Malpos. 1 ^o^ ^¶^: 23(52,27%) **x** 43(97,7%); *p* =0,000 Malpos. 2 ^o††^: 1(2,3%) **x** 1(2,3%) Oclusão: 3(6,8%) **x** 4(9,1%) Ruptura: 1(2,3%) **x** 1(2,3%) Suspeita de infecção: 2(4,5%) x 1(2,3%)	O uso do método de mensuração modificada proporcionou melhores resultados para o posicionamento adequado da ponta do PICC ^§§^ .
	Grupo-Controle: punção guiada por marcos de referência anatômicos (sítio de inserção até junção esternoclavicular direita e até 3º espaço intercostal)	44				
Wu, et al.; 2021 ^(^ ^36^ ^)^ ; China	Grupo Experimental: punção em safena após 15 minutos de compressa morna, guiada por fórmula, raio x para verificação da ponta *Fórmula >1500g*: L ^***^ = M ^†††^ - 1,5 +23 0,3 *Fórmula <1500g*: L ^***^ = 23 – 1,5 - M ^†††^ 0,3 L ^***^: tamanho do PICC ^§§^ a ser inserido M ^†††^: peso do recém-nascido Grupo-Controle: punção em veia safena guiada por marcos de referência anatômicos	65		16,52± 0,63 dias (1,47-26,46) Peso 2510± 180g ^||^ (1170-3760)	Complicações gerais: 4(6,15%) **x** 23(35,38%); *p* <0,001 Flebite: 1(1,53%) **x** 7(10,76%) Infecção: 1(1,53%) **x** 6(9,23%) Malpos. 2 ^o††^: 1(1,53%) **x** 4(6,15%) Trombose: 1(1,53%) **x** 6(9,23%)	O uso da fórmula para determinar a quantidade de cateter a ser inserido reduziu tempo de internação, escala de dor e complicações relacionadas, bem como aumentou a taxa de sucesso da primeira punção e o tempo de permanência do cateter.
		65		16,73 ± 0,84 dias (8,4 -26,81) Peso 2570 ± 210g ^||^ (1190-3890)	Obs: O Malpos. 1 ^o¶^ não foi verificado por indivíduo, mas quanto ao número de centímetros que tiveram que ser tracionados: Fórmula com tração de 0,25 ± 0,08 centímetros **x** Controle com tração de 2,87 ± 0,23 centímetros ( *p* < 0,000)	
Xiao, et al.; 2019 ^(^ ^28^ ^)^ ; China	Grupo Experimental: USG ^‡‡‡^ para visualização vascular e IC-ECG ^‡‡^ para guiar a ponta	78	IGN ^*^ 32,17 ±2,63s ^†^ (28-37) PN ^§^ 1520± 377,38g ^||^	15,21 ±7,52 dias Peso 1657,44 ± 307,22g ^||^	Complicações gerais: 5(6,41%) **x** 14(16,86%); *p* =0,040 Flebite: 2(2,56%) **x** 7(10,84%); *p* =0,202 Infecção: 1(1,28%) **x** 3(3,61%); *p* =0,657 Malpos. 1 ^o¶^: 5(6,41%) **x** 22(26,51%); *p* =0,001 Ruptura: 2(2,56%) **x** 4(4,82%); *p* =0,735	IC-ECG ^‡‡^ pode contribuir para diminuir as taxas de reposicionamen-to da ponta e as complicações, além de aumentar taxas de posicionamento adequado de ponta na primeira tentativa.
	Grupo-Controle: punção guiada por marcos de referência anatômicos	83	IGN ^*^ 32,36 ±2,78s ^†^ (28-37) PN ^§^ 1508,13± 279,31g ^||^	13,19 ±8,8 dias Peso 1571,63 ± 266,16g ^||^		
Zhou et al.; 2017 ^(^ ^37^ ^)^ ; China	Grupo Experimental: USG ^‡‡‡^ para visualização vascular e IC-ECG ^‡‡^ para guiar a ponta	49	IGN ^*^ 35 ± 4s ^†^ (28-41)	17 ± 16 dias (1-28) PN 2700 ± 900g ^||^ (1100-4900)	Malpos. 1 ^o¶^: 3(6,12%) **x** 75(37,5%); *p* <0,001 Malpos. 2 ^o^ ^††^ (Efusão pleural): 0(0%) **x** 1(0,5%)	IC-ECG ^‡‡^ com coluna salina pode ser aplicada a neonatos, guiando a ponta do cateter adequada-mente, diminuindo, assim, os riscos, atrasos e custos de ajustes da ponta.
	Grupo-Controle: punção guiada por marcos de referência anatômicos	200	IGN ^*^ 36 ± 3s ^†^ (28-41)	13 ± 12 dias (1-28) PN 2700 ± 900g ^||^ (1000-5000)		

^‡^ IGC = Idade gestacional corrigida;

^**^ USGRT = Ultrassonografia em tempo real;

^||||^ RR = Risco relativo;

^¶¶^ IC = Intervalo de confiança;

^¶^ Malpos.1º = Mau posicionamento primário da ponta;

^***^ L = Tamanho do PICC a ser inserido;

^†††^ M = Peso do recém-nascido;

^§§^ PICC = Cateter central de inserção periférica;

^§^ PN = Peso de nascimento;

^||^ g = Gramas;

^‡‡‡^ USG = Ultrassonografia

^‡‡^ IC-ECG = Eletrocardiograma intracavitário;

^†^ s = Semanas;

^††^ Malpos.2º = Mau posicionamento secundário intravascular ou extravascular da ponta;

^*^ IGN = Idade gestacional de nascimento;

 Nem todos os desfechos foram avaliados em todos os estudos incluídos. Aqueles avaliados em mais de um estudo incluído, com amostra semelhante e mais de uma técnica de inserção, foram analisados na rede. Na estrutura ou geometria de cada rede, o grupo-controle foi o único comparado diretamente com as demais técnicas de inserção e estas foram comparadas indiretamente entre si, exatamente por possuírem este comparador comum. Assim, para todos os desfechos em que foi possível a metanálise em rede, a geometria desta foi a mesma. A estrutura da rede de todos os desfechos que permitiram a metanálise em rede é representada graficamente na Figura 4 . Ressalta-se que, na geometria da rede, círculos representam as intervenções, cujos tamanhos correspondem ao número de cada amostra, e linhas retas são as comparações diretas entre as técnicas de inserção, com maior espessura quão maior o número de estudos que realizaram aquela comparação. 

**Figura 4:** - Geometria da rede de todos os desfechos que permitiram a metanálise em rede. Brasília, DF, Brasil, 2023

*IC-ECG = Eletrocardiograma intracavitário; ^†^ MRA = Marco de referência anatômico; ^‡^ IC-ECG/USG = Eletrocardiograma intracavitário e ultrassonografia para visualização vascular

 Em relação aos desfechos primários, um deles foi complicações gerais, que correspondeu ao total de complicações relatado por cada estudo, podendo ser referente a duas ou mais complicações, a depender do objetivo da pesquisa e do tempo de acompanhamento do RN. Dois estudos não verificaram este desfecho ^(^ ^37^ ^-^ ^38^ ^)^ , pois foram os únicos que acompanharam suas amostras apenas até a confirmação da localização da ponta e não até a retirada do PICC (vide tempo de permanência do dispositivo), como nos outros. O mau posicionamento primário da ponta não foi computado dentro do desfecho complicações gerais como as demais complicações por nenhum autor dos estudos incluídos. A Figura 5 mostra os resultados relacionados às complicações gerais no que se refere à metanálise com as comparações diretas e indiretas. 

**Figura 5:** - Gráficos de floresta que demonstram o efeito das técnicas de inserção no desfecho complicações gerais, tanto das comparações diretas (acima) como das realizadas na rede (abaixo). Brasília, DF, Brasil, 2023

*IC-ECG = Eletrocardiograma intracavitário; ^†^ RR = Risco relativo; ‡CI = Intervalo de confiança; §MRA = Marco de referência anatômico; ^||^ USGRT = Ultrassonografia em tempo real; ^¶^ IC-ECG/USG = Eletrocardiograma intracavitário e ultrassonografia para visualização vascular

 A complicação flebite foi analisada com metanálise de intervenção e em rede com os estudos com mesma amostra. Na de intervenção com os dois estudos que utilizaram IC-ECG na inserção do PICC ^(^ ^14^ ^,^ ^35^ ^)^ , constatou-se a possibilidade de tal técnica reduzir significativamente o risco de flebite em até 81% (RR 0,19; IC 95%: 0,06-0,65). Na metanálise em rede, o IC-ECG, quando comparado com o controle, permaneceu como a única técnica de inserção capaz de diminuir esta complicação de forma significativa. 

 O mau posicionamento primário da ponta do PICC foi um desfecho não mensurado diretamente em dois estudos ^(^ ^35^ ^-^ ^36^ ^)^ . Todas as intervenções (IC-ECG, IC-ECG/USG, USGRT e MRA modificado) reduziram o risco de mau posicionamento primário da ponta, com estimativa de grande magnitude, quando comparadas com o controle. Uma metanálise de intervenção foi realizada com dois estudos que compararam a USGRT com o grupo-controle ^(^ ^38^ ^-^ ^39^ ^)^ , com RR 0,47 (IC 95%: 0,31-0,72). Contudo, como esses ensaios ^(^ ^38^ ^-^ ^39^ ^)^ foram realizados com RNs muito prematuros e prematuros extremos, não foi possível incluí-los na metanálise em rede realizada com as demais técnicas de inserção. Nesta rede, também foi verificado que o IC-ECG/USG reduziu o risco de mau posicionamento primário quando comparado com MRA modificado (RR 0,31; IC 95% 0,10-0,96). 

 Assim, as técnicas de inserção reduziram significativamente complicações gerais, flebite e mau posicionamento primário da ponta. O *ranking* realizado de acordo com os resultados do P- *score* das técnicas mais efetivas na redução dessas complicações encontra-se na Figura 6 . Nesta figura, riscos relativos (RRs) menores que 1 favorecem a intervenção definida na coluna para os resultados da metanálise de rede (triângulo inferior) e a intervenção definida na linha para os resultados de metanálise pareados (triângulo superior). Dessa forma, verifica-se que as técnicas de inserção mais efetivas na redução das complicações gerais, flebite e mau posicionamento primário da ponta foram, respectivamente, IC-ECG (P- *score* 0,8399), fórmula (P- *score* 0,7562) e IC-ECG/USG (P- *score* 0,8863). 

**Figura 6:** - Tabelas de liga *(league tables)* demonstrando o *ranking* das técnicas de inserção mais efetivas na redução das complicações gerais, flebite e mau posicionamento primário da ponta com os respectivos riscos relativos resultados das comparações. Brasília, DF, Brasil, 2023

*IC-ECG = Eletrocardiograma intracavitário; ^†^ MRA = Marco de referência anatômico; ^‡^ IC-ECG/USG = Ultrassonografia para visualização vascular e ponta guiada por eletrocardiograma intracavitário

 A arritmia foi averiguada por meio de uma metanálise de intervenção realizada com os únicos dois estudos ^(^ ^14^ ^,^ ^35^ ^)^ que mensuraram este desfecho, verificando-se redução de seu risco com uso da técnica de inserção IC-ECG (RR 0,09; IC 95%: 0,01-0,71). 

 Com os desfechos infecção, mau posicionamento secundário da ponta e trombose venosa profunda associada ao cateter, realizou-se uma metanálise de intervenção e outra em rede, sendo constatada ausência de significância estatística nas estimativas de efeito pareadas e da rede. Um *ranking* das técnicas de inserção mais efetivas na tendência de redução das complicações citadas foi realizado e, neste, a fórmula foi considerada a melhor. 

 Duas complicações citadas no manual de 2021 da INS não foram encontradas em nenhum estudo incluído nesta revisão: a punção arterial inadvertida e a embolia aérea ^(^ ^11^ ^)^ . Outras duas complicações foram citadas nos estudos incluídos: oclusão e lesão de pele associada ao cateter. As metanálises realizadas para estes dois últimos desfechos (em rede para oclusão e de intervenção para lesão de pele) não mostraram redução significativa a depender da técnica de inserção utilizada. 

 Quanto aos desfechos secundários, a taxa de sucesso geral (TSG) foi acima de 80% em um estudo que utilizou USGRT (92,5% x 85%) ^(^ ^39^ ^)^ . Uma metanálise de intervenção foi realizada com este desfecho com apenas dois estudos homogêneos que utilizaram USGRT ^(^ ^38^ ^-^ ^39^ ^)^ em seu grupo experimental, resultando em RR 0,90; IC 95%: 0,60-1,36. 

 O estudo com maiores taxas de sucesso na primeira punção foi o que utilizou a fórmula como técnica de inserção (96,92% x 72,31%) ^(^ ^36^ ^)^ , enquanto com o MRA modificado a taxa chegou a 37,5% ^(^ ^40^ ^)^ . 

 Em relação ao tempo de permanência do cateter, a fórmula ^(^ ^36^ ^)^ e a USGRT ^(^ ^39^ ^)^ mostraram que o grupo experimental obteve, significantemente, maior tempo de permanência na comparação com o controle (MD 2,35; IC 95%: 2,07-2,63 e MD 0,9; IC 95%: 0,60-1,10, respectivamente). Já a técnica de inserção IC-ECG reduziu a média do tempo de permanência (MD -0,23; IC 95%: -1,21-0,75). 

 A certeza do corpo de evidências foi realizada por meio do GRADE. Nenhum desfecho avaliado apresentou certeza da evidência “alta”. As comparações diretas e indiretas foram avaliadas para cada desfecho, de modo que um resultado quanto à certeza da evidência das metanálises em rede fosse construído a partir da avaliação da incoerência e da imprecisão destas redes. A inconsistência na rede, como denomina o GRADE, é inexistente, porque, para cada desfecho, havia ou comparações diretas (técnicas x controle) ou indiretas (entre técnicas na rede), mas nunca uma combinação de comparação direta e indireta. Os resultados da avaliação estão presentes na Figura 7 . 

**Figura 7:** - Resultados das avaliações da certeza das evidências das estimativas diretas, indiretas e da metanálise em rede, de acordo com o *Grading of Recomendations, Assessment, Development, and Evaluation Working Group* (GRADE) realizado nos desfechos (n=11) com suas respectivas comparações. Brasília, DF, Brasil, 2023

**Desfecho**	**Comparações**	**Certeza da evidência das estimativas diretas**	**Certeza da evidência das estimativas indiretas**	**Certeza da evidência da metanálise em rede**
Arritmia	IC-ECG ^*^ x Controle	Moderada	-	-
Complicações gerais	IC-ECG ^*^ x Controle Fórmula x Controle MRA ^†^ modificado x Controle IC-ECG/USG ^‡^ x Controle USGRT ^§^ x Controle IC-ECG ^*^ x Fórmula IC-ECG ^*^ x MRA ^†^ modificado Fórmula x MRA ^†^ modificado	Moderada Moderada Moderada Baixa Muito baixa - - -	- - - - - Moderada Moderada Moderada	Moderada Baixa Baixa - - Baixa Moderada Baixa
Flebite	IC-ECG ^*^ x Controle MRA ^†^ modificado x Controle Fórmula x Controle Fórmula x MRA ^†^ modificado Fórmula x IC-ECG ^*^ IC-ECG ^*^ x MRA ^†^ modificado	Moderada Moderada Moderada - - -	- - - Moderada Moderada Moderada	Moderada Muito Baixa Baixa Muito Baixa Muito Baixa Muito Baixa
Infecção	IC-ECG ^*^ x Controle MRA ^†^ modificado x Controle Fórmula x Controle USGRT ^§^ x Controle Fórmula x MRA ^†^ modificado Fórmula x IC-ECG ^*^ IC-ECG ^*^ x MRA ^†^ modificado IC-ECG/USG ^‡^ x Controle	Moderada Baixa Moderada Muito baixa - - - Muito baixa	- - - - Baixa Moderada Baixa -	Muito baixa Muito baixa Baixa - Muito baixa Muito baixa Muito baixa -
Lesão de pele	IC-ECG ^*^ x Controle	Muito baixa	-	-
Mau posicionamento primário	IC-ECG ^*^ x Controle MRA ^†^ modificado x Controle IC-ECG/USG ^‡^ x Controle USGRT ^§^ x Controle IC-ECG/USG ^‡^ x MRA ^†^ modificado IC-ECG/USG ^‡^ x IC-ECG ^*^ IC-ECG ^*^ x MRA ^†^ modificado	Moderada Moderada Muito baixa Moderada - - -	- - - - Baixa Baixa Moderada	Moderada Moderada Baixa - Baixa Muito baixa Muito baixa
Mau posicionamento secundário	MRA ^†^ Modificado x Controle Fórmula x Controle IC-ECG/USG ^‡^ x Controle USGRT ^§^ x Controle Fórmula x IC-ECG/USG ^‡^ Fórmula x MRA ^†^ modificado MRA ^†^ modificado x IC-ECG/USG‡	Moderada Baixa Muito baixa Muito baixa - - -	- - - - Muito baixa Baixa Muito baixa	Muito baixa Muito baixa Muito baixa - Muito baixa Muito baixa Muito baixa
Oclusão	IC-ECG ^*^ x Controle MRA ^†^ modificado x Controle IC-ECG ^*^ x MRA ^†^ modificado	Moderada Moderada -	- - Moderada	Muito baixa Muito baixa Muito baixa
Tempo de permanência	IC-ECG ^*^ x Controle Fórmula x Controle USGRT ^§^ x Controle IC-ECG ^*^ x Fórmula	Moderada Moderada Moderada -	- - - Moderada	Muito baixa Moderada - Moderada
Trombose	IC-ECG ^*^ x Controle Fórmula x Controle Fórmula x IC-ECG ^*^	Alta Moderada -	- - Moderada	Baixa Muito baixa Muito baixa
Taxa de sucesso geral	MRA ^†^ modificado x Controle USGRT ^§^ x Controle	Baixa Muito baixa	- -	- -

^‡^ IC-ECG/USG = Eletrocardiograma intracavitário com ultrassonografia para visualização;

^*^ IC-ECG = Eletrocardiograma intracavitário;

^†^ MRA = Marco de referência anatômico;

^§^ USGRT = Ultrassonografia em tempo real

## Discussão

Esta revisão é a primeira a verificar a efetividade das diversas técnicas de inserção de PICC na prevenção da ocorrência de complicações em RNs. Com a síntese, avaliação e combinação criteriosa entre os oito estudos incluídos por meio de metanálises pareadas e em rede, foi possível verificar qual técnica de inserção, dentre as identificadas, é mais efetiva para diminuir determinadas complicações associadas ao dispositivo de acesso vascular central PICC. Dessa forma, houve significativa redução do risco de complicações gerais, arritmias, flebite, mau posicionamento primário da ponta do PICC, assim como alteração do tempo de permanência do cateter a depender da técnica de inserção empregada. Infecção, infiltração/extravasamento, mau posicionamento secundário da ponta, ruptura do cateter, trombose, oclusão, lesão de pele e TSG foram influenciados pelas técnicas de inserção e a maioria com redução absoluta, mas sem significância estatística e apresentando estimativas de efeito com muito baixa certeza da evidência.

 Prevenir complicações pode reduzir a morbidade, a necessidade de procedimentos adicionais e os custos ^(^ ^41^ ^)^ . Boas práticas durante a inserção do PICC em RNs podem mitigar exposição desnecessária do bebê, múltiplas punções venosas e risco de posicionamento inadequado do cateter, além de melhorar taxa de sucesso de inserção ^(^ ^42^ ^-^ ^43^ ^)^ . 

Nesta revisão, o uso de IC-ECG esteve associado a um risco significativamente menor e mais efetivo de complicações gerais, flebite e mau posicionamento primário da ponta e a uma redução das arritmias cardíacas, todas com moderada certeza da evidência. Também foi a melhor técnica para reduzir o tempo de permanência do cateter, especialmente quando comparado com a técnica que utiliza a fórmula. Com essa moderada certeza dos achados, pode-se dizer que o IC-ECG é uma técnica de inserção que deve ser utilizada na prática clínica por sua efetividade na prevenção de complicações importantes.

 Revisão sistemática e metanálise chinesa realizada com ensaios clínicos aleatorizados locais constatou que o IC-ECG reduziu o risco de flebite ( *Odds Ratio* – OR 0,33 - IC 95% 0,19-0,56, *p* <0,001) e o total de complicações (OR 0,23 - IC 95% 0,16-0,33, *p* <0,001), além de ter aumentado a acurácia da localização ótima da ponta (OR 5,37 - IC 95% 3,80-7,59, *p* <0,001) ^(^ ^44^ ^)^ . Esta metanálise corrobora os resultados aqui apresentados, podendo-se constatar efetividade do IC-ECG na redução de arritmias, de complicações gerais, de flebite, além do mau posicionamento primário da ponta e de suas possíveis consequências. Isto porque pesquisas e conjuntos de boas práticas associaram o aumento das taxas de complicações com a ponta do PICC não central, especialmente oclusões, extravasamento, outras complicações mecânicas, flebite e falha/remoção não eletiva ^(^ ^2^ ^,^ ^11^ ^,^ ^29^ ^,^ ^41^ ^,^ ^45^ ^-^ ^46^ ^)^ . A taxa de complicação de PICCs não centrais chega a ser 200% maior quando comparada aos centrais ^(^ ^29^ ^)^ . Ou seja, uma técnica de inserção capaz de prevenir o mau posicionamento primário do dispositivo de acesso vascular central diminuirá a ocorrência dessas outras complicações supracitadas, que podem ser extremamente deletérias. 

 A flebite é influenciada pela técnica de inserção do PICC, uma vez que pode ser causada quando a ponta não está central ^(^ ^2^ ^,^ ^35^ ^,^ ^47^ ^)^ (vasos de menor calibre estão mais sujeitos à irritação química e fricção constante do cateter). Pode, também, estar associada a outros fatores como cateter muito rígido, técnica de punção inadequada, sítio de punção inapropriado por movimentação constante do membro (especialmente membros superiores), infecção e incompatibilidade medicamentosa ^(^ ^11^ ^,^ ^35^ ^,^ ^48^ ^-^ ^49^ ^)^ . Dessa forma, como a técnica de inserção com o IC-ECG diminuiu mais efetivamente o mau posicionamento da ponta, não é surpresa ela ser também a melhor em reduzir o risco de flebite. 

 Em relação ao tempo de permanência, pode-se afirmar que o IC-ECG não foi efetivo em reduzi-lo quando comparado com o controle, mas o diminuiu de forma significativa quando comparado com a fórmula. Esse resultado é diferente do encontrado na revisão sistemática e metanálise chinesa, em que o uso de IC-ECG durante inserção reduziu este desfecho ao ser comparado com o controle (MD = 5,86, IC 95% 16,36-4,65, *p* =0,00) ^(^ ^44^ ^)^ . Destaca-se que o tempo de permanência do cateter deve ser discutido apenas se acrescentados outros dados que não foram colhidos e não estavam presentes nos estudos incluídos nesta revisão. Isso porque a interpretação da diminuição do TP pode tanto ser considerada positiva, se isto ocorreu por redução de complicações e consequente término da terapia intravenosa mais rapidamente, por exemplo, ou como negativa, se isso ocorreu por mais remoções precoces do cateter. 

 A técnica de inserção que utiliza uma fórmula baseada no peso do RN para predizer o tamanho, em centímetros, do cateter a ser inserido ( Figura 3 ) mostrou-se superior às demais ao aumentar significativamente o tempo de permanência (moderada certeza da evidência) e reduzir o risco de flebite (baixa confiança no efeito). Esta baixa certeza da evidência em relação à flebite leva à consideração de que a técnica mais efetiva na redução do seu risco foi, realmente, o IC-ECG, no qual a confiança é moderada, com estimativas mais precisas. Ademais, foi a segunda melhor técnica na diminuição do risco de complicações gerais e a com melhor taxa de sucesso na primeira tentativa de punção, menor necessidade de ajustes do PICC, de tempo de hospitalização e dor ^(^ ^36^ ^)^ . 

 Outras pesquisas com RNs também resultaram em fórmulas que predizem o tamanho de cateter a ser inserido, baseadas nas características físicas dos bebês como peso, altura e idade gestacional, garantindo uma forma mais acurada de localização central da ponta do PICC ^(^ ^25^ ^,^ ^50^ ^)^ . 

 No estudo com fórmula, a veia safena foi a puncionada, quase sempre na primeira tentativa e por enfermeiros treinados e com experiência ^(^ ^36^ ^)^ . A experiência de um time de enfermagem vascular é importante para o sucesso na inserção e segurança no que diz respeito às taxas de complicações ^(^ ^28^ ^,^ ^51^ ^)^ . 

 Destaca-se que, além da compressa morna utilizada na pesquisa com a fórmula ^(^ ^36^ ^)^ , outras investigações testaram estratégias distintas para manter o RN aquecido e veias vasodilatadas durante a inserção do PICC, tais como uso de manta térmica ^(^ ^52^ ^)^ e colocação do RN em berço com calor radiante durante a inserção deste dispositivo ^(^ ^4^ ^)^ . Essas táticas e os resultados encontrados quanto ao número de inserções na primeira punção no estudo que utilizou a compressa morna (96,92%, n=63) ^(^ ^36^ ^)^ e o berço aquecido (77,72%, n=457) ^(^ ^4^ ^)^ demonstram a necessidade de realmente serem empregadas medidas para evitar a hipotermia do RN durante procedimentos prolongados como a inserção do PICC. 

 O IC-ECG associado à USG para visualização vascular foi considerado a melhor técnica de inserção na diminuição do mau posicionamento primário da ponta pelo *ranking* da metanálise em rede. Porém, deve-se considerar que os estudos que a utilizaram eram não aleatorizados, com risco de viés maior que moderado e certeza da evidência muito baixa e baixa. Assim, torna-se correto referir que a técnica mais efetiva na redução do risco do mau posicionamento primário tenha sido o IC-ECG isolado e o IC-ECG/USG foi a segunda melhor, ultrapassando o MRA modificado e o controle. Os outros desfechos mensurados nos estudos que empregaram IC-ECG/USG ^(^ ^28^ ^,^ ^37^ ^)^ também resultaram em estimativas com um grau de incerteza importante e sem significância estatística. Isso evidencia que o IC-ECG/USG, no que tange à prevenção de complicações associadas ao PICC, ou não é vantajoso na prática clínica para tal ou necessita da realização de estudos aleatorizados para verificação mais fidedigna da existência ou não de benefícios. 

 Entretanto, o uso de equipamentos para auxiliar na visualização e punção venosa pode ser útil, já que RNs possuem veias mais estreitas que são imaturas e vulneráveis a rompimentos ^(^ ^1^ ^,^ ^25^ ^-^ ^26^ ^)^ . Na investigação com MRA modificado, inclusive, houve insucesso na punção de 18 RNs para inserção do PICC por fragilidade venosa ^(^ ^40^ ^)^ . 

 A INS recomenda a utilização da USG para avaliar a veia que será puncionada quanto ao seu calibre (cateter não pode ultrapassar 45% do vaso), se há anormalidades como oclusões e trombose e para identificação do posicionamento da ponta do dispositivo ^(^ ^11^ ^)^ . Em uma investigação descritiva realizada por enfermeiras do Irã com 30 RNs, utilizou-se um aparelho de USG para avaliação e visualização vascular na punção para inserção do PICC. Houve aumento da taxa de sucesso na primeira (68% grupo USG X 60% grupo convencional) e segunda tentativa (50% x 40%), mas esta não foi estatisticamente significativa ^(^ ^53^ ^)^ . Pesquisa retrospectiva americana cujo objetivo foi descrever o uso da USG na inserção do PICC encontrou taxa de sucesso de 100% em uma amostra composta de 10 RNs com múltiplas tentativas de acesso vascular sem êxito ^(^ ^54^ ^)^ . 

 A técnica de inserção que utilizou o MRA modificado ^(^ ^40^ ^)^ reduziu o risco de complicações gerais e de mau posicionamento primário com significância estatística (baixa e moderada a muito baixa certeza da evidência, respectivamente). 

 Estudo retrospectivo realizado com 588 RNs obteve baixa prevalência de complicações associadas ao cateter (10,71%) e também realizou a mensuração até a junção esternoclavicular direita, como o estudo com MRA modificado ^(^ ^40^ ^)^ , afirmando que a medida tradicional acabava por posicionar a ponta do PICC muito profunda ^(^ ^4^ ^)^ . Assim, torna-se imperativo, quando se trata de RNs, realizar uma mensuração prévia da quantidade de cateter a ser introduzida baseada em marcos de referência anatômicos, distinta da recomendada pela INS para outras populações em membros superiores (sítio de punção a região esternoclavicular direita e terceiro espaço intercostal) ^(^ ^11^ ^)^ . 

 USGRT mostrou efetividade em reduzir o mau posicionamento primário da ponta e o aumento do tempo de permanência do cateter de modo significativo e baixa e moderada certeza nos achados, respectivamente. Não apresentou estimativas de efeito significantes para as demais complicações e obteve até estimativas contrárias, sem significância e muito baixa certeza em alguns casos, como complicações gerais, infecção e mau posicionamento secundário da ponta. Há de se considerar que os estudos que utilizaram esta técnica de inserção ^(^ ^38^ ^-^ ^39^ ^)^ foram realizados com RNs muito prematuros e prematuros extremos, cujas peculiaridades podem ter possibilitado tendência de aumento de algumas complicações ou ausência de redução significativa de outras, como ocorreu com as demais técnicas. Ademais, foram os estudos com menor tamanho amostral. 

 Uma coorte prospectiva acompanhou a inserção de PICC em membros inferiores de 166 RNs em seu grupo experimental com USGRT e comparou com 141 RNs nos quais não foi utilizada esta tecnologia. Assim como nesta revisão, verificou-se redução significativa do mau posicionamento primário da ponta na medida em que a necessidade de ajustes do cateter no grupo USGRT foi bem menor (10,84% x 65,95%, *p* <0,001) e não houve nenhuma diferença significativa entre os grupos nas demais complicações (flebite, oclusão, bacteremia e ruptura) ^(^ ^12^ ^)^ . 

 Metanálise recente também valida os resultados encontrados nesta revisão ao verificar que a USG é excelente para localizar a ponta do PICC em unidade neonatal quando comparada com raio x, com sensibilidade de 95,2% e especificidade de 71,4%, devendo-se combinar a radiografia quando a localização da ponta não for possível por esse método ^(^ ^55^ ^)^ . 

 Com os resultados negativos dos estudos que utilizaram a USGRT, percebe-se ser imperativa a instituição de protocolos ^(^ ^56^ ^-^ ^57^ ^)^ , bem como o treinamento adequado e a experiência dos profissionais que manipulam o equipamento ^(^ ^57^ ^)^ . Quando se trata de RNs e crianças, a necessidade desses quesitos foi apontada como uma desvantagem da USG, assim como as dificuldades de algumas unidades possuírem um aparelho disponível 24 horas por dia ^(^ ^57^ ^-^ ^59^ ^)^ . 

 Várias alterações têm sido realizadas nos materiais e procedimentos relacionados à inserção e manutenção do PICC em RNs e ao uso de tecnologias para auxiliar punção e, nesse sentido, o posicionamento central do PICC tem sido mais recomendado pela literatura atual ^(^ ^11^ ^,^ ^56^ ^,^ ^60^ ^-^ ^61^ ^)^ . Essas tecnologias objetivam reduzir taxas de complicações e, consequentemente, a dor, o sofrimento e a morbimortalidade dos RNs ^(^ ^60^ ^)^ . Contudo, em nenhum dos estudos brasileiros publicados recentemente ^(^ ^26^ ^,^ ^42^ ^,^ ^62^ ^)^ , observa-se o uso dessas tecnologias (IC-ECG, USGRT, USG para visualização vascular etc), o que nos leva a refletir sobre quais motivos têm contribuído para que elas não estejam sendo utilizadas e estudadas no Brasil. 

 Esta revisão sistemática e metanálise (pareada e em rede) verificou a efetividade das técnicas de inserção na prevenção da ocorrência de complicações relacionadas ao PICC em RNs, sendo que estas podem, inclusive, culminar em sequelas e óbito. Incluiu cerca de seis técnicas de inserção, mais de 1000 RNs em sua amostra, empregada com rigor metodológico e análises diferenciadas. Os estudos incluídos foram todos ensaios clínicos controlados aleatorizados e não aleatorizados, avaliados em conjunto, como já preconizado na literatura ^(^ ^63^ ^)^ , cuja maioria possuía tamanho amostral superior a 100 RNs. Tal fato pode ser considerado uma vantagem, pois os ensaios clínicos relacionados a cateteres intravenosos em RNs, geralmente, possuem amostra média menor que 100 participantes ^(^ ^64^ ^)^ . 

Contudo, esta revisão deve ser interpretada no contexto de algumas limitações. Uma pequena amostra de estudos comparativos foi avaliada, com possibilidade de realização de poucas metanálises de intervenção. Além disso, nenhum estudo comparou as diferentes tecnologias entre si para inserir o PICC, o risco de viés da maioria dos estudos incluídos foi julgado como “Algumas preocupações” e nenhum desfecho foi avaliado com certeza da evidência alta.

## Conclusão

Esta revisão demostrou que as técnicas de inserção são capazes de prevenir complicações relacionadas ao PICC em RNs, mas ainda não se pode afirmar qual é a mais efetiva para todas elas. Isso porque cada técnica incluída nesta revisão mostrou maior efetividade para diferentes complicações, ainda que o IC-ECG tenha reduzido a maior parte delas.

Todas as técnicas diferentes da punção guiada por marco de referência anatômico (controle) reduziram o mau posicionamento primário da ponta, mas o IC-ECG foi a mais efetiva para reduzi-lo juntamente com as complicações gerais, a flebite e o tempo de permanência do cateter, diminuindo também, de maneira considerável e significativa, as arritmias. A fórmula mostrou maior efetividade em aumentar o tempo de permanência do cateter e foi a segunda melhor em reduzir as complicações gerais. Também foi considerada superior com muito baixa certeza de evidência e sem significância estatística em diminuir o risco de infecção, mau posicionamento secundário da ponta e trombose. A USGRT não mostrou redução das demais complicações, aumentou o tempo de permanência do cateter e verificou tendência de aumento sem significância de algumas.

Há necessidade de mais ensaios clínicos controlados aleatorizados com maiores amostras que comparem as técnicas de inserção no que tange às taxas de complicações. Estudos de custo-efetividade também devem ser operacionalizados para que políticas públicas sejam elaboradas no sentido de reduzir as falhas na terapia intravenosa dos pacientes de todas as idades e proporcionar maior segurança. Todo esforço deve ser direcionado para que mais estratégias, técnicas e tecnologias sejam investigadas de modo que o uso do PICC, imprescindível para os RNs, torne-se mais seguro para esta população tão vulnerável.
